# Directed evolution of RebH for catalyst-controlled halogenation of indole C–H bonds†
†Electronic supplementary information (ESI) available: Complete experimental procedures and characterization are supplied as supporting information. See DOI: 10.1039/c5sc04680g


**DOI:** 10.1039/c5sc04680g

**Published:** 2016-02-19

**Authors:** Mary C. Andorfer, Hyun June Park, Jaylie Vergara-Coll, Jared C. Lewis

**Affiliations:** a Department of Chemistry , University of Chicago , Chicago , IL 60637 , USA . Email: jaredlewis@uchicago.edu

## Abstract

RebH variants capable of chlorinating substituted indoles *ortho*-, *meta*-, and *para*- to the indole nitrogen were evolved by directly screening for altered selectivity on deuterium-substituted probe substrates using mass spectrometry. This systematic approach allowed for rapid accumulation of beneficial mutations using simple adaptive walks and should prove generally useful for altering and optimizing the selectivity of C–H functionalization catalysts. Analysis of the beneficial mutations showed that structure-guided selection of active site residues for targeted mutagenesis can be complicated either by activity/selectivity tradeoffs that reduce the possibility of detecting such mutations or by epistatic effects that actually eliminate the benefits of a mutation in certain contexts. As a corollary to this finding, the precise manner in which the beneficial mutations identified led to the observed changes in RebH selectivity is not clear. Docking simulations suggest that tryptamine binds to these variants as tryptophan does to native halogenases, but structural studies will be required to confirm these models and shed light on how particular mutations impact tryptamine binding. Similar directed evolution efforts on other enzymes or artificial metalloenzymes could enable a wide range of C–H functionalization reactions.

## Abstract 2

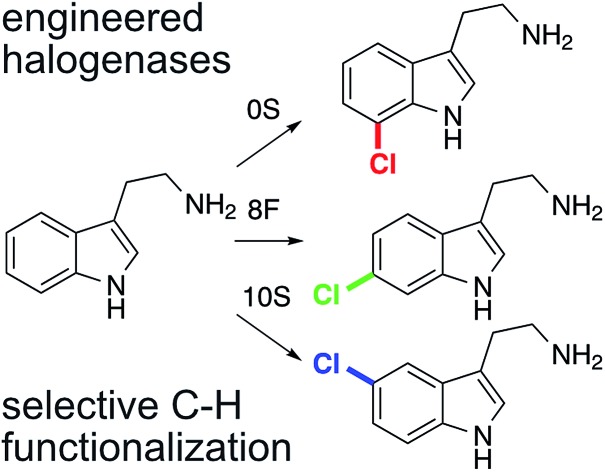
RebH variants capable of chlorinating substituted indoles *ortho*-, *meta*-, and *para*- to the indole nitrogen were evolved by directly screening for altered selectivity on deuterium-substituted probe substrates using mass spectrometry.

## Introduction

**Fig. 1:**
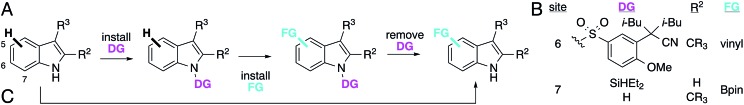
(A) Selective installation of functional groups (FG) on indoles *via* C–H bond activation using (B) different catalyst directing groups (DG) or (C) non-directed enzyme catalysis.

Catalytic C–H bond functionalization has the potential to reduce the need for functional group manipulation during chemical syntheses by allowing conversion of C–H bonds directly to functionality present in desired targets.^1^ This increases step economy, decreases waste, and expands the pool of substrates available for synthetic efforts.^2^ Organometallic catalysts dominate progress in this field,^1^ but most of these require substrates bearing particular functional groups termed directing groups for reactivity, selectivity, or both.^3^–^5^ While such groups may be present in a target molecule,^6^ often they are not, or their directing effects are mitigated by catalyst binding to other functionality in a substrate.^7^ In such cases, appropriate directing groups must be installed and removed, which decreases the benefits of C–H functionalization (Fig. 1A). The site selectivity of directed C–H functionalization is also intentionally limited; a given directing group enforces a particular selectivity on a substrate by design (Fig. 1B).^8^ In most cases, directing groups are used to functionalize proximal C–H bonds,^3^–^5^ but distal C–H bonds have been accessed using elaborate directing groups^9^ or catalysts with pendant functional group recognition elements.^10^,^11^ While some catalysts are capable of functionalizing C–H bonds without the need for directing groups,^12^ substrate-dependent steric,^13^ electronic,^14^–^16^ or stereoelectronic^17^–^19^ differentiation of C–H bonds is required for selectivity in these cases. Despite the synthetic utility of these methods,^2^,^8^,^20^ they highlight how catalyst control over the selectivity of C–H functionalization,^21^ and, just as importantly, the ability to tune that selectivity, remain fundamental challenges.

Many enzymes catalyze selective C–H functionalization by binding substrates such that a single C–H bond is presented to active site residues and cofactors involved in C–H cleavage.^22^ Indeed, the impact that C–H bond functionalization can have on synthetic efficiency is perhaps best appreciated by comparing natural product biosynthesis involving such enzymes and total syntheses using conventional methods.^23^ These catalysts evolved to functionalize particular substrates, but directed evolution^24^ provides a systematic approach for improving enzyme activity, selectivity, scope, and other properties.^25^ With the notable exception of cytochromes P450,^26^ however, few enzymes that functionalize C–H bonds have been evolved for biocatalysis.^22^ Even in cases where enzymes have been engineered for selective C–H functionalization, no selective pressure was applied to alter their selectivity; active variants were identified, and their selectivity was determined post hoc.^26^–^28^

Here, we show that the selectivity of rebeccamycin halogenase (RebH) can be evolved using deuterium-substituted probe substrates in combination with a mass spectrometry assay. Our results constitute a rare example of catalyst optimization to enable C–H functionalization *ortho*, *meta*, and *para* to an aromatic substituent with high selectivity (Fig. 1C, FG = Cl). This was accomplished without the use of metals or the harsh conditions typically associated with aromatic halogenation; RebH is an FADH_2_-dependent halogenase (FDH) that uses halide salts as a halogen source and O_2_ as an oxidant. The generality of the evolution strategy and the selectivity assay used in this effort suggest that the selectivity of other enzymes could be evolved in a similar fashion to enable a range of non-directed C–H functionalization reactions.

## Results and discussion

RebH catalyzes 7-halogenation of tryptophan.^29^ This process involves the reaction of O_2_ with bound FADH_2_ to form a flavin peroxide that oxidizes halide anion (X^–^, X = Cl, Br) to the corresponding hypohalous acid (HOX). HOX is proposed to travel through a pore within the enzyme to the active site where it has been shown to react with K79 to form a haloamine species.^30^ Aromatic halogenation is believed to proceed *via* electrophilic aromatic substitution of enzyme-bound substrate by a proximal halenium ion (X^+^) donor.^31^ This species is proposed to be either the K79 haloamine^30^ or HOX,^31^,^32^ the latter presumably regenerated *via* haloamine hydrolysis and hydrogen bonded within the active site.

We established that RebH halogenates a range of substituted indoles and electron rich aromatic substrates^33^ and evolved variants of this enzyme with improved stability^34^ and expanded substrate scope.^35^ While variants with high selectivity for a single site on different substrates were readily identified from these efforts, variants with different selectivities on a particular substrate were rarely observed. Related enzymes that chlorinate the 6- and 5-positions of tryptophan have also been characterized, however (Thal and PyrH, respectively),^36^ suggesting that it should be possible to alter RebH selectivity. Furthermore, site-directed mutagenesis of the 7-halogenase PrnA led to a variant that provided a 1 : 2 mixture of 5- and 7-chlorotryptophans,^37^ and a similar approach was used to alter the selectivity of PrnA toward 2-aminobenzoic acid so that 5-chlorination was favored over 3-chlorination (from 84 : 16 for PrnA to 38 : 62).^38^ While these examples show that halogenase selectivity can be altered, low selectivities were observed, and an initial examination of PrnA substrate scope^39^ indicated that substituted indoles were chlorinated on the pyrrole ring. Given the ability of RebH variants to halogenate the less reactive benzene ring of indole substrates^33^ and our success in engineering this enzyme,^33^,^35^ we initiated an effort to evolve its selectivity toward indoles. The broad utility of substituted indoles has led to the development of a number of metal-catalyzed methods for functionalizing indole C–H bonds. Most of these target the more reactive pyrrole ring,^40^ but directing and blocking group strategies have been used to access the indole benzene ring.

For example, an *N*-silyl directing group was used to borylate the 7-position of 2-unsubstituted indoles *via* a 3-step sequence (Fig. 1A/B).^41^ Substrates bearing a substituent at the indole 2-position have been alkenylated at the 6-position using a similar directing group approach^42^ and borylated at the 7-position using only the indole nitrogen as a directing group^43^ (Fig. 1A/B). Very recently, an *N*-silyl blocking group was used to borylate the 6-position of 3-substituted indoles, although significant 5-borylation was also observed.^44^ RebH variants capable of halogenating the benzene ring of 1,2-unsubstituted indoles would thus illustrate the potential for enzymes to eliminate the need for directing/blocking groups typically required for selective C–H functionalization (Fig. 1C). Halogen substituents are known to greatly impact the biological activity of small molecules^45^,^46^ and can be used for subsequent cross-coupling reactions to access additional functionality,^47^,^48^ making halogenation a particularly useful process. More broadly, successful evolution of RebH selectivity would establish a general approach for evolving the selectivity of other FDHs,^36^ each of which has its own unique selectivity and substrate scope. This, in turn, would provide access to a range of engineered halogenases for late stage C–H functionalization of synthetic intermediates, natural products, and other biologically active compounds.

### MALDI-MS as a screen for selectivity

**Fig. 2:**
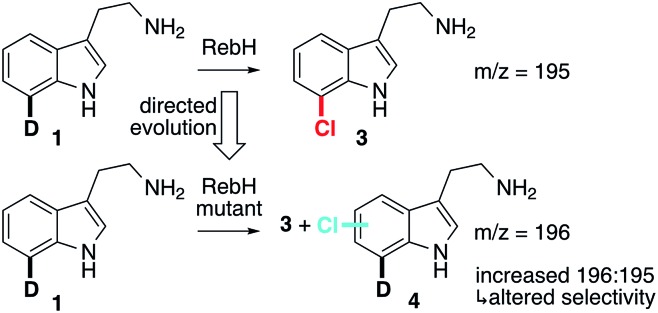
Mass spectrometry assay for halogenase selectivity using probe substrate **1**.

Engineering RebH variants with altered selectivity requires an assay capable of differentiating halogenated product isomers regardless of the site of halogenation. Li and coworkers reported a method to determine the enantioselectivity of C–H/D hydroxylation reactions conducted on deuterium-substituted substrates using mass spectrometry (GC/MS or LC/MS).^49^ We envisioned that deuterated tryptamines could be used in a similar fashion to identify RebH variants with altered regioselectivity. RebH halogenates tryptamine with the same high 7-selectivity that it exhibits on tryptophan,^33^ but deuterated tryptamines are more readily prepared than the corresponding tryptophans. We therefore prepared 7-deuterotryptamine, **1**, as a probe substrate since any alternate regioselectivity would lead to products (4) with *m*/*z***1** unit higher than that associated with the native regioselectivity (**3**, Fig. 2). Given the high selectivity of RebH on tryptamine, we believed that this unbiased probe would provide the best opportunity for identifying altered selectivity that could then be optimized using site-specific probes (*e.g.* 5-deuterotryptamine, **2**, *vide infra*). No significant kinetic isotope effect was observed for RebH-catalyzed chlorination of d_5_-tryptophan (Fig. S1 and S2†), so rate differences of the isotopomers did not have to be taken into account.^49^ Halogenation selectivity could be reliably determined by MALDI-MS analysis of crude reaction mixtures arrayed onto a standard 384-spot sample targets, which allowed for rapid evaluation of halogenase libraries.

### Rounds 0–6: altering RebH selectivity using probe **1**

**Fig. 3:**
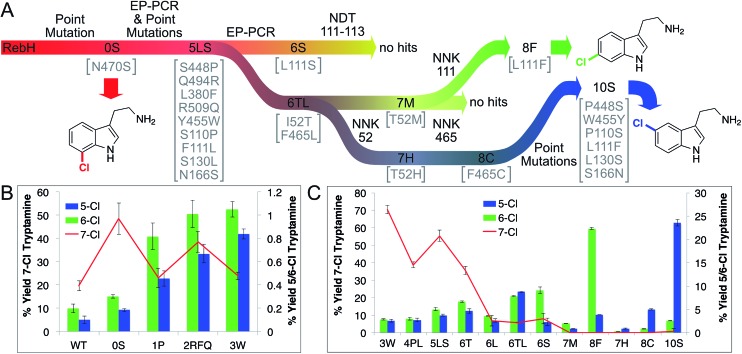
(A) Lineage diagram showing mutagenesis methods and mutations found in selected variants above and below the lineage arrows, respectively. (B/C) Yield of 7- (left *y*-axis) and 6- and 5-chlorotryptamine (right *y*-axis) for different variants along the halogenase lineage. Reactions conducted using 2.5 μM MBP-RebF, 9 U mL^–1^ GDH, 100 mM NaCl, 20 mM glucose, 100 μM NAD and FAD, 0.5 mM phenol, 0.5% v/v *i*-PrOH/25 mM HEPES buffer, pH 7.4, 25 °C. Substrate and enzyme concentrations: (B) 1.5 mM **2**, 15 μM RebH variant. (C) 0.5 mM **2**, 25 μM RebH variant.

Tryptamine chlorination by several RebH variants developed in our laboratory^34^,^35^ was examined to identify a suitable parent for our selectivity evolution effort (summarized in Fig. 3A). This analysis revealed that variants containing the mutation N470S provided the highest chlorination yields, which were required to maintain a MS signal-to-noise ratio sufficient for analysis of reactions conducted in cell lysate. Introducing this mutation into RebH gave variant 0S, which provided the highest chlorination yields of all variants evaluated (2.5-fold higher than RebH, >99% 7-chlorination, Fig. 3B). Despite the high selectivity of these enzymes for 7-chlorination, a trace amount of an additional chlorinated species was also detected by LC-MS, and authentic standards were used to establish that this was 5- and/or 6-chlorotryptamine (chromatographic separation of these compounds was not possible). We believed that this activity, while minor, would be sufficient to enable evolution of enzymes with high selectivity for both of these positions. A library of 0S variants was therefore generated using error-prone PCR, the library was expressed in *Escherichia coli*, and chlorination of **1** using lysates from 1000 clones was evaluated using an automated MALDI-MS method. The ratio of **4** (*m*/*z* = 196) to **3** (*m*/*z* = 195) was calculated for each reaction, and hits were defined as those with 196/195 ratios higher than that of parent. This led to the identification of variant 1P (0S-S448P), which provided a 4.5-fold increase in 5/6-chlorination selectivity, indicating that RebH selectivity could be altered *via* random mutagenesis and screening. 1P was used as the parent for a second round of error-prone PCR and screening as described above. Two variants provided increased yields of 5/6-chlorotryptamines, and these mutations were combined to give variant 2RFQ (1P-Q494R, L380F, R509Q, Fig. 3B).

O'Connor and coworkers previously showed that the mutation Y455W improved the specificity of RebH for tryptamine over tryptophan without changing selectivity for 7-chlorination.^50^ Introducing this mutation into 2RFQ to generate 3W, on the other hand, both decreased 7-chlorination and increased 5/6-chlorination (Fig. 3B). Error-prone PCR of 3W followed by recombination of beneficial mutations led to variant 4PL (3W-S110P, F111L), which further decreased 7-chlorination (Fig. 3C). To improve overall conversion, a number of mutations previously found to improve the stability of RebH^34^ were individually introduced into 4PL. Beneficial mutations were again combined to generate variant 5LS (4PL-S130L, N166S), which was used as a parent for another round of error-prone PCR and MALDI screening. Three variants from this library, each containing an active site mutation, were found to increase 5/6-chlorination to levels approaching or exceeding that of the residual 7-chlorination (6S, 5LS-L111S; 6T, 5LS-I52T; 6L, 5LS-F465L). To distinguish, and thus provide a means to individually optimize 5- and 6-chlorination, site-selective probe **2**, 5-deuterotryptamine, was prepared. LC-MS analysis of reactions conducted using **2** can be used to determine 7-, 6-, and 5-chlorotryptamine yields *via* chromatographic separation of the 7- and 5/6-isomers and mass differentiation of the 5- and 6-isomers (Fig. S12†). This procedure revealed that 6S provided 47% selectivity for 6-chlorotryptamine. Similar analysis of 6T, 6L, and variants resulting from recombination of L111S, I52T, and F465L indicated that variant 6 TL (5LS-I52T, F465L) provided the highest selectivity for 5-chlorotryptamine (39%) of all mutants screened (Fig. 3C).

### Evolving 5- and 6-halogenases using probe **2**

Despite the significant improvement in aromatic chlorination selectivity and high tryptamine conversion in reactions catalyzed by 6S, isolation of the products from these reactions revealed that 2-oxotryptamine was also being formed. Analysis of the halogenase lineage indicated that this product only formed to a significant extent after the F111S mutation was introduced into 5LS to give 6S. Since variants 6T (5LS-I52T) and 6L (5LS-F465L) also showed significant 5- and 6-chlorination without oxotryptamine formation, individual randomization of residues 52 and 465 in 6TL by site directed mutagenesis with NNK codons was pursued as a means to further optimize chlorination selectivity (Fig. 3A).

The resulting libraries were screened for activity on **2** by sequential MALDI-MS/UPLC to determine 7-, 6-, and 5-chlorotryptamine yields (see ESI†). Several hits were identified, including 7M (6TL-T52M) and 7H (6TL-T52H), which possess improved selectivity for 6- and 5-chlorination, respectively. Degenerate NNK codons were then introduced at residue 465 of these variants, and while no improvements were observed in the 7M library (Fig. 3A), variant 8C (7H-F465C) provided 86% selectivity for 5-chlorination (up from 39% with 6TL). Despite the improved selectivity of 7M and 8C, both of these variants provided low product yields. Given the significant impact of residue 111 on both halogenase activity and selectivity, we examined the effects of mutating this residue in both 7M and 8C (Fig. 3A). Site directed mutagenesis of residue 111 in 7M using an NNK codon was used to randomize this site. Remarkably, the variant with the highest selectivity for 6-chlorination (85%) as well as the highest yield of 6-chlorotryptamine (11-fold increase over 7M) from this library, 8F (7M-L111F), resulted from reversion of the F111L mutation that originally led to a significant change in 7-selectivity of 3W. Given this finding, we reverted this same mutation and several additional mutations in 8C to generate 10S, which led to a 5-fold improvement in yield while maintaining its high selectivity for 5-chlorination (87%).

### Isolated yields, kinetics, and substrate scope

**Fig. 4:**
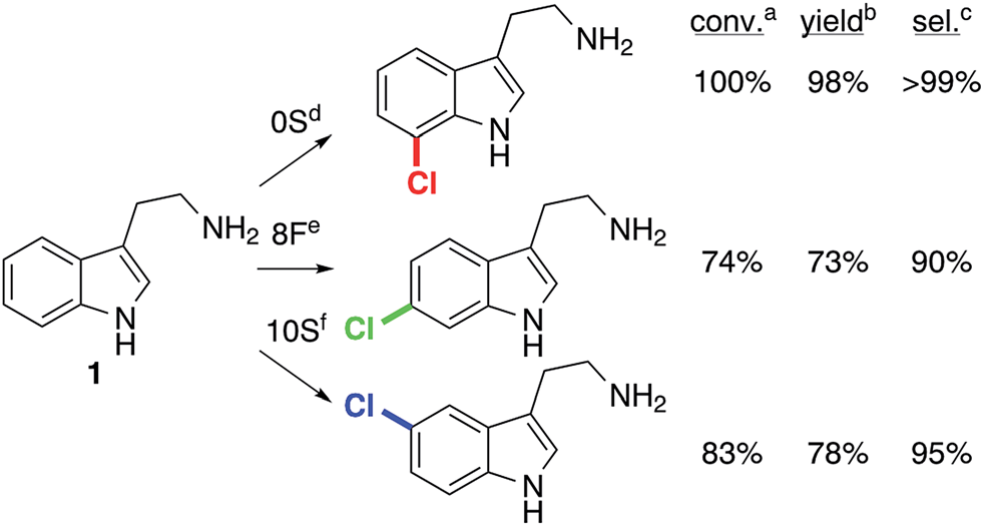
Chlorination of tryptamine using engineered halogenases. ^a^Conversion of starting material determined by UPLC analysis of crude reaction mixtures. ^b^Isolated yield of pure product. ^c^Selectivity determined by NMR analysis of a purified mixture of isomers (inseparable by preparative chromatography). ^d^10 μM 0S, 0.5 mM tryptamine (10 mg), 2.5 μM MBP-RebF, 9 U mL^–1^ GDH, 10 mM NaCl, 20 mM glucose, 100 μM NAD and FAD, 0.5% v/v *i*-PrOH/25 mM HEPES buffer, pH 7.4, 25 °C. ^e^As in (d) but 50 μM 8F, 16 °C. ^f^As in (d) but 50 μM 10S, 100 mM NaCl, 10 °C.

Optimization of reaction conditions to maximize product yields using 0S, 8F, and 10S indicated that 8F and 10S gave higher yields at lower temperatures (16 and 10 °C, respectively) and that 100 mM NaCl further increased yields in reactions catalyzed by 10S. While higher rates were observed for 8F and 10S with higher substrate concentrations, 0.5 mM substrate was used to maximize conversion rather than total turnover numbers. The selectivity of these enzymes remained essentially unchanged despite these variations. Tryptamine chlorination reactions (10 mg) were then conducted using the optimal conditions and loading for each enzyme (Fig. 4). Good yields (73–98%) and high selectivities (90–100%) were obtained.

The catalytic efficiencies for 0S, 10S and 8F were compared by steady state kinetic analysis. The enzyme loading requirements for these reactions are reflected in the kinetic parameters, with 0S maintaining a significantly higher *k*_cat_/*K*_m_ than 10S and 8F (Table 1). This loss in catalytic efficiency is partially due to a reduction in *k*_cat_, although both 5- and 6-halogenases display slightly higher *k*_cat_ values than RebH.^33^ The *K*_M_ of the 7-halogenase matched that of RebH,^33^ while the mutants with non-native selectivity, 10S and 8F, displayed substantially higher *K*_M_ values, suggesting weaker substrate binding. While substrate inhibition has been observed previously for tryptophan halogenases,^31^ this was not seen for either 8F or 10S at the concentrations investigated (up to 2.5 and 4.5 mM, Fig. S16–20†).

**Table 1:** Kinetic parameters for RebH, 0S, 8F, and 10S^*a*^

Enzyme	*K*_m_ (μM)	*k*_cat_ (min^–1^)	*k*_cat_/*K*_m_ (min μM)^–1^
RebH^*b*^	9	0.023	2.6 × 10^–3^
0S	10.6	0.135	2.6 × 10^–2^
8F	1747	0.037	2.1 × 10^–5^
10S	160	0.028	1.8 × 10^–4^

^*a*^2–4500 μM tryptamine, 2.5 μM MBP-RebF, 9 U mL^–1^ GDH, 100 mM NaCl, 20 mM glucose, 100 μM NAD and FAD, 0.5 mM phenol, 2.5% v/v DMSO/25 mM HEPES buffer pH 7.4, 25 °C. 0.1 μM 0S, 25 μM 10S, 25 μM 8F. Time points collected from 10–60 minutes.

^*b*^Values taken from a previous study.^33^

Given that RebH halogenates (X = Cl, Br) a number of indole derivatives with high selectivity,^33^ the activity of 8F and 10S was evaluated on several compounds (Table 2), including 2-methyltryptamine (entry 3), *N*-methyltryptamine (entry 4), tryptophol (entry 5), and tryptophan. 8F provided as good or better selectivity for 6-chlorination of these substrates than it did on tryptamine while providing reasonable to excellent yields. On the other hand, 10S had essentially perfect selectivity for 5-chlorination of *N*-methyltryptamine and good selectivity for 5-chlorination of tryptophol but low selectivity on 2-methyltryptamine (reasonable to good yields were again observed).

**Table 2:** Conversion and selectivity for halogenation of different substrates using 8F and 10S

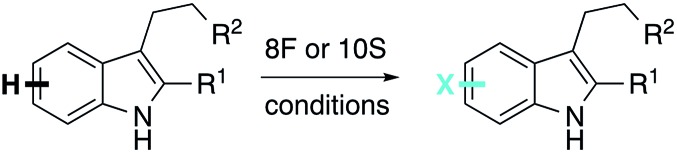							
Entry	R^1^	R^2^	X	8F (6-halogenase)^*a*^		10S (5-halogenase)^*b*^	
				Conv. (%)^*c*^	6-X (%)^*d*^	Conv. (%)^*c*^	5-X (%)^*d*^
1^*e*^	H	NH_2_	Cl	74	90	83	95
2	H	NH_2_	Br	84	69	35	59
3	Me	NH_2_	Cl	97	99	77	24
4	H	NHMe	Cl	54	98	74	>99
5	H	OH	Cl	48	89	48	84

^*a*^50 μM 8F, 0.5 mM substrate (1–2 mg), 2.5 μM MBP-RebF, 9 U mL^–1^ GDH, 10 mM NaCl, 20 mM glucose, 100 μM NAD and FAD, 0.5% v/v *i*-PrOH/25 mM HEPES buffer pH 7.4, 16 °C.

^*b*^Same as in (a) but with 50 μM 10S and 100 mM NaCl.

^*c*^Conversion determined by UPLC.

^*d*^Selectivity determined by NMR analysis of a purified mixture of inseparable isomers (X = Cl) or by LCMS analysis of reactions conducted using probe **2** (X = Br).

^*e*^Data from preparative reaction (Fig. 4).

Neither 8F nor 10S catalyzed chlorination of tryptophan (0.5 mM), the native substrate of RebH (Fig. S27†). This lack of activity can be rationalized for 8F since it includes the Y455W mutation known to improve RebH specificity for tryptamine over tryptophan,^50^ but the absence of this mutation in 10S makes the origin of its altered substrate specificity less clear. In addition, while both 8F and 10S catalyze tryptamine bromination, only modest selectivity was observed (Table 2, entry 2). These findings contrast with our previous results showing that engineered RebH variants typically maintain activity on tryptophan^35^ and catalyze chlorination and bromination with similar selectivity.^33^ On the other hand, an initial investigation of 10S substrate scope revealed that it chlorinates several additional substrates and provides altered product distributions on these substrates relative to RebH (Fig. S28†). These data show that 10S and 8F, which were evolved for altered selectivity on tryptamine, can halogenate substrates other than tryptamine with altered selectivity. While deviations from expected activity on indoles can result from minor structural differences, even major structural differences are tolerated in many cases.

The novel scope and selectivity of halogenases along our selectivity lineage make these enzymes promising catalysts for late stage halogenation^51^ and metabolic engineering.^50^,^52^ Achieving high isolated yields in larger-scale reactions (>10 mg), on the other hand, will require significant improvements in the activity of these enzymes. While the focus of this study was changing RebH selectivity, our data show that activity can also be improved without decreasing selectivity. At several points in the selectivity lineage (WT-0S, 1P-2RFQ, 8C-10S, and 7M-8F), the percent yield of the major chlorotryptamine isomer (7, 6, or 5-Cl) was significantly improved (1.7–11 fold) while improving or not affecting selectivity (Fig. 3B/C and S29†). This finding is consistent with our previous evolution efforts in which activity on non-native substrates was improved without sacrificing the observed selectivity.^35^ Sewald has also shown that cross-linked RebH can be used to halogenate substituted tryptophans on gram scale.^53^ These approaches to improving halogenase activity and reaction scale, coupled with our method for evolving halogenase selectivity, provide a general framework for improving halogenases for selective catalysis.

### Tryptamine halenium affinity

As previously noted, RebH catalysis is believed to involve electrophilic aromatic substitution of enzyme-bound substrate by a proximal halenium ion donor, believed to be either a K79 haloamine or HOX.^30^–^32^ The observed selectivity of 0S, 8F, and 10S toward 3-substituted indoles shows that these enzymes can differentiate similarly reactive sites on indole benzene rings both from one another and from the more reactive indole pyrrole ring.^39^,^54^ Similar selectivity preferences are a hallmark of tryptophan halogenation by native FDHs,^36^ but a quantitative evaluation of halenium ion reactivity toward different substrate sites, and thus the extent to which FDHs override the chemoselectivity of substrates toward halenium ion donors, such as HOX or haloamines, has not been reported. Calculated halenium affinity (HalA) has been used to predict the reactivity of a wide range of substrates toward halenium ion donors.^55^ The calculated HalA values (X = Cl) for the 2-, 4-, 5-, 6- and 7-positions of tryptamine were 177, 166, 163, 166, and 161 kcal mol^–1^, respectively (see ESI†). By this measure, 0S chlorinates the least reactive site on tryptamine, 8F and 10S selectively chlorinate sites that differ in reactivity by only 3 kcal mol^–1^, and all three enzymes chlorinate sites substantially less reactive than the 2-position. All of the engineered halogenases therefore override the expected halenium ion chemoselectivity toward tryptamine, but the ability of 8F and 10S to accomplish this feat is particularly notable given their relatively weak substrate binding (Table 1). Assuming *K*_d_ can be approximated by *K*_M_ for these enzymes,^56^ the Δ*G* for tryptamine binding to 8F and 10S is only 3.8 and 5.2 kcal mol^–1^, respectively, showing that even relatively weak binding can overcome large differences (>10 kcal mol^–1^) in HalA.

### Tryptophan binding and tryptamine docking

**Fig. 5:**
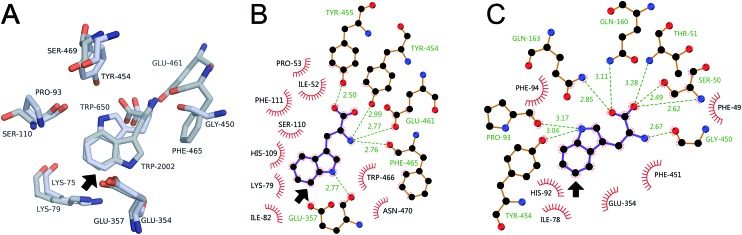
(A) Key residues in the RebH (grey carbons) and PyrH (light blue carbons) active sites. Interactions involved in tryptophan binding in (B) RebH and (C) PyrH. Arrow denotes chlorination site.

Better understanding of how substrate binding in 0S, 8F, and 10S might control halenium selectivity can be gleaned from previous work on the selectivity of RebH- and PyrH-catalyzed tryptophan chlorination.^57^ Aligning the structures of the RebH–58 and PyrH–tryptophan^57^ complexes (Fig. 5A) shows that the chlorinated sites (RebH, C_7_–H; PyrH, C_5_–H) lie at nearly the same point and roughly within a plane that bisects the space between conserved active site lysine and glutamate residues (RebH, K79/E357; PyrH, K75/E354). These residues have been proposed to either bind and activate HOX,^32^ or form a reactive chloramine and serve as a general base, respectively,^30^ to enable electrophilic aromatic substitution of the tryptophan benzene ring. Regardless of the nature of the halogen electrophile, its location proximal to the tryptophan C–H bond halogenated by each enzyme provides a rationale for the observed selectivity.^31^

The interactions involved in tryptophan binding are thus central to the ability of RebH and PyrH to orient this substrate to control halogenation selectivity.^57^ In both enzymes, tryptophan is bound *via* extensive hydrogen bonding interactions to the amino acid moiety (and ion pairing between the amine and E461 in RebH) (Fig. 5A), conserved aromatic residues that sandwich the benzene ring (H109/F111 in RebH; H92/F94 in PyrH), and hydrogen bonding between the indole N–H and backbone amide carbonyl moieties (E357 in RebH; P93 in PyrH). Because P93 is located on the opposite side of the PyrH active site relative to E357 in RebH, P93 hydrogen bonding leads to a flipped orientation of the indole moiety in PyrH. This difference in orientation determines whether C_7_–H or C_5_–H bond projects toward the conserved active site lysine and glutamate residues and undergoes halogenation. Notably, however, both RebH and PyrH possess backbone amides suitable for N–H hydrogen bonding in either orientation (C_7_–H: E357/E354; C_5_–H: S110/P93). Additional interactions, including π-stacking between tryptophan and W466 in RebH and a second indole N–H hydrogen bond to Y454 in PyrH, have been proposed to favor the substrate orientation observed for each enzyme.^57^

With these aspects of tryptophan–RebH and –PyrH binding in mind, docking simulations were used to identify binding interactions in tryptamine poses consistent with the FDH mechanism and selectivity of 0S, 8F, and 10S. Mechanistically relevant poses were taken to be those in which the tryptamine indole binds in a planar orientation between H109/F111 with an aromatic C–H bond at the site occupied by the tryptophan C_7_–H bond in the tryptophan–RebH complex (Fig. 5A/B).^57^ To validate this approach, AutoDock Vina^59^ was used to dock tryptophan into an apo RebH structure minimized using the GROMOS 43B1 force field in Swiss-PDBViewer.^60^ The lowest energy poses were consistent with the binding observed in the crystal structure of the RebH–tryptophan complex (Fig. S23†),^58^ although higher energy poses with tryptophan bound in flipped orientations (consistent with 5-halogenation^57^) were also obtained.

**Fig. 6:**
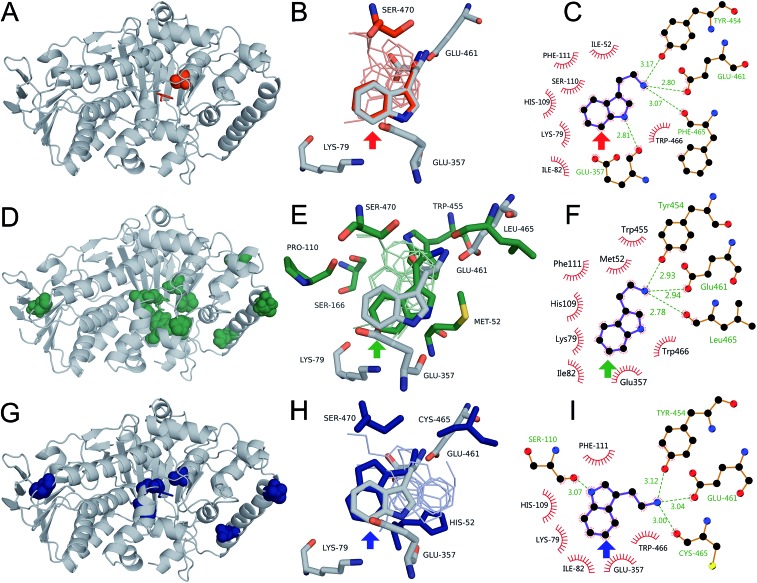
Location of mutations and tryptamine poses for 0S (red), 8F (green), and 10S (blue) mapped onto the RebH structure (grey). (A, D, and G) Location of mutations (spheres) and tryptamine poses (sticks). (B, E, and H) Active site mutations, conserved residues, tryptamine poses consistent with observed selectivity, and native tryptophan pose (sticks) and additional tryptamine poses (lines). (C, F, and I) Binding interactions in poses consistent with observed selectivity. Colored arrows indicate the chlorination site.^61^

Swiss-PDBViewer was then used to minimize the structures of 0S, 8F, and 10S, tryptamine was docked into each of the structures, and the resulting binding poses were analyzed. For each variant, a pose consistent with the observed selectivity was obtained, but poses consistent with alternate selectivities were again also obtained. Indeed, structures consistent with 5-, 6-, and 7-halogenation were obtained for 0S, which contains only a single point mutation (N470S) relative to RebH (Fig. S24†). The poses consistent with 7- (the observed selectivity; Fig. 6B) and 5-halogenation were essentially identical to those obtained from docking tryptamine in RebH and analogous to those found in the RebH–58 and PyrH–tryptophan^57^ structures (Fig. S25†). The pose consistent with 6-halogenation lacks both the indole N–H hydrogen bonding and amine ion pairing interactions observed in all tryptamine or tryptophan poses consistent with 7- or 5-halogenation. It is rotated, rather than flipped, within the 0S active site to project C_6_–H toward K79/E357. This rotation is apparently enabled by N470S, which allows formation of a hydrogen bond between the tryptamine amine and the backbone carbonyl of S110. Interestingly, the pose consistent with the selectivity of the 6-selective halogenase 8F did not involve this mode of amine binding and instead appeared largely similar to the 7-halogenation pose, but rotated so that C_6_–H projected toward K79/E357 (Fig. 6E).

A flipped pose in which both C_6_–H and C_5_–H projected toward K79/E357 (Fig. S26†) was also obtained. No crystal structure of a tryptophan 6-halogenase has been reported, so further structural characterization of 8F and native 6-halogenases, including Thal,^36^ will be required to determine the relevance of a flipped or rotated orientation to 6-selective halogenation. Only one mechanistically relevant pose was generated for the 5-halogenase 10S, and this was consistent with 5-halogenation (Fig. 6H). This pose was similar to the flipped tryptamine poses described for RebH and 0S docking (Fig. S24†), involving indole N–H hydrogen bonding to S110 and ion pairing with E461, and to tryptophan binding in the PyrH–tryptophan crystal structure.^57^

### Directed evolution strategy and library methods

While tryptamine docking provides binding poses consistent with the selectivities of 0S, 8L, and 10S, in no case is the precise mechanism by which mutations in these variants favor the relevant pose clear. It is possible that I52M and I52H in 8F and 10S disrupt tryptamine N–H hydrogen bonding to E357 due to their proximity to this residue, allowing additional mutations (*e.g.* P110/L465 in 8F and C465 in 10S) to alter binding. Characterization of these variants and their tryptamine complexes by X-ray crystallography and molecular dynamics simulations is underway to better understand structural perturbations resulting from mutagenesis. The lack of a clear mechanism by which random mutations improve fitness is a common theme in directed evolution, however, even when structural data for evolved variants are available.^62^ The same subtleties that complicate such analysis lead to the difficulty of rationally introducing specific mutations to improve enzyme function.^24^ Indeed, earlier attempts to modify the selectivity of PrnA toward tryptophan^37^ or substituted benzenes^38^ by mutating active site residues led to enzymes with poor selectivity, and active site mutations introduced into RebH to alter its specificity from tryptophan to tryptamine did not change its selectivity.^50^

On the other hand, halogenases with high non-native selectivity were obtained *via* random mutagenesis and screening using an assay for altered selectivity, followed by saturation mutagenesis of residues that significantly impacted selectivity or activity (Fig. 3A). Docking simulations suggest that tryptamine binding in 0S and 10S is similar to tryptophan binding in RebH and PyrH, respectively (Fig. 5).^57^ While evolving RebH selectivity took only a handful of mutations, however, PyrH and the 6-halogenase Thal differ from RebH by 205 and 335 residues, respectively, highlighting how dramatically different solutions to similar selectivity problems (*e.g.* 5- or 6-chlorination of indoles) can arise from homologous enzymes.

Despite the relatively small number of mutations required to convert RebH to a 5- or 6-halogenase, identifying these mutations required up to ten rounds of mutagenesis and screening. Several of the mutations identified are in the RebH active site (Fig. 6), and mutation of residues 52 and 465 in particular led to major branch points in the selectivity lineage (at 5LS and 6TL, Fig. 3A), suggesting that targeted mutagenesis of these sites could have decreased the effort required to alter RebH selectivity. We therefore compared the effects of introducing I52T and F465L into both RebH and 5LS (Table 3). The first of these mutations, I52T, decreases the 5/6-selectivity of RebH (1.4–1.5 fold), but, as shown in Fig. 3, it significantly improves 5/6-selectivity in 5LS (1.7–2.2 fold). While F465L increases 5/6-selectivity in both RebH and 5LS, the fold improvement for 5- *versus* 6-selectivity is opposite in the two cases (favoring 5Cl in RebH and 6Cl in 5LS) and no greater than the improvement afforded by the S448P mutation (variant 1P) in our selectivity lineage (Fig. 3). We next introduced the mutations at residues 52 and 465 that led to optimal 5- and 6-selectivity (I52H/F465C and I52M/F465L, respectively) into RebH. No measureable activity was observed for RebH-I52H/F465C, and while RebH-I52M/F465L did have altered selectivity (Fig. S22†), extremely low conversion (<0.5% at 5 mol% enzyme loading) was observed. Together, these results show that several of the key mutations responsible for the selectivity of 8F and 10S have a minimal or even negative impact on the 5/6-selectivity of RebH itself.

**Table 3:** Effects of mutations at residues 52 and 465 on RebH and 5LS on the selectivity of aromatic chlorination (SD, *n* = 2)

	RebH variant			5LS variant		
	No mutation	I52T	F465L	No mutation	I52T	F465L
% 7Cl	99.0 (0.10)	99.3 (0.05)	96.9 (0.24)	86.8 (0.12)	73.6 (0.20)	32.3 (0.98)
% 6Cl	0.7 (0.05)	0.5 (0.04)	1.5 (0.12)	7.4 (0.09)	16.6 (0.34)	49.2 (2.12)
% 5Cl	0.3 (0.05)	0.2 (0.09)	1.6 (0.12)	5.8 (0.03)	9.8 (0.14)	18.5 (1.14)

## Conclusions

RebH variants 0S, 8F, and 10S, which chlorinate substituted indoles *ortho*-, *meta*-, and *para*- to the indole nitrogen, were evolved by directly screening for altered selectivity on deuterium-substituted probe substrates using mass spectrometry. This systematic approach allowed for rapid accumulation of beneficial mutations using simple adaptive walks and should prove generally useful for altering and optimizing the selectivity of C–H functionalization catalysts. Analysis of the selectivity lineage showed how “rationally” selecting active site residues for targeted mutagenesis could be complicated either by activity/selectivity tradeoffs that reduce the possibility of detecting such mutations or by epistatic effects that actually eliminate their benefits in certain contexts.^62^ As a corollary to this finding, the precise manner in which the beneficial mutations improved RebH selectivity is not clear. Docking simulations suggest that tryptamine binds to these variants as tryptophan does to native halogenases, but structural studies will be required to confirm these models and shed light on how the mutations identified impact tryptamine binding.

Interestingly, 8F and 10S bind tryptamine rather poorly, but still chlorinate this substrate with almost exclusive selectivity for the 6- and 5-positions, respectively, rather than the RebH-preferred 7-position or the more reactive 2-position. Similar selectivity was observed for chlorination of 2-methyltryptamine, *N*-methyltryptamine, and tryptophol by 8F and 10S. These results indicate that even weak substrate binding can be sufficient to enable highly selective C–H functionalization in an enzyme active site,^63^–^65^ and directed evolution provides a means to systematically tune this binding to functionalize different C–H bonds on a given substrate. Similar efforts on other enzymes^22^ or artificial metalloenzymes^66^ that catalyze C–H functionalization could therefore enable a wide range of transformations.

## Abbreviations

FDH: Flavin dependent halogenase
MBP: Maltose binding protein
GDH: Glucose dehydrogenase
NAD: Nicotinamide adenine dinucleotide
FAD: Flavin adenine dinucleotide

## Supplementary Material

Supplementary information
