# Supplementary material for: Directed evolution of RebH for catalyst-controlled halogenation of indole C–H bonds
Source: Chem Sci. 2016 Feb 19;7(6):3720–9. doi: 10.1039/c5sc04680g (PMC4917012; doi:10.1039/c5sc04680g)
Supplement: Supplementary file 1 [file SC-007-C5SC04680G-s001.pdf]

This journal is © The Royal Society of Chemistry 2016

Table of Contents	Page
Materials	2
General Procedures	3-10
Library construction and screening	3-7
Expression and purification of RebH and MBP-RebF	8
Preparative bioconversions	9
Table of primers	10
Detailed Experimental Procedures	11-51
KIE experiments	11-13
MALDI MS as a screening method	13-15
Deuterated probe synthesis	16-20
Rounds 0-10	20-28
Conversion and selectivity determination of lineage	28-32
Kinetic analysis	32-36
Detailed isolation and characterization	37-38
Substrate scope characterization	38-40
Effect of key selectivity-changing residues on wtRebH	40-42
Tryptamine halenium affinity calculations	42-44
Docking studies	44-47
Additional Substrate Scope Profile for wtRebH and 10S	47-50
Increasing Enzyme Activity through Directed Evolution	51
NMR Spectra	52-74
References	75

Materials:

Unless otherwise noted, all reagents were obtained from commercial suppliers and used without further purification. $[Ir(cod)Cl]_2$ and $[Ru(p\text{-}cymene)Cl_2]_2$ were purchased from Strem. Deuterated solvents were obtained from Cambridge Isotope labs. Silicycle silica gel plates (250 mm, 60 F254) were used for analytical TLC, and preparative chromatography was performed using SiliCycle SiliaFlash silica gel (230-400 mesh). Oligonucleotides were purchased from Integrated DNA Technologies (San Diego, CA), and the sequences of the primers used in this study are reported below.

Plasmids pET-28a/RebF and pET-28a/RebH in BL-21 DE3 E. coli were provided by the Walsh group of Harvard Medical School, Boston, MA.[1] The pLIC-MBP plasmid was provided by the Bottomley group of Monash University, Clayton, Australia.[2] The pGro7 plasmid encoding the groES and groEL chaperone set was purchased from Takara (Otsu, Shiga, Japan). BL21(DE3) E. coli cells were purchased from Invitrogen (Carlsbad, CA). NdeI and HindIII restriction enzymes, T4 DNA polymerase, and Phusion HF polymerase were purchased from New England Biolabs (Ipswitch, MA). Luria broth (LB) and Terrific broth (TB) media were purchased from Research Products International (Mt. Prospect, IL). Qiagen Miniprep Kits were purchased from QIAGEN Inc. (Valencia, CA) and used according to the manufacturer's instructions. All genes were confirmed by sequencing at the University of Chicago Comprehensive Cancer Center DNA Sequencing & Genotyping Facility (900 E. 57th Street, Room 1230H, Chicago, IL 60637). Electroporation was carried out on a Bio-Rad MicroPulser using method Ec2. Ni-nitrilotriacetic acid (Ni-NTA) resin and Pierce® BCA Protein Assay Kits were purchased from Fisher Scientific International, Inc. (Hampton, NH), and the manufacturer's instructions were followed when using both products. Amicon® 30 kD spin filters for centrifugal concentration were purchased from EMD Millipore (Billerica, MA) and used at 4,000 g at 4 °C. The glucose dehydrogenase (GDH-105), FAD, and NAD were purchased from Codexis (Redwood City, CA). Biotage reverse phase columns (SNAP-KP-C18-HS) were purchased from Biotage.

Complete analytical data have been reported in the literature for: *N,N-bis(phenylmethyl)-1H-Indole-3-ethanamine* (**1a**)[3], *7-chloro-tryptamine* (**3**)[4], *6-chloro-tryptamine* (**5**)[5], *5-chloro-tryptamine* (**6**)[6], and *2-oxytryptamine* (**13**)[7].

General Procedures:

Standard molecular cloning procedures were followed.[8] Reactions were analyzed using an Agilent Technologies 1200 UHPLC or Agilent Technologies 6130 LC-MS. Reverse phase preparative chromatography was carried out using a Biotage Isolera One or Agilent Technologies 1100 HPLC. ^{1}H and ^{13}C NMR spectra were recorded at 500 MHz and 126 MHz, respectively, on a Bruker DMX-500 or DRX-500 spectrometer, and chemical shifts are reported relative to residual solvent peaks.[9] All mass spectra were collected at the University of Chicago Mass Spectrometry Service Center. High-resolution mass spectra were obtained on an Agilent Technologies 6224 TOF LC-MS. High-throughput screening was performed using a Bruker Ultraflextreme MALDI-TOF-TOF with a Bruker MTP384 steel massive target plate. RebH protein concentrations were determined by A_{280} measurements taken on a Tecan Infinite M200 pro microplate reader. A Microlab® Nimbus liquid handling robot was used in MALDI target plate preparation, and library expression and screening. Library colonies were picked using a Norgen Systems colony-picking robot.

Error-prone library construction and expression[10]*:* Error-prone PCR was used to construct libraries for rounds 1, 2, 4 and 6. Forward and reverse primers used were 5’ - TTAATACATATGTCCGGCAAGATTGACAAGATCCTC - 3’ and 5’ – TATTAAAAGCTTTCAGCGGCCGTGCTGTTGCCTCAG – 3’, respectively. The PCR conditions were as follows: 1 ng/µl parent template, 10x Taq buffer, 0.2 mM dNTPs each, 0.2 µM forward primer, 0.2 µM reverse primer, 0.025 U/µl Taq polymerase, and 100 µM $MnCl_2$. PCR was performed in a volume of 50 µL with the following procedure: 95 °C 30 s, (95 °C 30 s, 55 °C 30 s, 72 °C 90 s) for 20 cycles, 72 °C 10 min. The resulting RebH insert was gel purified and digested with the restriction enzymes HindIII (0.33 U/µl) and NdeI (0.33 U/µl) in 10x Cutsmart buffer in a final reaction volume of 60 µl. The digestion was conducted at 37 °C for 12-16 hours, after which it was gel purified. This insert was ligated into digested pET-28a (insert:plasmid ratio of 7.5:1) using T4 DNA ligase. Ligations were conducted for 20 hours at 16 °C. Ligations were cleaned with Zymo DNA Cleaning and Concentrating kits and were transformed by electroporation into *E. coli* containing a plasmid encoding the chaperone pGro7. Library colonies were picked using an automated colony picker (Norgren Systems) and arrayed into 96-deep-well plates (1 mL) containing 300 µL LB with 50 µg/mL kanamycin and 20 µg/mL

chloramphenicol. In each 96-well plate, 6 wells were left blank as a control for contamination, and 6 wells were parent cultures. Cells were grown overnight at 37 °C, 250 rpm, and 50-100 µL of overnight culture were used to inoculate 1 mL TB (with 50 µg/mL kanamycin and 20 µg/mL chloramphenicol) in 96-deep-well plates (2 mL) using a liquid handling robot. Following growth at 37 °C, 250 rpm, to an OD_{600} = 0.8-1, enzyme expression was induced with IPTG and arabinose (added using a multichannel pipette) to final concentrations of 10 µM and 0.2 mg/mL, respectively. Protein expression continued for ~20 h at 30 °C, 250 rpm, after which cells were pelleted by centrifugation, the supernatants were discarded, and cell pellets were stored at -20 °C until use.

Targeted library construction and expression: A targeted library approach was used to construct libraries for rounds 7 and 8. Mutations were introduced via overlap extension technique.[11] Primers are listed in the Primer Table. The fragment PCR conditions were as follows: 1 ng/µl parent template, 5x Phusion GC buffer, 0.2 mM dNTPs each, 0.5 µM forward primer, 0.5 µM reverse primer, 0.02 U/µl Phusion polymerase, and 5% v/v DMSO. Fragments were gel purified. The assembly PCR conditions were as follows: 1:1 ratio of fragments, 5x Phusion GC buffer, 0.2 mM dNTPs each, 0.5 µM forward primer, 0.5 µM reverse primer, 0.02 U/µl Phusion polymerase, and 5% v/v DMSO. Fragment and assembly PCR were performed in a volume of 50 µL with the following procedure: 98 °C 30 s, (98 °C 20 s, 55 °C 30 s, 72 °C 90 s) for 28 cycles, 72 °C 10 min. It is important to note DMSO addition was found to enhance specificity and yield of PCR products.

The resulting RebH insert was gel purified and digested with the restriction enzymes HindIII (0.33 U/µl) and NdeI (0.33 U/µl) in 10x Cutsmart buffer in a final reaction volume of 60 µl. The digestion was conducted at 37 °C for 12-16 hours, after which it was gel purified. This insert was ligated into digested pET-28a (insert:plasmid ratio of 7.5:1) using T4 DNA ligase. Ligations were conducted for 20 hours at 16 °C. Ligations were cleaned with Zymo DNA Cleaning and Concentrating kits and were transformed by electroporation into *E. coli* containing a plasmid encoding the chaperone pGro7. Library colonies were picked using an automated colony picker (Norgren Systems) and arrayed in 1-ml 96-well plates containing 300 µL LB with 50 µg/mL kanamycin and 20 µg/mL chloramphenicol. For libraries in which only a single

residue was randomized, a sufficient number of colonies were picked to ensure 95% library coverage[12] (100-150 colonies). For the library in which residues 111-113 were randomized with NDT codons, 1,000 colonies were picked, which corresponds to 44% library coverage.[12] In each 96-well plate, 6 wells were left blank as a control for contamination, and 6 wells were parent cultures. Cells were grown overnight at 37 °C, 250 rpm, and 50-100 µL of overnight culture were used to inoculate 1 mL TB (with 50 µg/mL kanamycin and 20 µg/mL chloramphenicol) in 2-mL 96-well plates using a liquid handling robot. Following growth at 37 °C, 250 rpm, to an OD_{600} = 0.8-1, enzyme expression was induced with IPTG and arabinose (added using a multichannel pipette) to final concentrations of 10 µM and 0.2 mg/mL, respectively. Protein expression continued for ~20 h at 30 °C, 250 rpm, after which cells were pelleted by centrifugation, the supernantants were discarded, and cell pellets were stored at -20 °C until use.

Library Lysis and Screening[10]*:* Cell pellets in 2-mL 96-well plates were thawed and suspended in 300 µL HEPES buffer (25 mM, pH 7.4). Cells were pelleted by centrifugation and the supernatant was discarded to wash away residual culture media. Cell pellets were then suspended in 100 µL HEPES buffer (25 mM, pH 7.4) containing 0.75 mg/mL lysozyme. After incubation at 37 °C, (250 rpm, for 30 min.) cells were flash frozen in liquid nitrogen and thawed in a 37 °C water bath. DNaseI (10 µL of 1 mg/mL, 25 mM HEPES buffer, pH 7.4) was added, and the cells were incubated at 37 °C, 250 rpm, for 15 min. After centrifugation, 50 µL of supernatant containing cell lysates was transferred to a microtiter plate for screening using a liquid handling robot.

Halogenation reaction set-up: Similar to what has been described previously for halogenation reactions,[4,10] MBP-RebF (0.0017 equiv., 2.5 µM final concentration) and glucose dehydrogenase (9 U/mL final concentration) were added as solutions (25 mM HEPES, pH 7.4) to the RebH lysate. A solution containing deutero-tryptamine (1 equiv., 1.5 mM final concentration), NAD (0.067 equiv., 100 µM final concentration), FAD (0.067 equiv., 100 µM final concentration), NaCl (66.7 equiv., 100 mM final concentration), and glucose (13.3 equiv., 20 mM final concentration) was added via multichannel pipette to simultaneously initiate the reactions (final reaction volume of 75 µL). Probe **1** (7-deutero-tryptamine) was used as substrate in rounds 1, 2, 4 and 6. Probe **2** (5-deutero-tryptamine) was used as substrate in rounds 7 and 8 to directly

determine 5-halogenation hits, and to narrow the pool of potential 6-halogenation hits. The smaller size of the NNK libraries allowed for the use of UPLC Method 2 as a secondary screen for variants found by MALDI MS with conversion and good selectivity for 6/7-chlorotryptamine. If larger libraries were needed, the 6-deuterotryptamine probe could have been synthesized, but for this study, it was not required. The microtiter plates were sealed (ALPS 3000, Thermo Scientific) and incubated at 37 °C (rounds 1, 2, and 4) or 25 °C (rounds 6,7, and 8) in a vertical incubator at 150 rpm for 12-16 hours. The next morning, reactions were quenched with 75 μL of methanol. Ten microliters of 75 mM HCl was added to each reaction using a multichannel pipette, which was required for spotting mixtures onto a MALDI target without re-dissolving the pre-spotted matrix, but was done regardless of how the reactions were analyzed. Precipitated protein was then pelleted by centrifugation, and the supernatant was transferred to a 96-well 0.45 μm filter plate using a liquid handling robot. The filter plate was loaded on top of a new 96-well microtiter plate and centrifuged at 2,453 g for 10 minutes, or until all supernatant had passed through the filter. A liquid handling robot was then used to transfer filtered reaction mixtures onto a MALDI target plate (detailed MALDI target preparation shown below) or a microtiter plate that could be sealed (ALPS 3000, Thermo Scientific) for subsequent HPLC analysis if necessary.

*As a side note, both MBP-RebF and RebH variants can be lyophilized into powder without any loss of activity. Although lyophilized protein was not used specifically in these studies, it has been used successfully for other purposes with several variants described in this study. When using lyophilized halogenase or reductase, the powder is simply dissolved in water and added to reactions.

MALDI target preparation and screening: A 384-well MALDI target plate was spotted with 2 μL of a solution of α-cyano-4-hydroxycinnamic acid by a liquid handling robot. The matrix solution contained 7.5 mg/mL of α-cyano-4-hydroxycinnamic acid in 1:1 THF:H_2O. This was dried in a vacuum oven for 15 minutes. Once dry, 2 μL of the filtered bioconversions were spotted onto the MALDI target plate by a liquid handling robot. This was dried in a vacuum oven for 15-30 minutes. The plate was loaded into a Bruker Ultraflextreme MALDI-TOF-TOF and an automated method was developed. Spectra were generated with the reflectron positive (RP) mode. The detector range was set at 160-200 Da. Final mass spectra were produced by

averaging 500 raster shots taken at 50 random positions within each spot, which amounted to 25-30 seconds per spot. Targets were shot using the AutoXecute tool of the Flex Control acquisition software.

MALDI MS data analysis and HPLC screening: MALDI MS peaks were analyzed in Flex Analysis software. All peaks within the detector range besides those corresponding to deutero-tryptamine, chlorotryptamine, and deutero-chlorotryptamine (m/z = 162, 195, and 196) were set as background. Data from the spectra for these three peaks were then exported into Excel. An Excel macro was developed to insert the value "0" for spectra that did not contain peaks 195 and/or 196. From this list, the data could be easily arrayed into conversions and selectivities in 96-well-plate format in Excel. Hits were identified as variants that showed higher conversion relative to parent or higher selectivity relative to parent. When using Probe **1**, the ratios 196/(162+196+195) (for tryptamine conversion) and 196/(196+195) (for selectivity) were calculated. When using Probe **2**, 195/(162+196+195) and 195/(196+195) were calculated. These ratios were compared to those of the parent reactions, and the highest hits were re-screened by UPLC. Hits confirmed by UPLC were sequenced and verified by enzyme purification and re-analysis. For rounds 7 and 8, 5-chlorination hits were directly identified by MALDI MS using 195/(162+196+195) and 195/(196+195). Variants with high values for 196/(162+196+195) and 196/(196+195) were re-screened by UPLC Method 2 (see below) to distinguish 6- from 7-chlorination.

UHPLC/LC-MS methods:

UHPLC/LC-MS Method 1: Agilent Eclipse Plus C18 4.6 x 150 mm column, 3.5 μM particle size; solvent A = H_2O/0.1% TFA, solvent B = CH_3CN; 0-10 min, B = 15%; 10-17 min, B = 15-22%; 17-20 min, B = 22-30%; 20-21 min, B = 30%. Absorbance at 280 nm was measured.

UHPLC/LC-MS Method 2: Agilent Eclipse Plus C18 4.6 x 50 mm column, 3.5 μM particle size; solvent A = H_2O/0.1% TFA, solvent B = CH_3CN; 0-4 min, B = 15%; 4-4.5 min, B = 15-20%; 4.5-6.5 min, B = 20-30%; 6.5-7 min, B = 100%. Absorbance at 280 nm was measured.

Expression and Purification of MBP-RebF and RebH: For preparative bioconversions and kinetic studies, large-scale cultures (750 mL) of MBP-RebF and RebH variants were grown, expressed and purified as previously reported[4]. MBP-RebF concentrations were measured using the Pierce BCA Protein Assay Kit. RebH concentrations were determined using A_{280} and extinction coefficients calculated based on amino acid composition (Protein Calculator v3.3, http://www.scripps.edu/~cdputnam/protcalc.html).

Smaller cultures (50 mL) of RebH variants were grown to compare conversion and selectivity between variants. Primary culture (500 µL) was used to inoculate 50 mL TB (with 50 µg/mL kanamycin and 20 µg/mL chloramphenicol). Following growth at 37 °C, 250 rpm, until OD_{600} = 0.6-0.8, enzyme expression was induced with IPTG and arabinose to final concentrations of 100 µM and 2 mg/mL, respectively. Protein expression continued for ~20 h at 30 °C, 250 rpm, after which cultures were harvested by centrifugation. Cell pellets were suspended in 10 mL 25 mM HEPES (pH 7.4) in 50 mL conical tubes, and lysed by sonication while kept on ice.

Sonication Conditions:

50 mL cultures: sonication was performed on a Qsonica S-4000 Sonicator with a 0.5" horn using the following procedure: 8 x 30 s with 45 s rests, 20% duty cycle delivering 40-50 W. To keep the sample from over-heating, the conical tube was submerged in a circulating ice-water bath.

750 mL cultures: sonication was performed on a Qsonica S-4000 Sonicator with a 0.5" horn using the following procedure: 5 x 1 min with 1 min rests, 20% duty cycle delivering 40-50 W. To keep the sample from over-heating, the conical tube was submerged was submerged in a circulating ice-water bath.

After sonication, cell debris was pelleted by centrifugation and the clarified lysate was passed over a Ni-NTA affinity chromatography column. RebH was eluted from the Ni-NTA with 250 mM imidazole. RebH containing fractions were pooled and exchanged into a buffer of 25 mM HEPES (pH 7.4), 10 % glycerol. RebH concentrations were determined using A_{280} and extinction coefficients were again calculated based on amino acid composition using Protein Calculator v3.3. Protein stocks were then stored at -20 °C until use.

Preparative tryptamine bioconversions: Preparative bioconversions were conducted similarly for all enzymes. A solution of 1, 2 or 10 mg substrate (1 equiv., 0.5 mM final concentration) in 0.5% v/v isopropanol was added to a reaction vessel (crystallization dish - 100 x 50 mm, or 50 mL Erlenmeyer flask). Solutions in HEPES buffer (25 mM, pH 7.4) of NAD (0.2 equiv., 100 μM final concentration), FAD (0.2 equiv., 100 μM final concentration), NaCl (20 or 200 equiv., 10 or 100 mM final concentration), and a glucose dehydrogenase (9 U/mL final concentration GDH) were added. Solutions in HEPES/glycerol buffer (25 mM, pH 7.5, 10% glycerol v/v) of RebH (0.02-0.1 equiv., 10-50 μM final concentration) and MBP-RebF (0.005 equiv., 2.5 μM final concentration) were added. The bioconversion was diluted with HEPES buffer (25 mM, pH 7.4) to the appropriate reaction volume and an aqueous solution of glucose (40 equiv., 20 mM final concentration) was added to initiate the cofactor regeneration cycle. The dish was covered with perforated aluminum foil and agitated in a vertical incubator at 90 rpm at 10-25 °C.

Reactions were monitored by HPLC Method 1 as described above and were quenched with aqueous HCl (5.0 M, until pH ~1-2) upon completion. NaCl was added to saturation. Precipitated protein was filtered out through a pad of Celite and was washed with water. The filtrate was basified (pH ~9-12) and extracted into CH_2Cl_2. The crude material was purified by reverse phase chromatography, on a Biotage or a preparative HPLC (Agilient 1100).

Reported conversions are the unadjusted final conversions of substrate to chlorinated products. Selectivities of preparative reactions were calculated by comparing analogous protons in the aryl region of ^{1}H NMR spectra.

Table of Primers.

Targeted Amino Acids Residues	Forward Mutagenic Primer (5'-3')	Reverse Mutagenic Primer (5'-3')	Template
pET28-RebH Primers	TTAATACATATGTCCGGCAAGATTGACAAGATC CTC	TATTAAAAGCTTTCAGCGGCCGTGCTGTTGCCTCA G	All RebH variants
N470S	CTTCTGGAACAACAGCAGCTACTACTGC	GCAGTAGTAGCTGCTGTTGTTCCAGAAG	wtRebH
R509Q	TCAAGGACCAGCAGCGGAA	TTCCGCTGCTGGTCCTTGA	2RF
Y455W	AGCTCTACTGGGGCAACTTC	GAAGTTGCCCCAGTAGAGCT	2RFQ
F111L	CTACCACCCCTTAGGTCTGC	GCAGACCTAAGGGGTGGTAG	4P
S130L	GGTTCGACCGTTTGTACCGGGGGAAG	CTTCCCCCGGTACAAACGGTCGAACC	4PL
N166S	CCAAGGTGACGAGCTACGCGTGGCAC	GTGCCACGCGTAGCTCGTCACCTTGG	4PL
L111S	ACCACCCCTCAGGTCTGCT	AGCAGACCTGAGGGGTGGT	5LS
I52T	AGGCCACGACTCCCAATCT	AGATTGGGAGTCGTGGCCT	wtRebH, 5LS
F465L	TTCCGCAACCTCTGGAACA	TGTTCCAGAGGTTGCGGAA	wtRebH, 5LS
NDT: F111, G112, L113	TTCTACCACCCC**NDTNDTNDT**CTCAAGTACCAC	GTGGTACTTGAG**AHNAHNAHN**GGGGTGGTAGAA	6S
NNK: I52	CGAGGCCACG**NNK**CCCAATCTGCA	TGCAGATTGGG**MNN**CGTGGCCTCG	6TL, 7W
NNK: F465	GAGTTCCGCAAC**NNK**TGGAACAACAGC	GCTGTTGTTCCA**MNN**GTTGCGGAACTC	6TL, 7H, 7V, 7M
NDT: W466	TTCCGCAACCTC**NDT**AACAACAGCAGC	GCTGCTGTTGTT**AHN**GAGGTTGCGGAA	6TL
NDT: P53	GAGGCCACGACT**NDT**AATCTGCAGACG	CGTCTGCAGATTA**AHN**AGTCGTGGCCTC	6TL
NDT: Y362	TCGACGGGCATC**NDT**TTCGTCTACGCC	GGCGTAGACGAA**AHN**GATGCCCGTCGA	5LS
NNK: 111	TCTACCACCCC**NNK**GGTCTGCTC	GAGCAGACC**MNN**GGGGTGGTAGA	7M
I52M	GAGGCCACGATGCCCAATCTG	CAGATTGGGCATCGTGGCCTC	wtRebH + F465L
I52H	CGAGGCCACGCATCCCAATCTGCA	TGCAGATTGGGATGCGTGGCCTCG	wtRebH, 0S, 1P, 2RFQ, 3W, 4PL
F465C	GAGTTCCGCAACTGTTGGAACAACAGC	GCTGTTGTTCCAACAGTTGCGGAACTC	wtRebH, 0S, 1P, 2RFQ, 3W, 4PL
L111F	CTACCACCCCTTCGGTCTGCTC	GAGCAGACCGAAGGGGTGGTAG	8C

Detailed Experimental Procedures:

Kinetic isotope effect of deuterium labeling: In order to affirm deprotonation of the arene occurs after the rate-limiting step, and thus has little to no contribution to the observed selectivity on the deuterated tryptamine probes, initial rates of L-tryptophan and d_5-L-tryptophan chlorination were compared. RebH variant 0S (0.002 equiv., 1 μM final concentration), MBP-RebF (0.005 equiv., 2.5 μM final concentration), glucose dehydrogenase (9 U/mL final concentration), and FAD (0.2 equiv., 100 μM final concentration) were added as solutions (25 mM HEPES, pH 7.4) to an Eppendorf tube. A solution containing L-tryptophan or d_5-L-tryptophan (1 equiv., 0.5 mM final concentration), NAD (0.2 equiv., 100 μM final concentration), NaCl (200 equiv., 100 mM final concentration), phenol (internal standard, 1 equiv., 0.5 mM final concentration) and glucose (40 equiv., 20 mM final concentration) was added to this tube to initiate reaction (1200 μL final reaction volume for each substrate). This reaction was then split into 75 μL aliquots in a 96-well microtiter plate. The L-tryptophan reaction was initiated and pipetted into the microtiter plate first, followed by *d5*-L-tryptophan. The reactions were mixed at 650 rpm on top of an Eppendorf air bath and were quenched with 1 volume (75 μL) methanol at various time points from 10-45 min. Reactions were conducted in triplicate. The precipitated protein was removed by centrifugation and the reactions were filtered and analyzed by UPLC Method 2 from the General Procedures. Product formation was obtained by fitting the ratio of product to internal standard to a calibration curve prepared from known concentrations of chlorinated tryptophan. The initial rates of L-tryptophan and d_5-L-tryptophan were found to be 2.1076 and 2.0257 μM/min, respectively (Fig. S1). This suggests there is no significant isotope effect for RebH halogenation of H/D tryptophan (k_H/k_D = 1.04). Note: Because of the time difference between the two reaction initiations, the conversions of L-tryptophan in Fig. S1 are slightly higher than *d5*-L-tryptophan. The number of minutes in between time points, however, is identical in both cases, thus the initial rates can be accurately determined from the slopes.

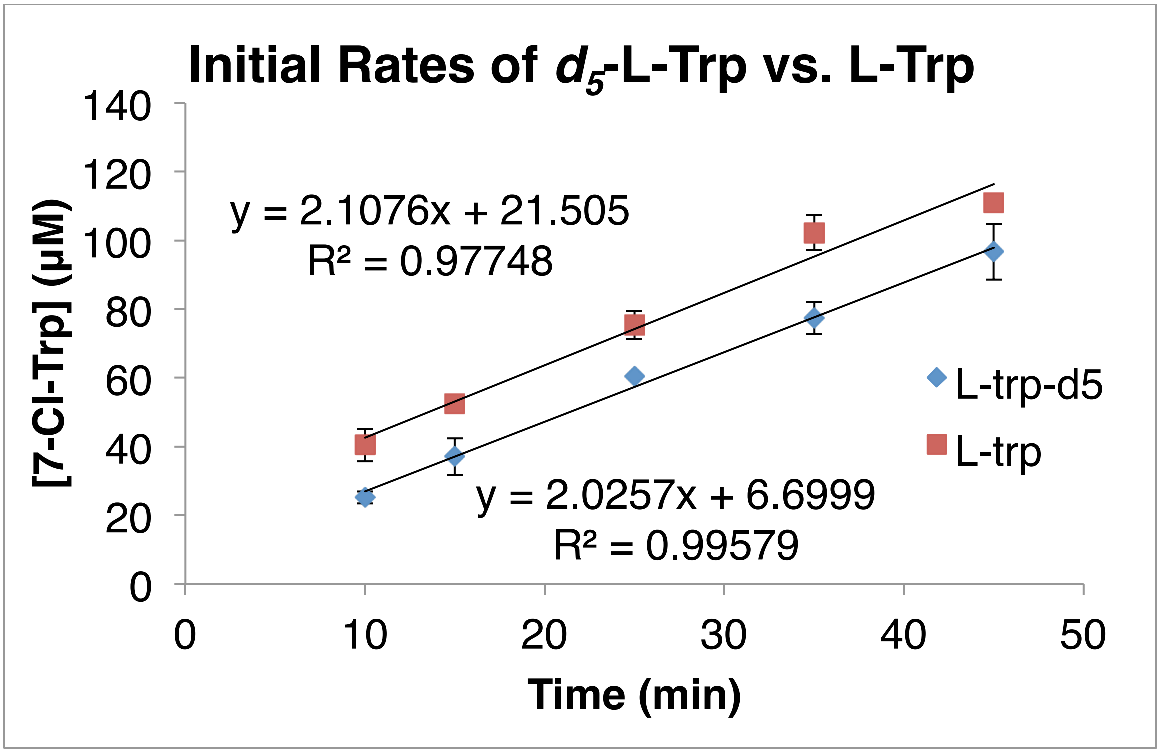

Figure S1: The initial rates of RebH halogenation of d_5-L-tryptophan and L-tryptophan were independently determined. No significant isotope effect was observed ($k_H/k_D = 1.04$).

In a second experiment, d_5-L-tryptophan and L-tryptophan were added in equal amounts (1 equiv., 0.5 mM final concentration each) to reactions using the same method as described above. Reactions were quenched with methanol at times between 9-45 min. The precipitated protein was removed by centrifugation. The reactions were filtered and analyzed by LC-MS using Method 2 described in the General Procedures. Product formation was obtained by fitting the ratio of product to internal standard to a calibration curve prepared from known concentrations of chlorinated tryptophan. The ratio of H/D-chlorotryptophan was determined by finding the relative intensities of m/z = 239 and m/z = 243 in the mass spectrum of the product peak. Again, no significant isotope effect was observed ($k_H/k_D = 0.97$, Fig. S2).

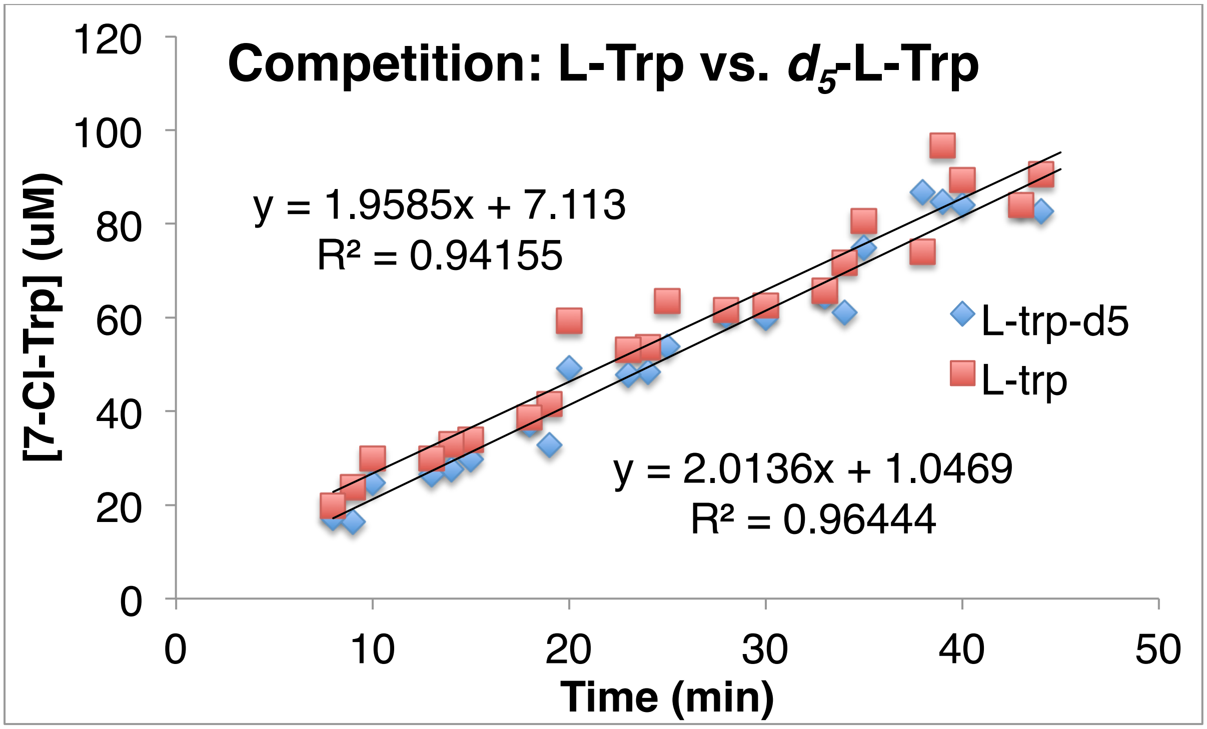

Figure S2: In a competition experiment, the initial rates of RebH halogenation of d_5-L-tryptophan and L-tryptophan were determined. No significant isotope effect was observed ($k_H/k_D = 0.97$).

Using MALDI MS to analyze RebH bioconversions: To determine whether reaction conversion could be measured by MALDI MS, a time course of wtRebH chlorination of tryptamine was conducted and analyzed by UPLC and MALDI MS. wtRebH (0.05 equiv., 25 µM final concentration), MBP-RebF (0.005 equiv., 2.5 µM final concentration), and glucose dehydrogenase (9 U/mL final concentration) were added as solutions (25 mM HEPES, pH 7.4) to an Eppendorf tube. A solution containing tryptamine (1 equiv., 0.5 mM final concentration), FAD (0.2 equiv., 100 µM final concentration), NAD (0.2 equiv., 100 µM final concentration), NaCl (20 equiv., 10 mM final concentration), and glucose (40 equiv., 20 mM final concentration) was added to this tube to initiate reaction (600 µL final reaction volume). This reaction was then split into 75 µL aliquots in Eppendorf tubes. These were agitated at 650 rpm in an air bath at 25 °C. Reactions were quenched with 1 volume (75 µL) methanol at various time points from 30-210 min. Precipitated protein was removed by centrifugation, and the reactions were filtered according to the method found in the General Procedures. Ten microliters

of 75 mM HCl was added to each reaction to slightly acidify it, which allowed spotting onto a MALDI target without re-dissolving the pre-spotted matrix.

A MALDI target was spotted by hand with matrix, followed by the acidified bioconversions using the method described in the General Procedures. Spots were analyzed using the MALDI MS automatic method outlined in the General Procedures. Percent conversion was calculated by taking the intensity of the m/z peak for chlorotryptamine (195) divided by the added intensities of the chlorotryptamine peak (195) and tryptamine (161). After the MALDI target plate had been spotted with reaction mixtures, these same reactions were analyzed by UPLC using Method 1 described in the General Procedures. Percent conversion was calculated by taking the area of the product peak divided by the area of the combined product and starting material peaks. This data was plotted below. It was found that, while the MALDI MS response was not linear, it could be used to determine relative conversions (Fig. S3). This allows MALDI MS analysis to function as an initial screen of libraries. A secondary HPLC/LC-MS analysis of conversion and selectivity could be performed for hits found using this MALDI MS screen.

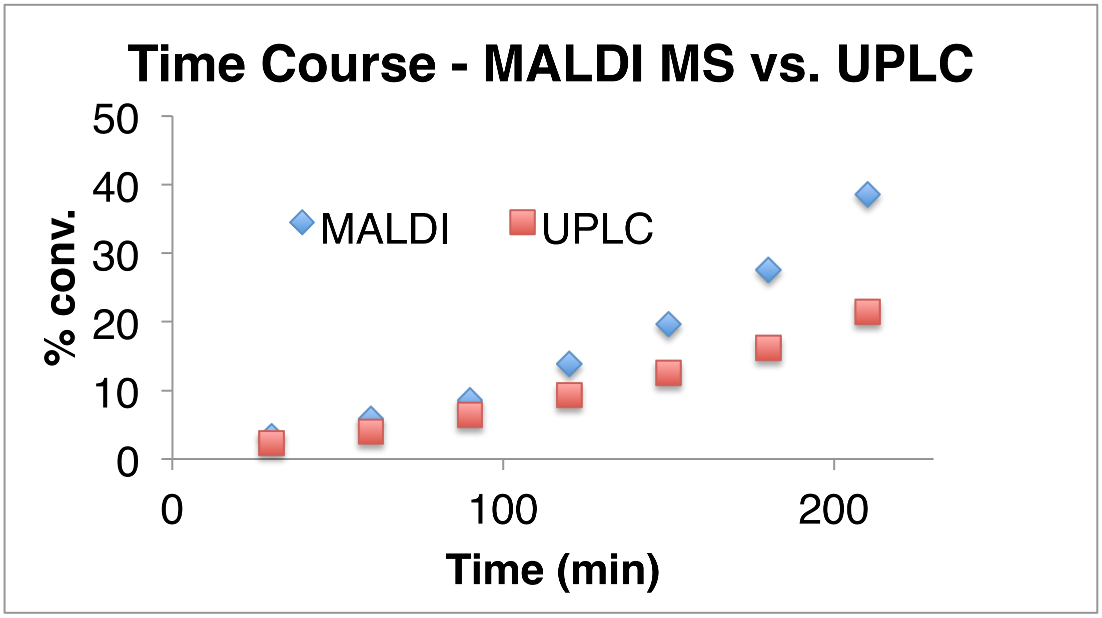

Figure S3: Although a linear response is not observed, MALDI-MS can be used to determine relative conversions between reactions.

Detection of different H/D-tryptamine ratios: Once deuterated Probe **1** had been synthesized, the reliability of MALDI MS to distinguish tryptamine from Probe **1** was explored. Solutions

containing tryptamine (0.5 mM final concentration), FAD (100 µM final concentration), NAD (100 µM final concentration), NaCl (10 mM final concentration), and glucose (20 mM final concentration) were made in 25 mM HEPES, pH 7.4. Each solution contained a different amount of Probe **1** (0.01-0.5 mM final concentration). These solutions (75 µL each) were acidified (10 uL of 75 mM HCl) and 1 volume of methanol was added to each to mimic bioconversion preparation. The acidified solutions were spotted by hand on a MALDI target plate and shot with the automatic method described in the General Procedures. The ratio of the intensity of the m/z peak corresponding to Probe **1** (162) and the intensity of the m/z peak corresponding to tryptamine (161) was calculated and plotted against the known concentration of Probe **1** (Fig. S4). This plot demonstrates that the MALDI MS screen can be used to determine the relative ratio of Probe **1**:tryptamine.

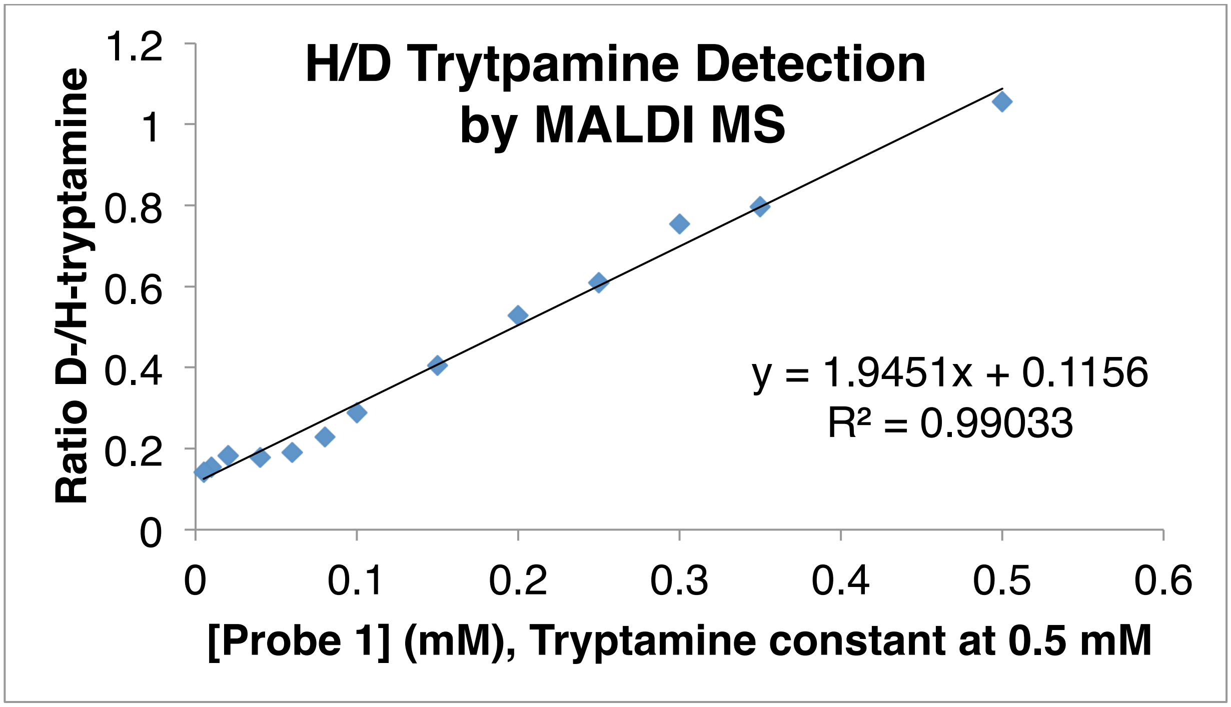

Figure S4: When different ratios of D-/H-tryptamine are analyzed by MALDI MS, a linear response is observed, suggesting this screen can be used to identify differences in D-/H-chlorotryptamines.

Synthesis and characterization of deuterated probes

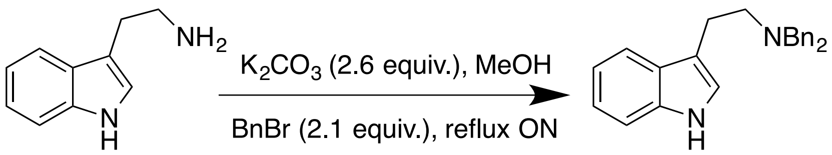

*N,N-bis(phenylmethyl)-1H-Indole-3-ethanamine (**1a**):* Tryptamine (2 g, 12.48 mmol, 1 equiv.) and K_2CO_3 (4.5 g, 32.56 mmol, 2.6 equiv.) were added to a round bottom flask under nitrogen. Dry methanol (60 mL) was added, followed by benzyl bromide (3.12 mL, 26.24 mmol, 2.1 equiv.). The reaction mixture was heated to reflux for 12 hours. The crude reaction mixture was passed through filter paper and the filtrate was concentrated onto silica gel. Purification by flash chromatography (SiO_2, 20% ethyl acetate/hexanes) afforded the known compound[3] **1a** in 79% yield (3.36 g, 9.88 mmol). ^1H NMR (500 MHz; $CDCl_3$): δ 7.88 (s, 1H), 7.47-7.42 (m, 5H), 7.38-7.35 (m, 5H), 7.29 (t, J = 7.3, 2H), 7.21 (t, J = 7.6, 1H), 7.09 (td, J = 7.5, 0.9, 1H), 6.94 (d, J = 2.1, 1H), 3.76 (s, 4H), 3.04 (t, J = 7.9, 2H), 2.87 (t, J = 7.8, 2H). HRMS (ESI-TOF) calcd for $C_{24}H_{24}N_2$ $[M + H]^+$: 341.2012, found: 341.0735.

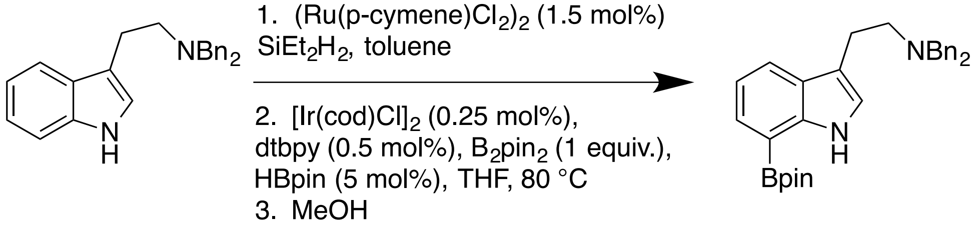

*7-(4,4,5,5-tetramethyl-1,3,2-dioxaborolan-2-yl)-N,N-bis(phenylmethyl)-1H-Indole-3-ethanamine (**1b**):* The procedure for this reaction was adapted from a previous report.[13] **1a** (1.8651 g, 5.49 mmol, 1 equiv.), $[Ru(p\text{-cymene})Cl_2]_2$ (50 mg, 0.0823 mmol, 0.015 equiv.), diethylsilane (1.07 mL, 8.235 mmol, 1.5 equiv.) and toluene (2.75 mL) were added to a dry Teflon-sealed Schlenk flask inside a glovebox. The reaction mixture was stirred at room temperature until full conversion to the *N*-silyltryptamine was observed by ^1H NMR (~10 hours). The solvent was removed under vacuum. $[Ir(cod)Cl]_2$ (9.2 mg, 0.0137 mmol, 0.0025 equiv.), dtbpy (7.9 mg, 0.0275 mmol, 0.005 equiv.), B_2pin_2 (1.394 g, 5.49 mmol, 1 equiv.), HBpin (39.9 μL, 0.275 mmol, 0.05 equiv.) and THF (5.49 mL) were added to the flask. The reaction was removed from the glovebox and stirred at 80 °C until full conversion was observed by ^1H NMR. After the reaction was cooled, MeOH (~5.5 mL) was added. This was stirred until complete desilylation

was observed by ^{1}H NMR. The reaction mixture was concentrated onto silica gel. Purification by flash chromatography (SiO_2, 8% ethyl acetate/hexanes) afforded **1b** in 68% yield (1.733 g, 3.733 mmol). ^{1}H NMR (500 MHz; $CDCl_3$): δ 8.95 (s, 1H), 7.60 (d, J = 7.0 Hz, 1H), 7.48 (d, J = 7.8 Hz, 1H), 7.39 (d, J = 7.4 Hz, 4H), 7.30 (t, J = 7.5 Hz, 4H), 7.23 (t, J = 7.2 Hz, 2H), 7.03 (t, J = 7.5 Hz, 1H), 6.97 (s, 1H), 3.69 (s, 4H), 2.99 (t, J = 7.8 Hz, 2H), 2.80 (t, J = 7.8 Hz, 2H), 1.38 (s, 12H). ^{13}C NMR (126 MHz; $CDCl_3$): δ 141.61, 140.14, 129.34, 129.02, 128.37, 126.97, 126.69, 122.67, 121.57, 118.78, 114.25, 83.92, 58.59, 54.45, 25.20, 23.42. ^{11}B NMR (160 MHz; $CDCl_3$): δ 31.33. HRMS (ESI-TOF) calcd for $C_{30}H_{35}N_2O_2B$ [M + H]+: 467.2870, found: 467.1362.

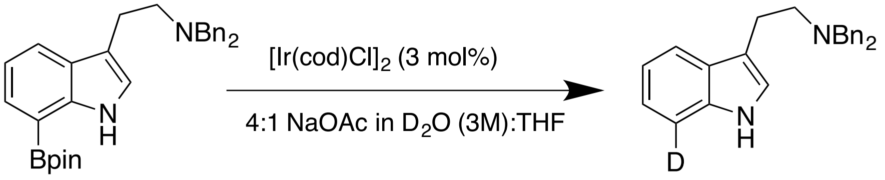

7-deutero- N,N-bis(phenylmethyl)-1H-Indole-3-ethanamine (1c): In a glovebox, **1b** (1 g, 2.15 mmol, 1 equiv.) and $[Ir(cod)Cl]_2$ (44 mg, 0.0655 mmol, 0.03 equiv.) were added to a dry round bottom flask. The flask was taken out of the glovebox and placed under nitrogen. Dry THF (9.5 mL) and D_2O/NaOAc (38.8 mL, 3M) were added. The reaction mixture was stirred at room temperature until full conversion of the starting material was observed by TLC (~48 hours). Purification by flash chromatography (SiO_2, 20% ethyl acetate/hexanes) afforded **1c** in 90% yield (659 mg, 1.93 mmol). ^{1}H NMR (500 MHz; $CDCl_3$): δ 7.88 (s, 1H), 7.43-7.36 (m, 5H), 7.30 (t, J = 7.2, 4H), 7.23 (t, J = 7.1, 2H), 7.16 (d, J = 7.0, 1H), 7.06-7.01 (m, 1H), 6.92 (d, J = 1.0, 1H), 3.70 (s, 4H), 2.99 (t, J = 7.7, 2H), 2.82 (t, J = 7.7, 2H). ^{13}C NMR (126 MHz; $CDCl_3$): δ 139.90, 136.22, 128.96, 128.29, 127.64, 126.96, 121.79, 121.53, 119.17, 118.91, 114.52, 110.78 (t), 58.40, 53.94, 23.10. HRMS (ESI-TOF) calcd for $C_{24}H_{23}DN_2$ [M + H]+: 342.2075, found: 342.0788.

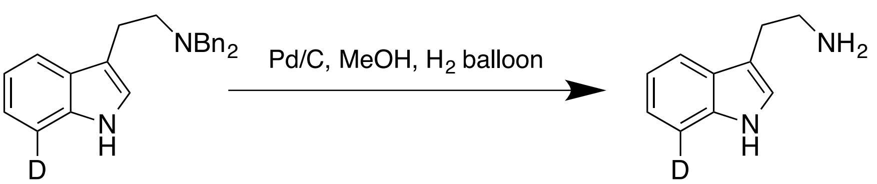

7-deutero-1H-Indole-3-ethanamine (Probe 1): **1c** (250 mg, 0.733 mmol, 1 equiv.) and Pd/C (150 mg, 10 wt. % loading on activated carbon) were added to a round bottom flask under nitrogen. Dry MeOH (25 mL) was added as solvent. The flask was purged with hydrogen for 15

minutes, after which the reaction was kept under 1 atm of hydrogen by balloon. The reaction was stirred at room temperature and monitored by HPLC until full conversion to the debenzylated product was observed. The reaction mixture was filtered through Celite and subsequently concentrated onto Celite. The Celite was packed into a Biotage samplet, which was then loaded into a reverse phase column (Biotage SNAP-KP- C18-HS). The crude material was purified by reverse phase chromatography (gradient from water 0.1% TFA to 15% CH_3CN/water 0.1%TFA). When concentrated by rotovap, the deuterium label is partially lost due to proton exchange. Instead, 7-deutero-tryptamine-containing fractions were pooled, basified and extracted into DCM. The DCM was concentrated by rotovap to afford Probe **1** in 68% yield (80.2 mg, 0.499 mmol). ^{1}H NMR (500 MHz; MeOD): δ 7.56 (d, J = 7.8, 1H), 7.16 (s, 1H), 7.12 (d, J = 6.9, 1H), 7.04 (t, J = 7.5, 1H), 3.22 (t, J = 7.1, 2H), 3.11 (t, J = 7.2, 2H). ^{13}C NMR (126 MHz; MeOD): δ 138.21, 128.75, 123.54, 122.22, 119.56, 119.26, 113.40, 111.99 (t), 43.07, 29.52. HRMS (ESI-TOF) calcd for $C_{10}H_{11}DN_2$ [M + H]+: 162.1136, found: 162.2032.

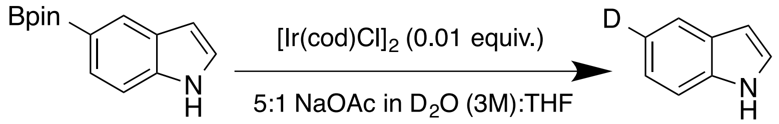

*5-deutero-indole (**2a**):* In a glovebox, 5-(4,4,5,5-tetramethyl-1,3,2-dioxaborolan-2-yl)-1H-indole (5 g, 20.6 mmol, 1 equiv.) and $[Ir(cod)Cl]_2$ (138 mg, 0.206 mmol, 0.01 equiv.) were added to a dry round bottom flask. The flask was taken out of the glovebox and placed under nitrogen. Dry THF (20 mL) and D_2O/NaOAc (100 mL, 3M) were added, and the mixture was stirred at room temperature until full conversion of starting material was observed by TLC (~48 hours). Purification by flash chromatography (SiO_2, 5% ethyl acetate/hexanes) afforded **2a** in 78% yield (1.886 g, 16.0 mmol). ^{1}H NMR (500 MHz; $CDCl_3$): δ 8.01 (s, 1H), 7.74 (s, 1H), 7.41 (d, J = 8.2, 1H), 7.28 (d, J = 8.2, 1H), 7.20 (t, J = 2.8, 1H), 6.63 (m, 1H). ^{13}C NMR (126 MHz; CD_2Cl_2): δ 136.37, 128.41, 124.71, 122.23, 120.90, 119.89 (t), 111.47, 102.81. HRMS (ESI-TOF) calcd for C_8H_6DN [M + H]+: 119.0714, found: 118.9099.
*It should be noted that some amount of deuteration at the C-3 position was occasionally observed at longer reaction times. Because the C-3 position is deprotonated during alkylation with oxalyl chloride, this was not an issue for the synthesis of this tryptamine probe. If

necessary, the 3-deuteration can be removed by reaction of product with 0.01 M HCl at 60 °C for 4 hours.[14]

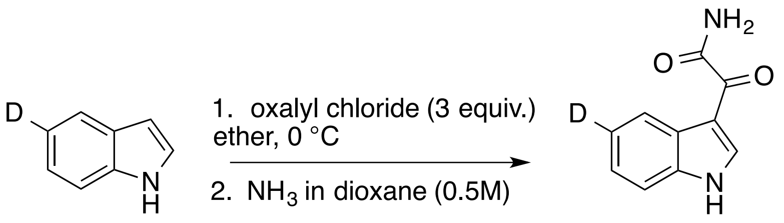

*7-deutero-α-oxo-H-Indole-3-acetamide (**2b**):* The procedure for this reaction was adapted from a previous report.[15] Oxalyl chloride (304 µL, 3.54 mmol, 3 equiv.) was added dropwise to a stirring solution of **2a** (140 mg, 1.19 mmol, 1 equiv.) in dry ether (10.5 mL) under nitrogen at 0 °C. The reaction mixture was then warmed to room temperature and stirred until full conversion of indole was observed by TLC. Stirring was stopped, and the reaction mixture was allowed to settle. The supernatant was removed and discarded. The precipitant was placed back under nitrogen, and ammonia in dioxane (14 mL, 0.5 M) was added. This mixture was stirred at room temperature for 14 hours. $KHCO_3$ was added, and the reaction mixture was filtered. Filter paper was rinsed with hot acetone. The combined filtrates were concentrated onto silica gel. Purification by flash chromatography (SiO_2, 50% ethyl acetate/hexanes) afforded **2b** in 60% yield (136 mg, 0.72 mmol). ^{1}H NMR (500 MHz; DMSO): δ 12.19 (s, 1H), 8.68 (d, J = 3.0, 1H), 8.22 (s, 1H), 8.07 (s, 1H), 7.70 (s, 1H), 7.53 (d, J = 8.1, 1H), 7.26 (d, J = 8.1, 1H). ^{13}C NMR (126 MHz; DMSO): δ 183.43, 166.48, 138.66, 136.77, 126.62, 123.72, 122.65 (t), 121.61, 112.97, 112.59. HRMS (ESI-TOF) calcd for $C_{10}H_7DN_2O_2$ [M + H]+: 190.0721, found: 190.0727.

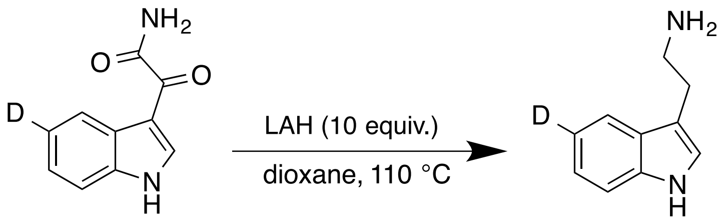

*5-deutero-1H-Indole-3-ethanamine (Probe **2**):* The procedure for this reaction was adapted from a previous report.[15] In a glovebox, lithium aluminum hydride (271.4 mg, 7.2 mmol, 10 equiv.) was added to a solution of **2b** (136 mg, 0.72 mmol, 1 equiv.) in dry dioxane (44 mL). The

reaction was removed from the glovebox and refluxed at 110 °C under nitrogen until full conversion to 5-deutero-tryptamine was observed by HPLC. To quench the excess LAH, H_2O (272 µL) was added slowly, followed by addition of NaOH (272 µL 15% w/v) and H_2O (816 µL). The reaction mixture was stirred for 1 hour, then filtered over Celite through a coarse fritted glass filter and washed with methanol. The filtrate was concentrated onto Celite. The Celite was packed into a Biotage samplet, which was then loaded into a reverse phase column (Biotage SNAP-KP-C18-HS). The crude material was purified by reverse phase chromatography (gradient from water 0.1% TFA to 15% CH_3CN/water 0.1%TFA). When concentrated by rotovap, the deuterium label is partially lost due to proton exchange. Instead, 5-deutero-tryptamine-containing fractions were pooled, basified and extracted into DCM. The DCM was concentrated by rotovap to afford Probe **2** in 40% yield (46.2 mg, 0.288 mmol). ^{1}H NMR (500 MHz; $CDCl_3$): δ 8.58 (s, 1H), 7.63 (s, 1H), 7.35 (d, J = 8.1, 1H), 7.21 (d, J = 8.2, 1H), 7.00 (s, 1H), 3.05 (t, J = 6.6, 2H), 2.93 (t, J = 6.6, 2H). ^{13}C NMR (126 MHz; $CDCl_3$): δ 136.61,127.62, 122.20, 121.92, 118.82, 113.69, 111.28, 42.44, 29.57. HRMS (ESI-TOF) calcd for $C_{10}H_{11}DN_2$ [M + H]+: 162.1136, found: 162.1142.

Detailed Mutagenesis Protocols

Round 0: RebH variants that had been previously evolved for substrate scope and stabiltiy[10] were tested for conversion of tryptamine to chlorotryptamine. One variant, 2T + N470S, showed higher conversion (>2-fold) than wtRebH. Because lower conversion was observed for the mutant 2T than wtRebH, variant 0S (wtRebH + N470S) was created using overlap extension PCR. Primer sequences can be found in the Primer Table. The fragment PCR conditions were as follows: 1 ng/µl parent template, 5x Phusion GC buffer, 0.2 mM dNTPs each, 0.5 µM forward primer, 0.5 µM reverse primer, 0.02 U/µl Phusion polymerase, and 5% v/v DMSO. Fragments were gel purified. The assembly PCR conditions were as follows: 1:1 ratio of fragments, 5x Phusion GC buffer, 0.2 mM dNTPs each, 0.5 µM forward primer, 0.5 µM reverse primer, 0.02 U/µl Phusion polymerase, and 5% v/v DMSO. Fragment and assembly PCR were performed in a volume of 50 µL with the following procedure: 98 °C 30 s, (98 °C 20 s, 55 °C 30 s, 72 °C 90 s) for 28 cycles, 72 °C 10 min. Pure 0S protein was obtained using the expression and purification described above in the General Procedures. RebH variants (0.02 equiv., 10 µM

final concentration), MBP-RebF (0.005 equiv., 2.5 μM final concentration), and glucose dehydrogenase (9 U/mL final concentration) were added as solutions (25 mM HEPES, pH 7.4) to Eppendorf tubes. A solution containing tryptamine (1 equiv., 0.5 mM final concentration), FAD (0.2 equiv., 100 μM final concentration), NAD (0.2 equiv., 100 μM final concentration), NaCl (20 equiv., 10 mM final concentration), and glucose (40 equiv., 20 mM final concentration) was added to these tubes to initiate reaction. Final reaction volume was 75 μL. Reactions were agitated overnight at 650 rpm in an Eppendorf air bath at room temperature. These were quenched with 75 μL of methanol, centrifuged to remove precipitated protein, filtered, and analyzed by UPLC Method 1, as described in the General Procedures. Bioconversions with 0S showed higher conversion of tryptamine than wtRebH, as well as 2T + N470S (Fig. S5). For this reason, 0S was used as parent in round 1.

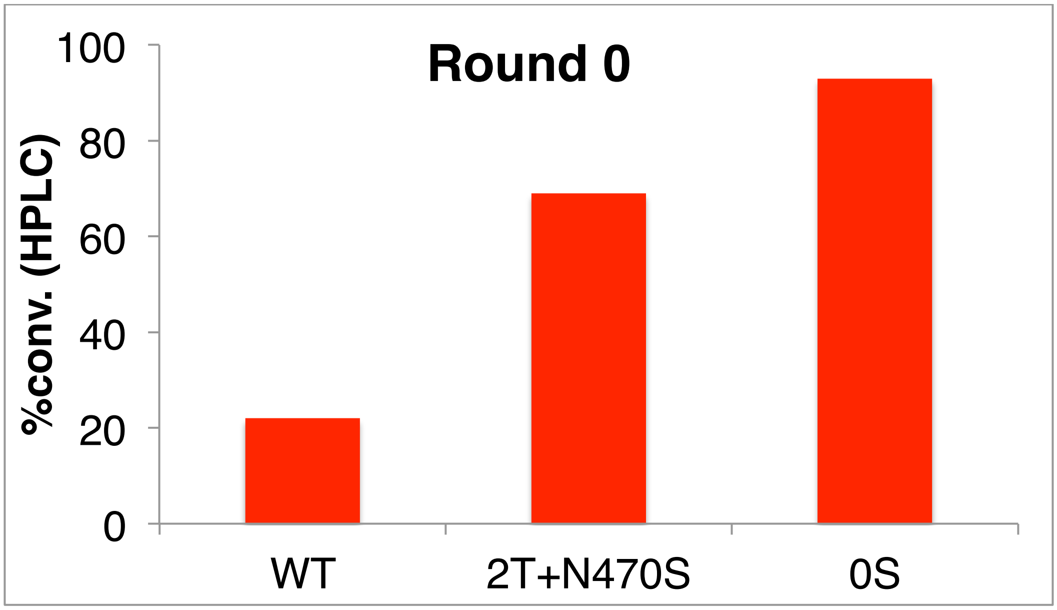

Figure S5: Conversion of tryptamine to 5-, 6- and 7-chlorotryptamine. When previously engineered RebH variants were screened for activity on tryptamine, mutants with mutation N470S gave the highest conversion. Point mutant 0S gives the highest conversion of all tested variants.

Rounds 1-6: For round 1, error-prone PCR was performed on the template 0S. From this 1,000 member library, one hit, 1P (0S + S448P) was identified by screening on Probe **1**, using the procedure outlined in the General Procedures (MALDI MS, followed by rescreen of hits by

HPLC). This mutant displayed a 4.5-fold increase in 5/6-chlorination of tryptamine over 0S (from 0.9% to 4.2% of total product). For round 2, error-prone PCR was performed on the template 1P. Two hits, 2RF and 2Q (1P + Q494R + L380F and 1P + R509Q) were identified from this 1,000 member library that had increased conversion of chlorotryptamine without a decrease in selectivity for 5/6 chlorination. These hits were identified through screening on Probe **1**, using the procedure outlined in the General Procedures (MALDI MS, followed by rescreen of hits by HPLC). The mutation R509Q was introduced into 2RF by overlap extension PCR (Primer sequences can be found in the Primer Table, PCR method described in section "Round 0"). The resulting 2RFQ variant gave the highest conversion to chlorotryptamine, without a decrease in selectivity for 5/6 chlorination. The bioconversions to confirm round 2 results were conducted in a similar manner to those performed for round 0. Tryptamine loading was increased to 2 mM, RebH loading was 10 μM, and reactions were agitated at 37 °C (Fig. S6).

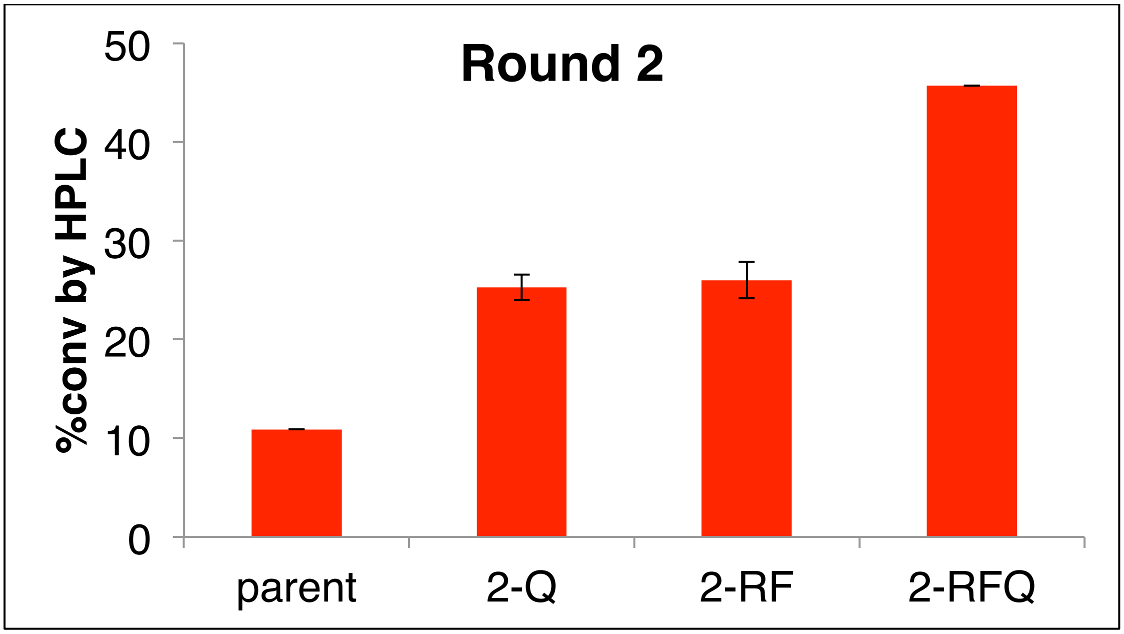

Figure S6: Conversion of tryptamine to 5-, 6- and 7-chlorotryptamine. When the mutations found in Round 2 are combined, their effects are roughly additive.

The point mutation Y455W[16] was then introduced into 2RFQ by overlap extension PCR (see "Round 0" and Primer Table). The variant, 3W, showed 1.7-fold increased selectivity for 5/6 chlorination of tryptamine over 2RFQ. For round 4, error-prone PCR was performed on the

template 3W. From this 1,000 member library, two hits were identified through screening on Probe **1**, using the procedure outlined in the General Procedures (MALDI MS, followed by rescreen of hits by HPLC), 4P and 4L (3W + S110P and 3W + F111L). 4P resulted in higher conversion to 5/6-chlorotryptamine without a decrease in selectivity, while 4L increased the selectivity for 5/6-chlorination of tryptamine. Addition of F111L to 4P by overlap extension PCR resulted in variant 4PL, which increased the conversion of variant 4L while retaining increased selectivity for 5/6-chlorination of tryptamine. The bioconversions to confirm round 4 results were conducted in a similar manner to those performed for round 0. Tryptamine loading was increased to 1.5 mM, RebH loading was 10 µM, and reactions were agitated at 25 °C (Fig. S7).

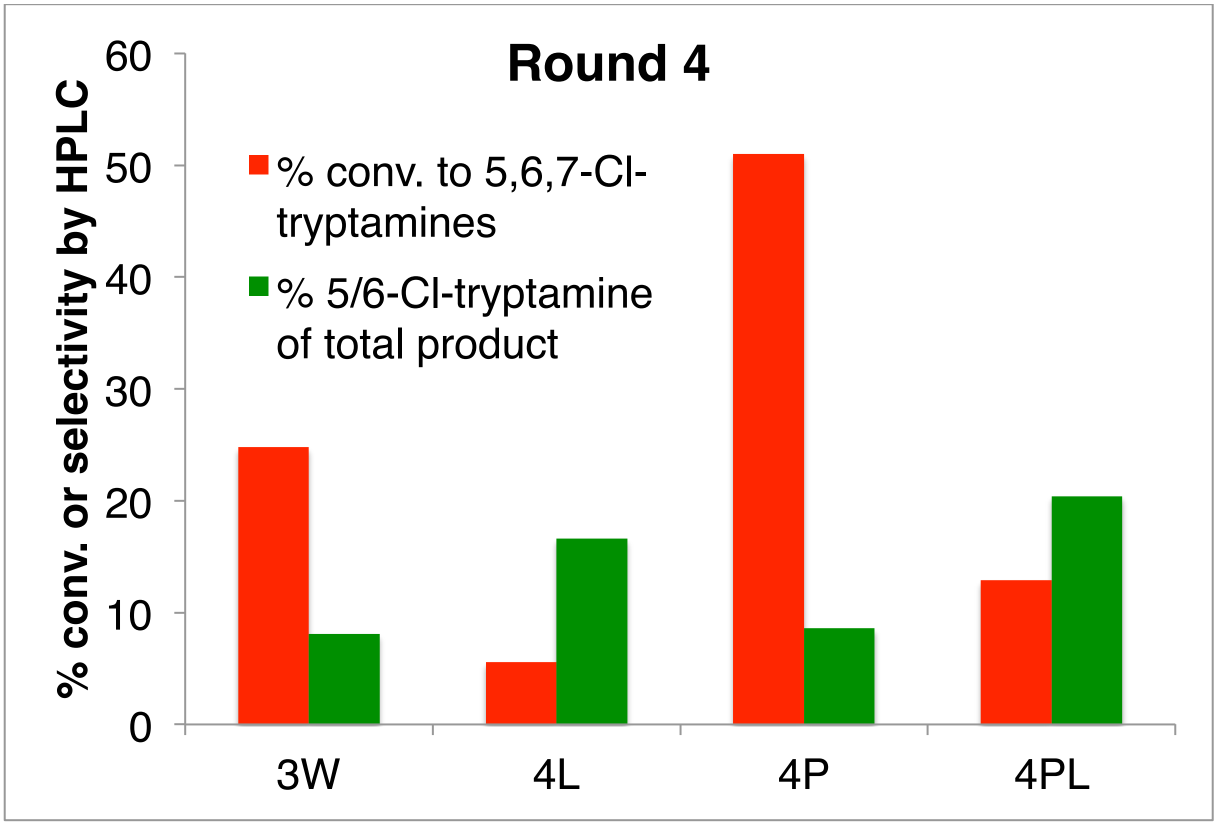

Figure S7: Conversion of tryptamine to 5-, 6- and 7-chlorotryptamine and selectivity of variants for 5- and 6-chlorination of tryptamine. When the two mutations found in round 4 are combined, the resulting variant, 4PL, retains the higher selectivity of 4L with increased conversion of tryptamine.

Point mutations N166S and S130L, which had previously been shown to increase the thermostability of RebH[10a], were introduced into 4PL by overlap extension PCR (see "Round 0" and Primer Table). The resulting mutant 5LS showed higher conversion to 5-,6-, and 7-chlorotrypamine without a decrease in selectivity for 5/6 chlorination (Fig. S14). For round 6, error-prone PCR was performed on the template 5LS. From this 1,000 member library, three hits were identified through screening on Probe **1**, using the procedure outlined in the General Procedures (MALDI MS, followed by rescreen of hits by HPLC). These were 6ILLA (5LS + T322I +F458L + F465L + V481A), 6TA (5LS + I52T + T496A), and 6VS (5LS + A58V + L111S). All three showed significantly increased selectivity for 5/6 chlorination of tryptamine. By examining the wtRebH crystal structure, the residues I52T, L111S and F465L were identified as active site residues. For this reason, 6T (5LS + I52T), 6S (5LS + L111S) and 6L (5LS + F465L) were cloned by overlap extension PCR. Indeed, these were the residues responsible for the observed changes in selectivity, and eliminating the additional mutations in each of the variants identified generally provided higher conversions. Next, all combinations of these three residues were cloned by overlap extension PCR. Bioconversions were conducted using Probe **2** to determine 5-, 6- and 7-chlorination selectivities (for detailed description of selectivity analysis, see section entitled "Conversion and selectivity determination of lineage"). From these bioconversions, it was found that 6S showed the best selectivity for 6-halogenation, while 6TL (5LS + I52T + F465L) showed the best selectivity for 5-halogenation (Fig. S14).

Rounds 7-10:

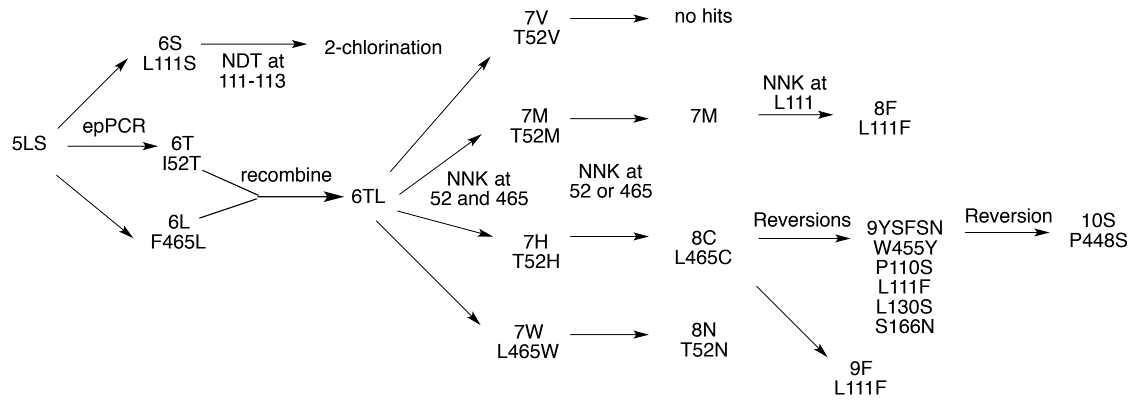

Figure S8: An expanded lineage summary of rounds 6-10.

Using 6S as a parent, residues 111-113 were randomized through overlap extension PCR using NDT codons. From this, a library of 1,000 variants was generated, expressed and screened on Probe **1**, using the procedure outlined in the General Procedures (MALDI MS, followed by rescreen of hits by HPLC). Hits with high selectivity for 6-chlorination from this library were initially identified, but upon further analysis it was found that these hits produced high amounts of 2-oxotryptamine. 2-Oxotryptamine can be formed by chlorination at C2 of tryptamine, followed by hydrolysis. To confirm that this process could happen readily, a chemical chlorination of tryptamine with NCS was conducted (see "detailed isolation and characterization"). Upon reverse phase Biotage purification using conditions similar to both UPLC analytical methods, only 2-oxotryptamine was recovered. MALDI MS data for variants along the selectivity lineage were re-evaluated for the presence of 2-oxotryptamine. Only mutants with the F111S mutation generated substantial amounts of this product. Because of this, the branch of the lineage containing 6S was no longer pursued, and efforts were focused on the I52T and F465L mutations (Fig. S8).

Because variant 6TL produced similar amounts of 5-, 6-, and 7-chlorotryptamines, it was a promising candidate for both the 5- and 6-chlorination branches of the lineage. Two libraries were generated by individual randomization of residues 52 and 465 in 6TL by site directed mutagenesis with NNK codons (detailed procedure found in the General Procedures). 100-150 variants were screened for each library, corresponding to over 95% coverage.[12] Libraries were screened for activity on Probe **2** using sequential MALDI-MS/UPLC, as described in the General Procedures. Many hits were identified, including 7M (T52M), 7H (T52H), 7W (L465W) and 7V (T52V). The bioconversions to confirm round 7 results were conducted and analyzed in a similar manner to those in the "conversion and selectivity determination of lineage" section. Probe **2** was used as substrate and was added to a final concentration of 1.5 mM, RebH loading was 10 μM, and reactions were agitated at 25 °C. Reactions were analyzed by LC-MS using the LC-MS Method 1 described in the General Procedures. Results are shown in Figure S9.

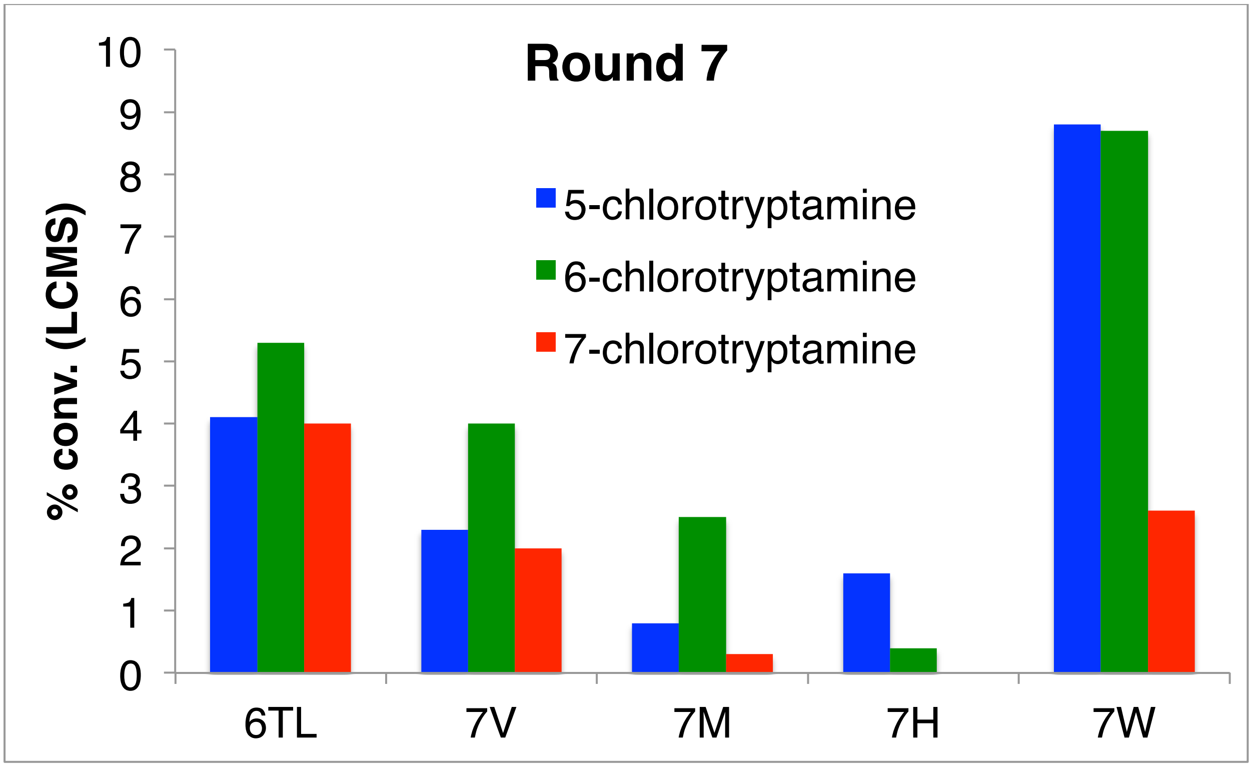

Figure S9: Conversion of tryptamine to 5-, 6- and 7-chlorotryptamine. All four variants show increased selectivity for either 5- or 6-chlorotryptamine and were carried onto round 8.

The four hits 7M, 7H, 7W and 7V were used as templates for the next round. Three libraries were generated by individual randomization of residue 465 in 7M, 7H and 7V by site directed mutagenesis with NNK codons. A fourth library was generated by individual randomization of residue 52 in 7W by site directed mutagenesis with NNK codons. (detailed procedure found in the General Procedures). 100-150 variants were screened for each library, corresponding to over 95% coverage.[12] Libraries were screened for activity on Probe **2** using sequential MALDI-MS/UPLC, as described in the General Procedures. No hits were found for the 7V and 7M libraries. Mutant 8N (T52N) from the 7W library and mutant 8C (L465C) from the 7H library were identified. The bioconversions to confirm round 8 results were conducted in the same manner as those for round 7. The mutants with the highest selectivities for 5 and 6-chlorination of tryptamine were 8C and 7M, respectively (Fig. S10).

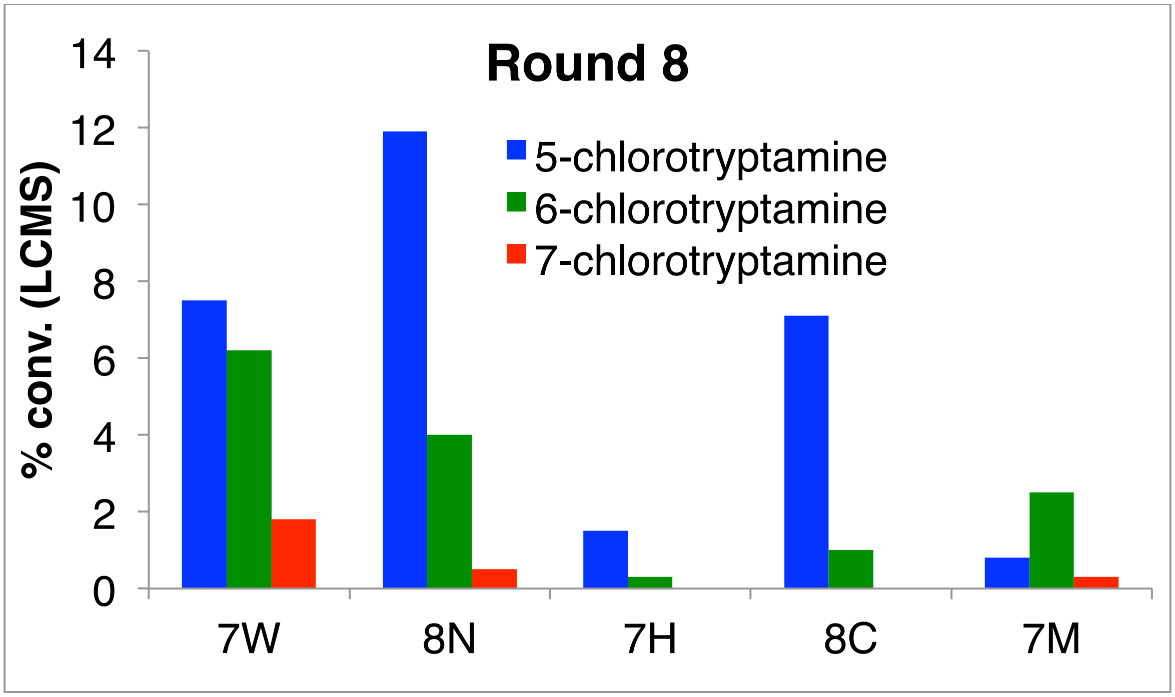

Figure S10: Conversion of tryptamine to 5-, 6- and 7-chlorotryptamine. 8C shows the best selectivity for 5-halogenation. 7M has the best selectivity for 6-halogenation.

While 7M was selective for 6-halogention, it suffered from low activity. Since residue 111 had previously affected both the selectivity and activity so substantially (F111L and L111S), a library was generated by individual randomization of residue 111 in 7M by site directed mutagenesis with NNK codons (detailed procedure found in the General Procedures). 150 variants were screened, corresponding to over 95% coverage.[12] Libraries were screened for activity on Probe **2** using sequential MALDI-MS/UPLC, as described in the General Procedures. One hit, 8F (7M + L111F) was identified. The bioconversions to confirm this hit were conducted in the same manner as those for round 7 (Fig. S11).

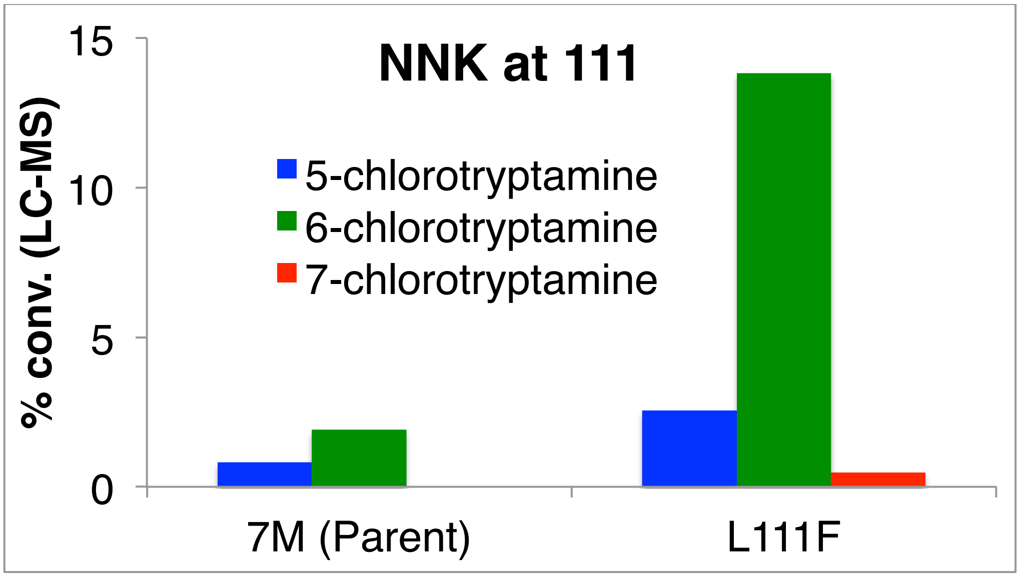

Figure S11: Conversion of tryptamine to 5-, 6- and 7-chlorotryptamine. When the mutation L111F is introduced into 7M, the conversion and selectivity for 6-chlorotryptamine both increase.

In an effort to increase the conversion of 8C, the L111F mutation was introduced by overlap extension PCR. While this did slightly increase the conversion, it still remained too low for preparative reactions. For this reason, other reversions were made. The I52H and F465C mutations (from rounds 7-8) were introduced into every parent along the lineage (0S, 1P, 2RFQ, 3W and 4PL). From these variants, 9YSFSN (2RFQ + I52H + F465C) gave the highest conversion with no loss of selectivity. The S448P mutation led to a loss of conversion when introduced into 0S (forming 1P); therefore, this mutation was reverted in 9YSFSN. The resulting variant, 10S, had increased conversion of tryptamine without loss of selectivity.

Other targeted libraries: In addition to the libraries discussed in rounds 1-8, three other targeted libraries were constructed along this lineage that produced no hits. These were NDT libraries at residues P53, Y362 and W466. The parent template for P53 and W466 was variant 6TL. 5LS was used as template for Y362. Reactions were conducted with Probe **1**, and were screened by sequential MALDI MS/UPLC, as described in the General Procedures.

Conversion and selectivity determination of lineage: In order to compare the conversions and selectivities for 5-, 6- and 7-chlorination of tryptamine of key mutants along the lineage, 50 mL

expression cultures were grown for the following variants: wtRebH, 0S, 1P, 2RFQ, 3W, 4PL, 5LS, 6L, 6T, 6TL, 6S, 7M, 8F, 7H, 8C, 9YSFSN, 10S. These cultures were expressed, lysed, and purified according to the protocol found in the General Procedures.

Reaction set-up for wtRebH, 0S, 1P, 2RFQ, 3W: RebH variants (0.05 equiv., 15 μM final concentration) were arrayed into a 96-well microtiter plate in triplicate. MBP-RebF (0.0017 equiv., 2.5 μM final concentration) and glucose dehydrogenase (9 U/mL final concentration) were added as solutions (25 mM HEPES, pH 7.4) to the RebH. A solution containing Probe **2** (1 equiv., 1.5 mM final concentration), NAD (0.067 equiv., 100 μM final concentration), FAD (0.067 equiv., 100 μM final concentration), NaCl (66.7 equiv., 100 mM final concentration), phenol (internal standard, 0.33 equiv., 0.5 mM final concentration) and glucose (13.3 equiv., 20 mM final concentration) were added via multichannel pipette to simultaneously initiate the reactions (final reaction volume of 75 μL).

Reaction set-up for 3W, 4PL, 5LS, 6L, 6T, 6TL, 6S, 7M, 8F, 7H, 8C, 9YSFSN, 10S: RebH variants (0.05 equiv., 25 μM final concentration) were arrayed into a 96-well microtiter plate in triplicate. MBP-RebF (0.005 equiv., 2.5 μM final concentration) and glucose dehydrogenase (9 U/mL final concentration) were added as solutions (25 mM HEPES, pH 7.4) to the RebH. A solution containing Probe **2** (1 equiv., 0.5 mM final concentration), NAD (0.2 equiv., 100 μM final concentration), FAD (0.2 equiv., 100 μM final concentration), NaCl (200 equiv., 100 mM final concentration), phenol (internal standard, 1 equiv., 0.5 mM final concentration) and glucose (40 equiv., 20 mM final concentration) were added via multichannel pipette to simultaneously initiate the reactions (final reaction volume of 75 μL).

Reactions for all mutants were mixed at 650 rpm on top of an Eppendorf air bath and were quenched with 1 volume (75 μL) methanol after 16 hours. The precipitated protein was removed by centrifugation and the reactions were filtered and analyzed by LC-MS using Method 1 from the General Procedures. 7-chlorotryptamine separates from 5- and 6-chlorotryptamine using this LC-MS method. Because of this, concentrations of 7-chlorotryptamine were determined by calculating the ratio of product to internal standard and fitting that value to a calibration curve. 5- and 6-chlorotryptamines do not separate by LC-MS. To find the ratio of 5:6 chlorinated product, the mass intensities m/z = 195 and 196 for the 5/6-chlorotryptamine peak were analyzed. The 196 peak was adjusted according to the predicted spectrum for 5-chlorotryptamine (the intensity of m/z = 196 – (the intensity of m/z = 195 * 0.12)). The ratio of

195:196 was then calculated using these values (intensity of 195 and adjusted intensity of 196). This ratio was then used to find the area of the UV peak corresponding to the individual 5- and 6-chlorotryptamines. These two values were divided by the area for internal standard and fit to a calibration curve.

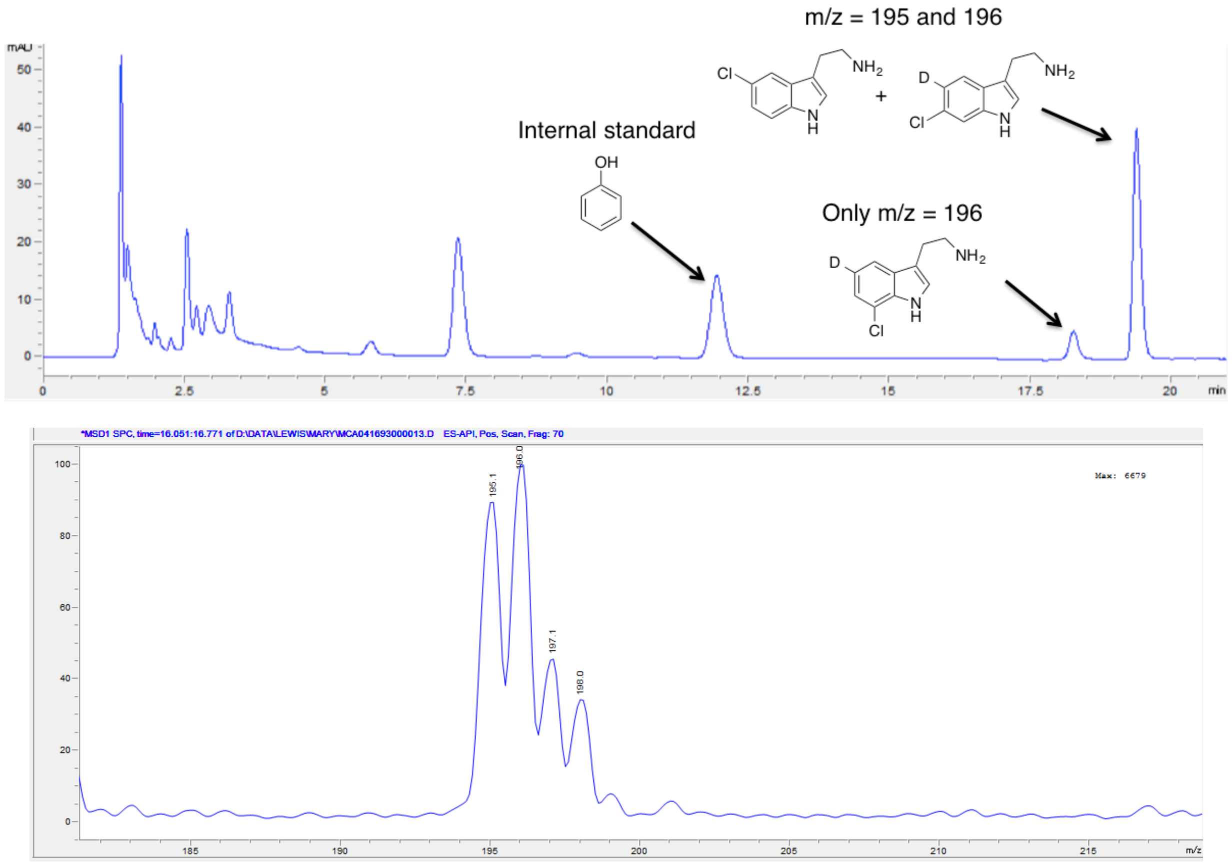

Figure S12: Top: An example LC-MS trace for a RebH bioconversion. 7-chlorotryptamine is chromatographically separated from the 5- and 6-chlorotryptamines. Bottom: Mass spectrum of peak for 5- and 6-chlorotryptamines. When Probe 2 is used, the difference between the masses of 5- and 6-chlorotryptamines can be used to calculate the ratio of 5:6.

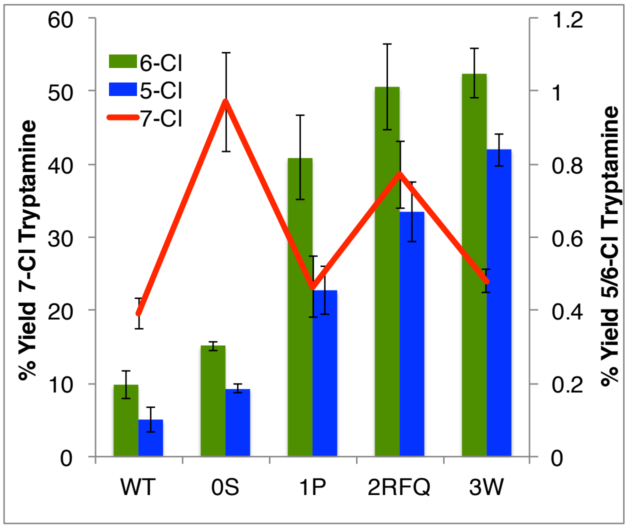

Figure S13: Yield of 5, 6 and 7 chlorotryptamines for variants RebH - 3W. As the lineage progresses, both yield of 5- and 6-chlorotryptamines as well as selectivity for 5- and 6-chlorotryptamines increase.

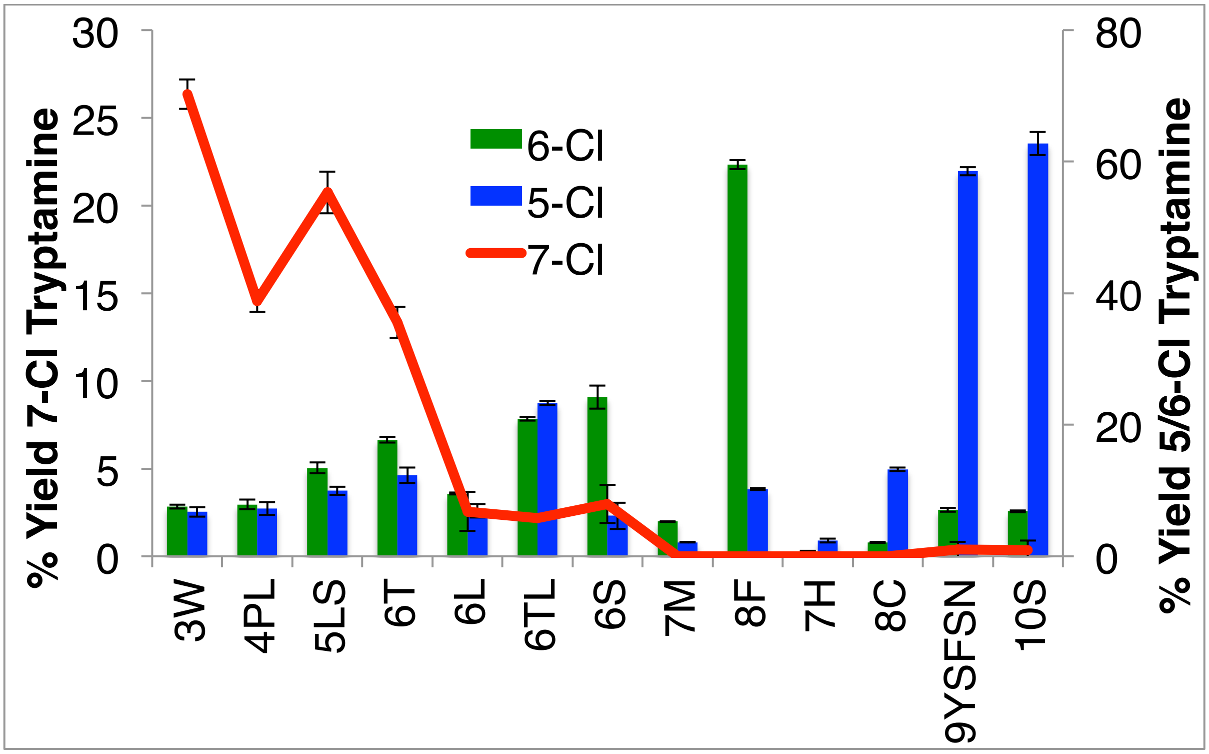

Figure S14: Yield of 5, 6 and 7 chlorotryptamines for variants 3W - 10S. As the lineage progresses, both yield of as well as selectivity for 5- and/or 6-chlorotryptamines increase.

Conversion and selectivity determination for bromination reactions: Bioconversions were conducted similarly to the procedure detailed in "conversion and selectivity determination of

lineage." Reactions were conducted with NaBr (100 mM final concentration) instead of NaCl and 25 μM of each halogenase. Reactions were quenched with one volume methanol after 12 hours and were analyzed similarly to the procedure detailed in "conversion and selectivity determination of lineage." Instead of using the peaks corresponding to m/z = 195 and 196, the peaks corresponding to m/z = 239 and 240 were used to calculate the selectivity of 8F and 10S.

Determination of kinetic parameters: Rates were determined by monitoring the conversion of 2-4500 μM tryptamine in the presence of NAD (100 μM final concentration), FAD (100 μM final concentration), NaCl (100 mM final concentration), MBP-RebF (2.5 μM final concentration), glucose dehydrogenase (9 U/mL final concentration GDH), glucose (20 mM final concentration), and phenol as an internal standard (0.5 mM final concentration) at a final volume of 75 μL in a microtiter plate. RebH was added at a final concentration of either 0.1 μM (0S) or 25 μM (10S, and 8F). Plates were sealed using a plate sealer and shaken at 650 rpm at room temperature. Reaction mixtures were quenched at 10-60 minutes by addition of 75 μL of MeOH. All time points were collected in triplicate. The precipitated protein was then removed by centrifugation and the reactions were filtered and analyzed by HPLC method 2 described in the General Procedures. Product formation was determined by calculating the ratio of product to internal standard and fitting that value to a calibration curve prepared from known concentrations of each chlorinated isomer of tryptamine. The kinetic parameters (K_m and k_{cat}) for each mutant were determined using the Hanes-Woolf plots constructed from the substrate concentrations and the observed initial rates.

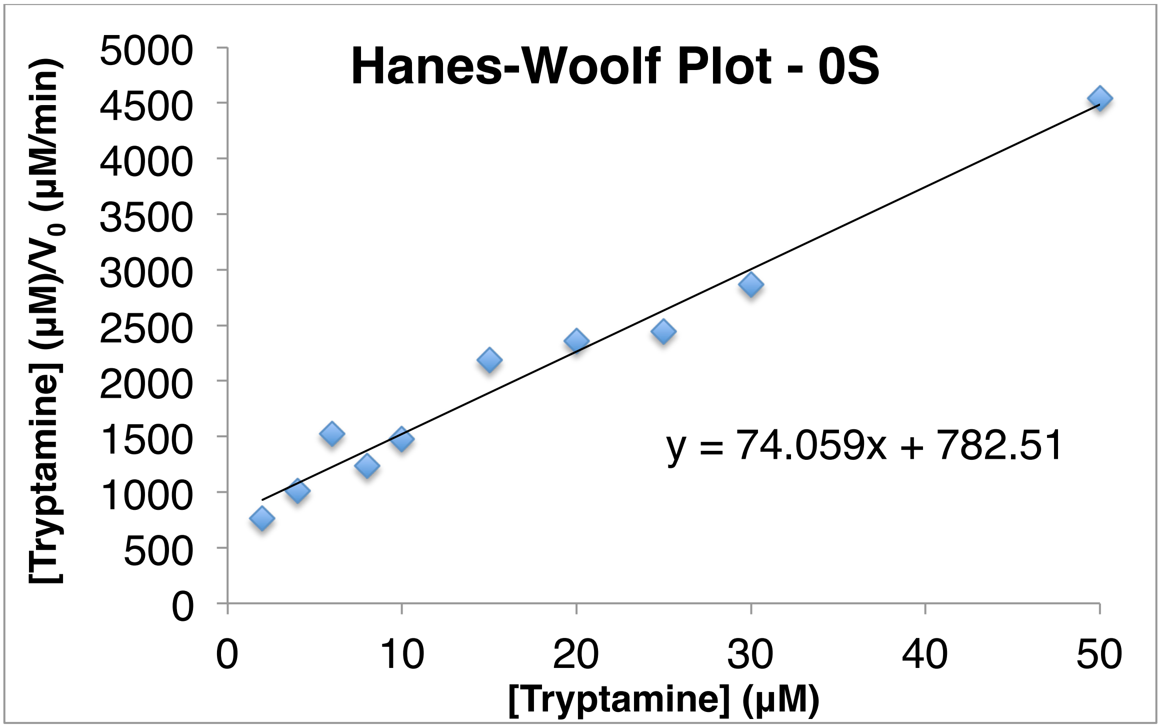

Figure S15: Hanes-Woolf plot for variant 0S. From this, K_M and k_{cat} were calculated to be 10.6 μM and 0.135 min^{-1}, respectively.

Figure S16: A

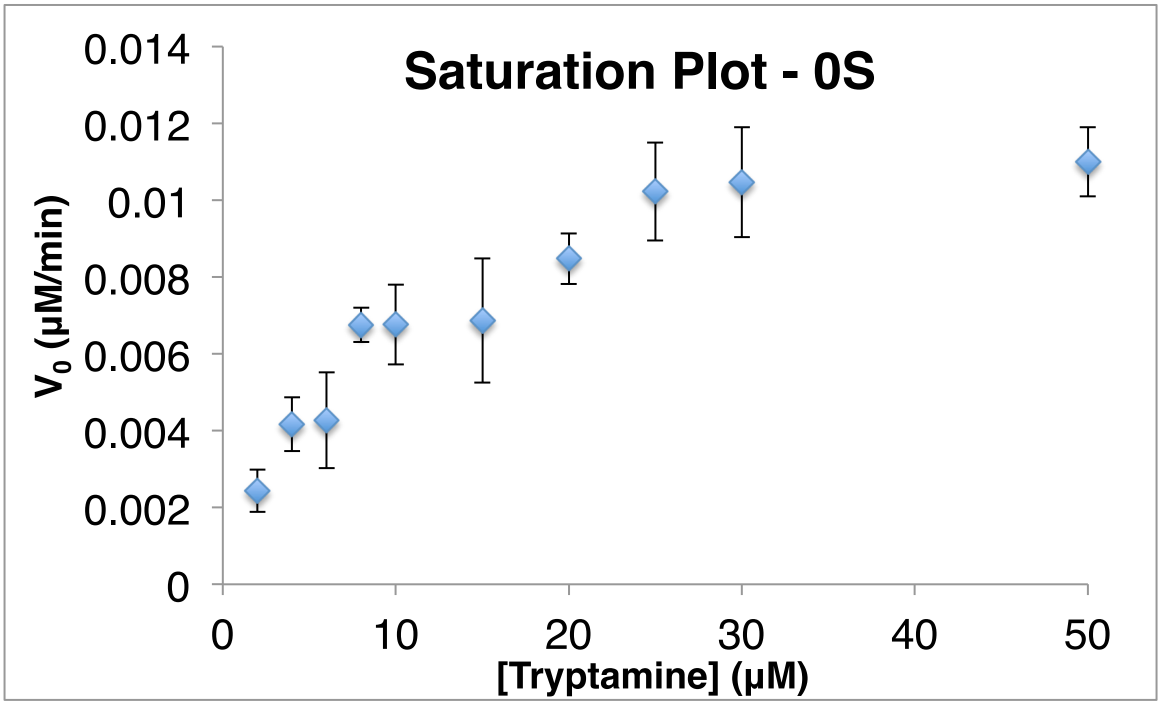

Figure S16: B

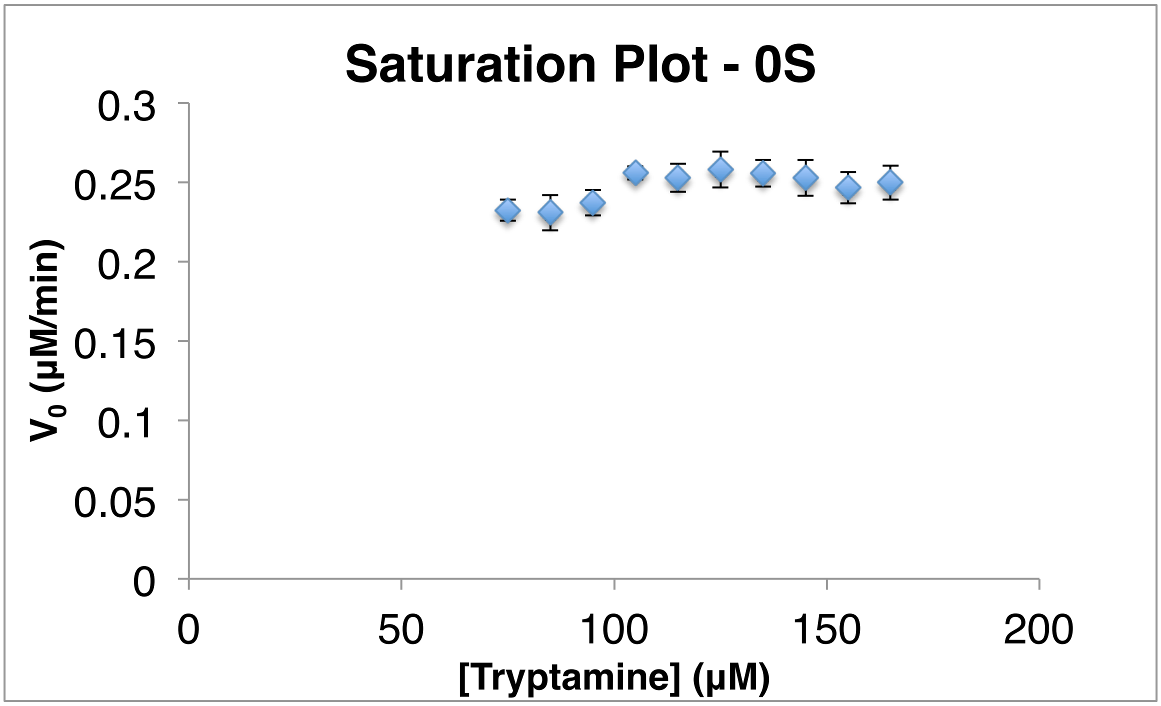

Figure S16: A. Saturation plot of data used for Hanes-Woolf plot (Fig. S15) for variant 0S. Standard deviations between trials are displayed. B. Saturation plot for kinetic data obtained at higher substrate concentration range. No substrate inhibition is observed at these higher concentrations.

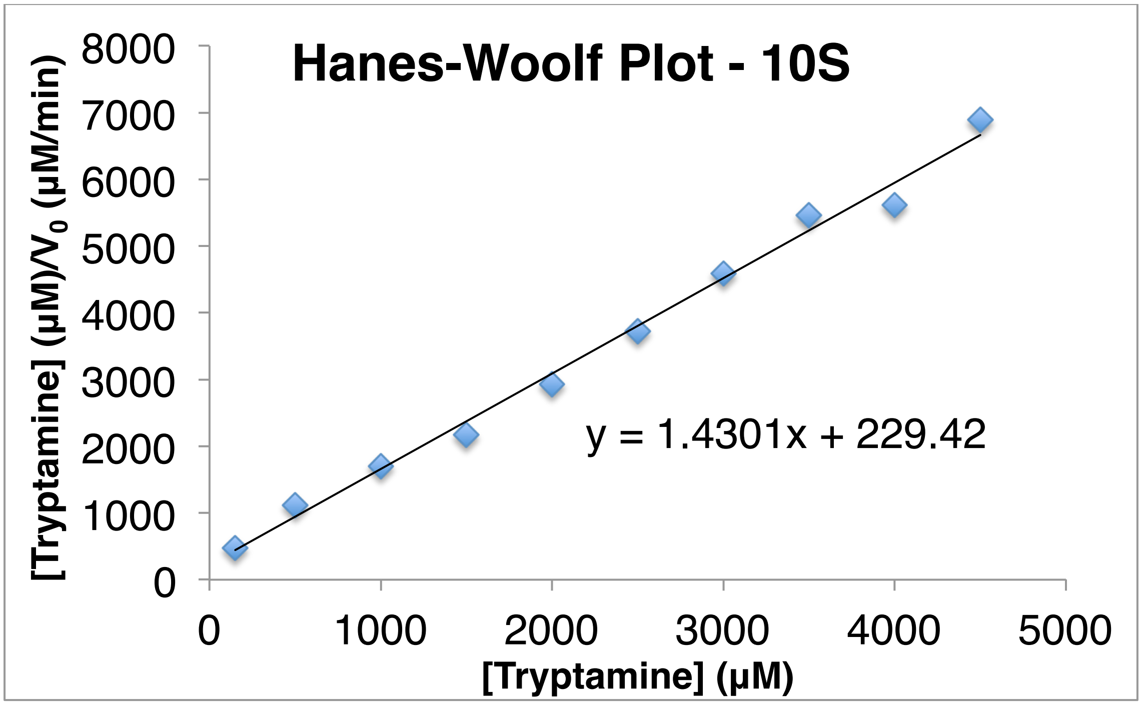

Figure S17: Hanes-Woolf plot for variant 10S. From this, K_M and k_{cat} were calculated to be 160 µM and 0.028 min^{-1}, respectively.

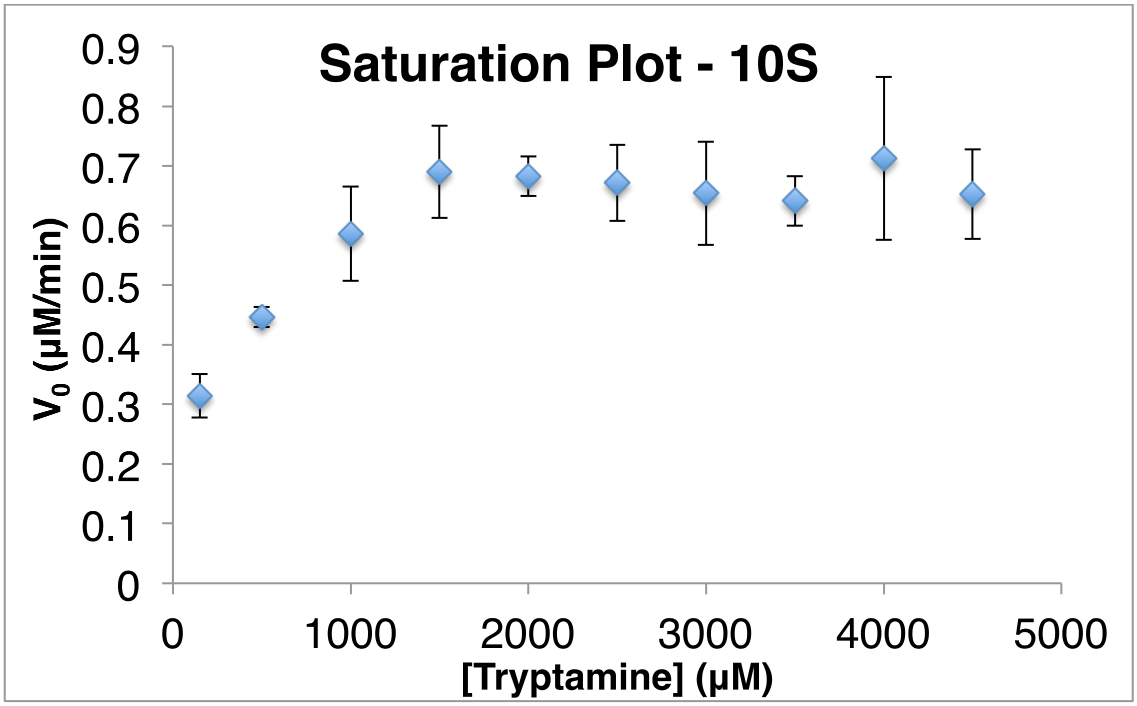

Figure S18: Saturation plot for variant 10S. Standard deviations between trials are displayed.

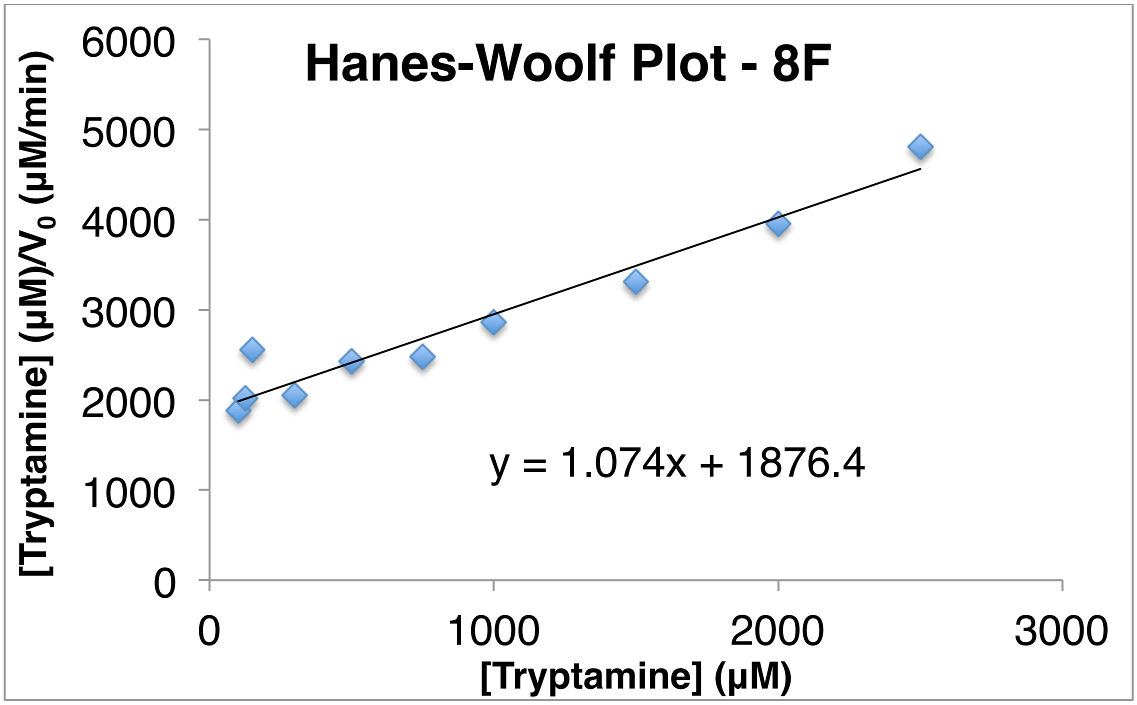

Figure S19: Hanes-Woolf plot for variant 8F. From this, K_M and k_{cat} were calculated to be 1747 μM and 0.037 min^{-1}, respectively.

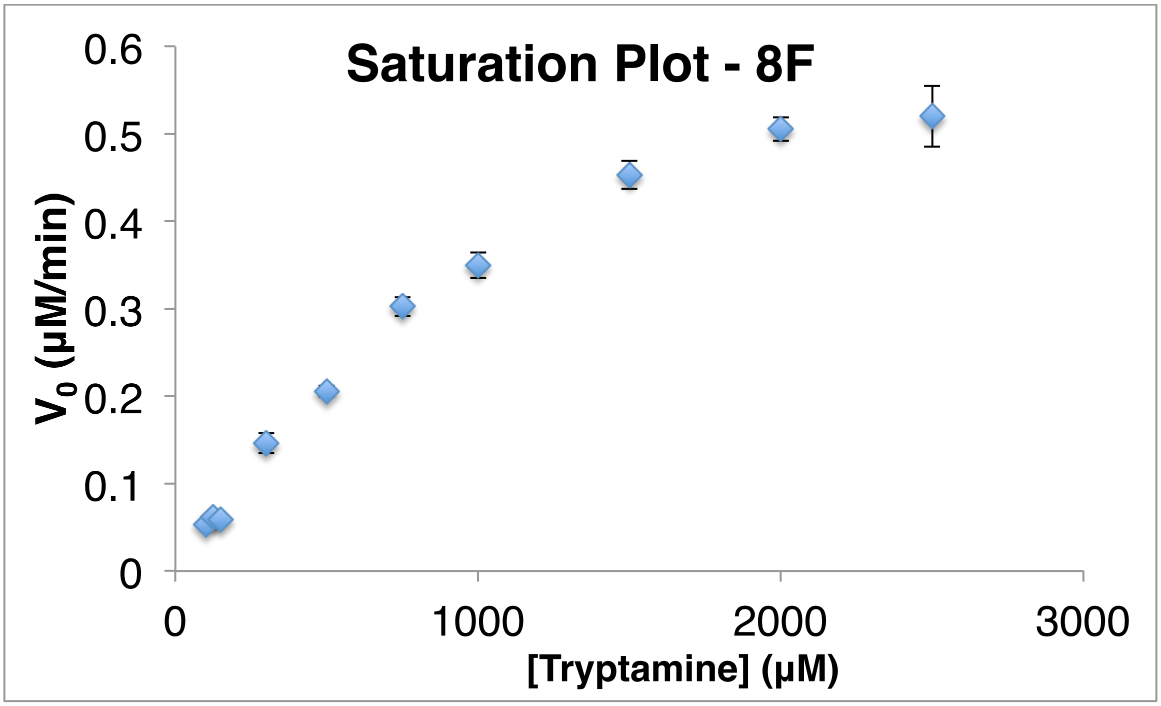

Figure S20: Saturation plot for variant 8F. Standard deviations between trials are displayed.

Detailed isolation and characterizations:

7-chloro-tryptamine (3): The bioconversion was conducted in a crystallization dish according to the general procedure, using 0.02 equiv. of 0S (10 µM final concentration) at 25 °C, with 10 mg of substrate and 10 mM NaCl. After maximum conversion to monohalogenated product was observed by HPLC, the reaction mixture was filtered through Celite, extracted into CH_2Cl_2, and concentrated onto Celite. The Celite was packed into a Biotage samplet, which was then loaded into a reverse phase column (Biotage SNAP-KP-C18-HS). The crude material was purified by reverse phase chromatography (gradient from 100% H_2O to 15% CH_3CN/H_2O) to afford the known compound[4] **3** in 98% yield (19.0 mg of **3**·TFA, 0.0617 mmol). ^1H NMR (500 MHz; MeOD): δ 7.49 (dd, J = 7.9, 0.8, 1H), 7.22 (s, 1H), 7.11 (d, J = 7.6, 1H), 6.99 (t, J = 7.8, 1H), 3.19 (t, J = 7.4, 2H), 3.08 (t, J = 7.4, 2H). HRMS (ESI-TOF) calcd for $C_{10}H_{11}N_2Cl$ $[M + H]^+$: 195.0684 and 197.0654, found: 195.0682 and 197.0658.

6-chloro-tryptamine (5): The bioconversion was conducted in a crystallization dish according to the general procedure, using 0.1 equiv. of 8F (50 µM final concentration) at 16 °C, with 10 mg of substrate and 10 mM NaCl. After maximum conversion to monohalogenated product was observed by HPLC, the reaction mixture was filtered through Celite, extracted into CH_2Cl_2, and concentrated onto Celite. The Celite was packed into a Biotage samplet, which was then loaded into a reverse phase column (Biotage SNAP-KP-C18-HS). The crude material was purified by reverse phase chromatography (gradient from 100% H_2O to 15% CH_3CN/H_2O) to afford the known compound[5] **5** in 73% yield (14.1 mg of **5**·TFA, 0.0456 mmol). ^1H NMR (500 MHz; MeOD): δ 7.53 (d, J = 8.4, 1H), 7.38 (d, J = 1.8, 1H), 7.20 (s, 1H), 7.03 (dd, J = 8.5, 1.9, 1H), 3.22 (t, J = 7.4, 2H), 3.10 (t, J = 7.4, 2H). HRMS (ESI-TOF) calcd for $C_{10}H_{11}N_2Cl$ $[M + H]^+$: 195.0684 and 197.0654, found: 195.0736 and 197.0669.

5-chloro-tryptamine (6): The bioconversion was conducted according to the general procedure using 0.1 equiv. of 10S (50 µM final concentration) at 10 °C, with 10 mg of substrate and 100 mM NaCl. After maximum conversion to monohalogenated product was observed by HPLC, the reaction mixture was filtered through Celite, extracted into CH_2Cl_2, and concentrated onto Celite. The Celite was packed into a Biotage samplet, which was then loaded into a reverse phase column (Biotage SNAP-KP-C18-HS). The crude material was purified by reverse phase chromatography (gradient from 100% H_2O to 15% CH_3CN/H_2O) to afford the known

compound[6] **6** in 78% yield (15.0 mg of **6**·TFA, 0.0487 mmol). ^{1}H NMR (500 MHz; MeOD): δ 7.54 (d, J = 2.0, 1H), 7.30 (d, J = 8.6, 1H), 7.19 (s, 1H), 7.05 (dd, J = 8.6, 2.0, 1H), 3.17 (t, J = 7.4, 2H), 3.04 (t, J = 7.4, 2H). HRMS (ESI-TOF) calcd for $C_{10}H_{11}N_2Cl$ $[M + H]^+$: 195.0684 and 197.0654, found: 195.0687 and 197.0655.

2-oxytryptamine (13): The prodecure was adapted form a previous report.[17] Tryptamine (100 mg, 0.625 mmol, 1 equiv.) and *N*-chlorosuccinimide (83.4 mg, 0.625 mmol, 1 equiv.) were added to a 25 mL round bottom flask. A mixture of glacial acetic acid and formic acid (10:3, 7.9 mL) was added and the reaction was stirred for 20 minutes at room temperature. The reaction was neutralized with NaOH, extracted into CH_2Cl_2, and concentrated onto Celite. The Celite was packed into a Biotage samplet, which was then loaded into a reverse phase column (Biotage SNAP-KP-C18-HS). The crude material was purified by reverse phase chromatography (gradient from 100% H_2O to 15% CH_3CN/H_2O) to afford the known compound **13**[7]. No 2-chlorotryptamine was observed upon purification. ^{1}H NMR (500 MHz; MeOD): δ 7.30 (dd, J = 7.4, 0.6, 1H), 7.25 (tt, J = 7.7, 1.0, 1H), 7.06 (td, J = 7.6, 1.0, 1H), 6.92 (d, J = 7.8, 1H), 3.64 (dd, J = 8.4, 5.1, 1H), 3.14 (t, J = 7.6, 2H), 2.35-2.29 (m, 1H), 2.14-2.07 (m, 1H). HRMS (ESI-TOF) calcd for $C_{10}H_{12}N_2O$ $[M + H]^+$: 177.1022, found: 177.1011.

Substrate scope characterization:

^{1}H NMR and high-resolution mass spectrometry were used to characterize products isolated from 1-2 mg reactions. Because of the small amounts of isolated product, ^{13}C NMR was not obtained. Reported conversion values were obtained from raw HPLC data. No isolated yields were obtained. Selectivities were determined by comparing ^{1}H NMR integrations of similar aryl protons for the compounds.

6-chloro-2-methyl-tryptamine (7): The bioconversion was conducted in a flask according to the general procedure using 0.08 equiv. of 8F (40 μM final concentration) at 16 °C, with 1 mg of substrate and 10 mM NaCl. After maximum conversion to monohalogenated product was observed by HPLC, the reaction mixture was filtered through Celite, extracted into CH_2Cl_2, and dried under high vacuum. The crude material was re-suspended in 200 μL of MeOH, filtered, and purified by preparative reverse phase HPLC in 50 μL increments according to HPLC method 1. Product-containing fractions were pooled to afford **7.** ^{1}H NMR (500 MHz; MeOD): δ 7.41 (d,

J = 8.4, 1H), 7.27 (s, 1H), 6.99 (dd, J = 8.4, 1.8, 1H), 3.13 (t, J = 7.3, 2H), 3.04 (t, J = 7.6, 2H), 2.40 (s, 3H). HRMS (ESI-TOF) calcd for $C_{11}H_{13}N_2Cl$ $[M + H]^+$: 209.0840 and 211.0811, found: 209.0851 and 211.0824.

5-chloro-2-methyl-tryptamine (8) and 6-chloro-2-methyl-tryptamine (7): The bioconversion was conducted in a flask according to the general procedure using 0.08 equiv. of 10S (40 μM final concentration) at 16 °C, with 1 mg of substrate and 100 mM NaCl. After maximum conversion to monohalogenated product was observed by HPLC, the reaction mixture was filtered through Celite, extracted into CH_2Cl_2, and dried under high vacuum. The crude material was re-suspended in 200 μL of MeOH, filtered, and purified by preparative reverse phase HPLC in 50 μL increments according to HPLC method 1. Product-containing fractions were pooled to afford a mixture of **7** and **8.** ^{1}H NMR (500 MHz; MeOD): δ 7.47 (d, J = 1.8, 0.168H), 7.41 (d, J = 8.4, 0.642H), 7.27 (d, J = 1.9, 0.476H), 7.23 (d, J = 8.6, 0.158H), 7.02 (dd, J = 8.32, 2.26, 0.209H), 6.99 (ddd, J = 8.4, 1.9, 0.7, 0.529H), 3.12 (m, 2H), 3.03 (m, 2H), 2.40 (m, 3H). HRMS (ESI-TOF) calcd for $C_{11}H_{13}N_2Cl$ $[M + H]^+$: 209.0840 and 211.0811, found: 209.0844 and 211.0840.

6-chloro-N-methyl-tryptamine (9): The bioconversion was conducted in a flask according to the general procedure using 0.1 equiv. of 8F (50 μM final concentration) at 16 °C, with 2 mg of substrate and 10 mM NaCl. After maximum conversion to monohalogenated product was observed by HPLC, the reaction mixture was filtered through Celite, extracted into CH_2Cl_2, and dried under high vacuum. The crude material was re-suspended in 400 μL of MeOH, filtered, and purified by preparative reverse phase HPLC in 50 μL increments according to HPLC method 1. Product-containing fractions were pooled to afford **9.** ^{1}H NMR (500 MHz; MeOD): δ 7.54 (d, J = 8.5, 1H), 7.39 (d, J = 1.8, 1H), 7.22 (m, 1H), 7.05 (dd, J = 8.5, 1.9, 1H), 3.13 (t, J = 7.4, 2H), 2.71 (s, 3H). HRMS (ESI-TOF) calcd for $C_{11}H_{13}N_2Cl$ $[M + H]^+$: 209.0840 and 211.0811, found: 209.0850 and 211.0818.

5-chloro-N-methyl-tryptamine (10): The bioconversion was conducted in a flask according to the general procedure using 0.1 equiv. of 10S (50 μM final concentration) at 16 °C, with 2 mg of substrate and 100 mM NaCl. After maximum conversion to monohalogenated product was observed by HPLC, the reaction mixture was filtered through Celite, extracted into CH_2Cl_2, and dried under high vacuum. The crude material was re-suspended in 400 μL of MeOH, filtered,

and purified by preparative reverse phase HPLC in 50 µL increments according to HPLC method 1. Product-containing fractions were pooled to afford **10.** ^{1}H NMR (500 MHz; MeOD): δ 7.60 (d, J = 1.9, 1H), 7.35 (d, J = 8.6, 1H), 7.25 (s, 1H), 7.11 (dd, J = 8.6, 1.9, 1H), 3.29 (m, 2H), 3.12 (t, J = 7.4, 2H). HRMS (ESI-TOF) calcd for $C_{11}H_{13}N_2Cl$ $[M + H]^+$: 209.0840 and 211.0811, found: 209.0855 and 211.0823.

6-chloro-tryptophol (11): The bioconversion was conducted in a flask according to the general procedure using 0.1 equiv. of 8F (50 µM final concentration) at 16 °C, with 2 mg of substrate and 10 mM NaCl. After maximum conversion to monohalogenated product was observed by HPLC, the reaction mixture was filtered through Celite, extracted into CH_2Cl_2, and dried under high vacuum. The crude material was re-suspended in 400 µL of MeOH, filtered, and purified by preparative reverse phase HPLC in 50 µL increments according to HPLC method 1. Product-containing fractions were pooled to afford **11.** ^{1}H NMR (500 MHz; MeOD): δ 7.49 (dd, J = 8.4, 0.5, 1H), 7.32 (dd, J = 1.9, 0.5, 1H), 7.09 (m, 1H), 6.97 (dd, J = 8.4, 1.9, 1H), 3.79 (t, J = 7.2, 2H), 2.94 (td, J = 7.2, 0.8, 2H). HRMS (ESI-TOF) calcd for $C_{10}H_{10}NOCl$ $[M + H]^+$: 196.0524 and 198.0495, found: 196.0509 and 198.0531.

5-chloro-tryptophol (12): The bioconversion was conducted in a flask according to the general procedure using 0.1 equiv. of 10S (50 µM final concentration) at 16 °C, with 2 mg of substrate and 100 mM NaCl. After maximum conversion to monohalogenated product was observed by HPLC, the reaction mixture was filtered through Celite, extracted into CH_2Cl_2, and dried under high vacuum. The crude material was re-suspended in 400 µL of MeOH, filtered, and purified by preparative reverse phase HPLC in 50 µL increments according to HPLC method 1. Product-containing fractions were pooled to afford **12.** ^{1}H NMR (500 MHz; MeOD): δ 7.52 (s, 1H), 7.29 (d, J = 9.2, 1H), 7.13 (s, 1H), 7.04 (d, J = 8.6, 1H), 3.79 (t, J = 7.2, 2H), 2.93 (t, J = 7.1, 2H). HRMS (ESI-TOF) calcd for $C_{10}H_{10}NOCl$ $[M + H]^+$: 196.0524 and 198.0495, found: 196.0504 and 198.0530.

Effect of key selectivity-changing residues in wtRebH: To analyze the effects of the mutations I52T and F465L found in round 6, both mutations were independently introduced into the wtRebH gene through overlap extension PCR (as described in section "Round 0"), thus forming WT+T and WT+L. 50 mL expression cultures of WT+T, WT+L, wtRebH, 5LS, 6T and 6L were

grown, lysed and purified as described in the General Procedures. Reactions were conducted in duplicate and analyzed according to the procedure found in "Conversion and selectivity determination of lineage." Results are shown in Figure S21.

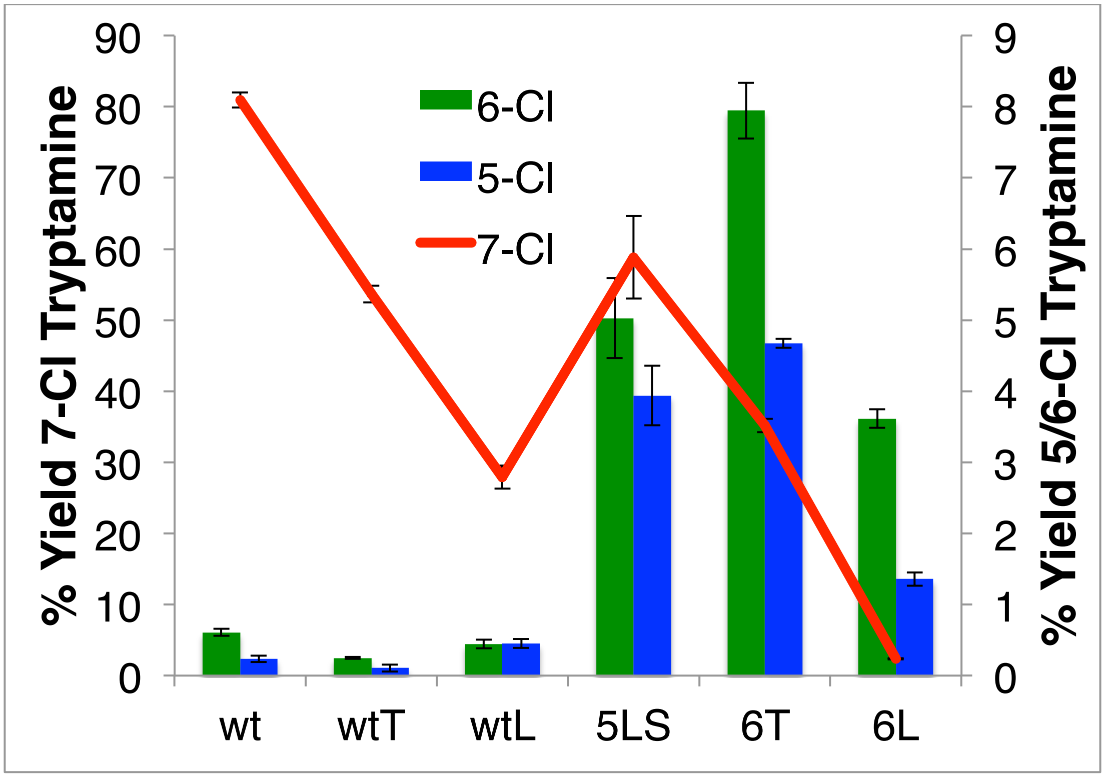

Figure S21: Yield of 5, 6 and 7 chlorotryptamines for variants wtRebH, wt+T, wt+L, 5LS, 6T and 6L. I52T does not increase selectivity for 5/6-halogenation in wtRebH. While F465L does increase the selectivity for 5/6-halogenation, the selectivity still remains low.

To test whether mutations found in Rounds 0-5 were necessary for 10S and 8F activity and selectivity, the mutations at residues 52 and 465 were introduced into wtRebH (I52H + F465C from 10S; I52M + F465L from 8F), thus forming wtHC and wtML. 50 mL expression cultures of wtRebH, wtHC, wtML, 10S, and 8F were grown, lysed and purified as described in the General Procedures. Reactions were conducted in duplicate and analyzed according to the procedure found in "Conversion and selectivity determination of lineage" (5% RebH loading). Results are shown in Figure S22 (no 7-halogenation was observed for wtHC, wtML, 10S or 8F, so it is not included in Fig. S22).

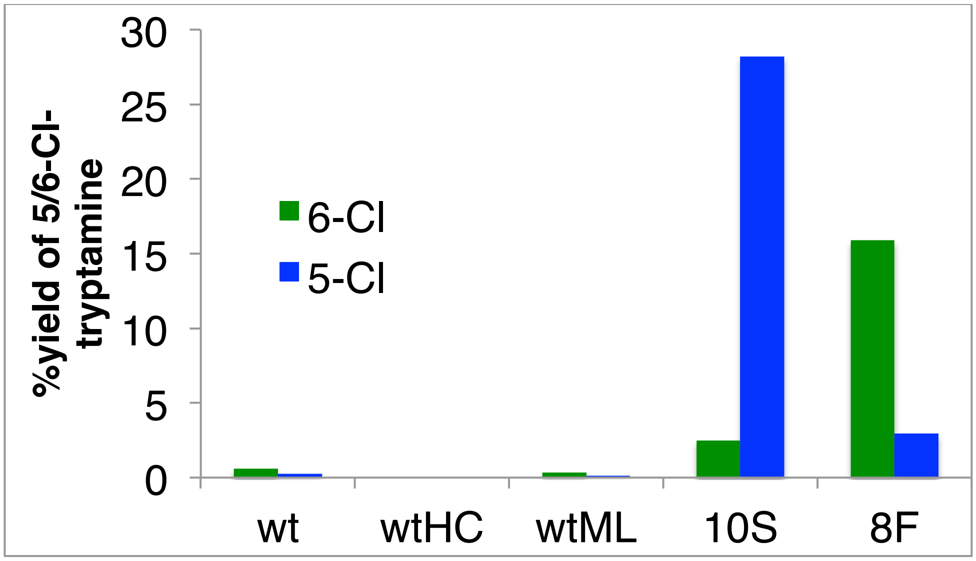

Figure S22: Yield of 5 and 6 chlorotryptamines for variants wtRebH, wtHC, wtML, 10S and 8F. No activity is observed for wtHC. Less than 0.5% total conversion is observed for wtML.

Halenium Affinity Calculations: Halenium affinity (HalA) refers to the energy associated with the reaction of a substrate with a halenium ion (X^+). In this study, X = Cl, and the reaction involves attack of Cl^+ by different sites on tryptamine. A representative reaction (reaction of Cl^+ at the 2-position of tryptamine) is shown in eq. 1. Please note, this is NOT meant to imply that RebH generates "Cl^+". The calculated HalA simply provides a relative measure of substrate reactivity toward electrophilic chlorenium ion donors, such as HOX or chloramine.

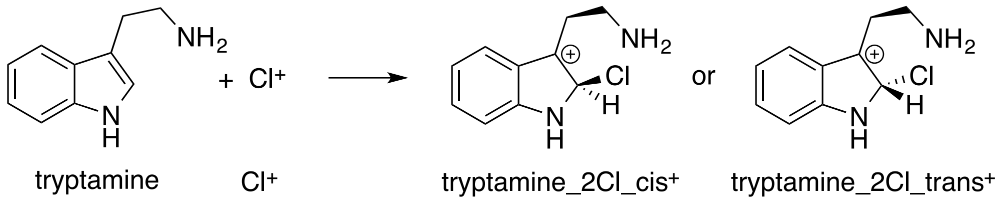

(1)

Geometry optimizations and frequency calculations were conducted using Gaussian (DFT, B3LYP, LANL2DZ). Different conformations of the aminoethyl group led to diastereomeric product structures (eq. 1), so energies for both were used to calculate HalA values. A significant difference in these values was only observed for 2-chlorination (see below). Halenium affinity values for different positions on tryptamine were calculated using equations

(2) and (3) according a literature procedure.[18] Electronic energies ($E_{(elec)}$), zero point energies (*ZPE*), and frequencies (ν) were obtained directly from Gaussian output files (Sum of electronic and zero-point Energies, Zero-point correction, and Vibrational temperatures, respectively).

$$HalA = -\Delta E_{(elec)} - \Delta ZPE - \Delta E'_{(vib)} + \frac{5}{2}RT \quad (2)$$

$$E'_{(vib)}(T) = \sum_{i=1}^{3n-6} \frac{Nhv_i}{e^{Nhv_i} - 1} \quad (3)$$

Table 1. Complete HalA calculation summary (using eq. 2):

1	A	B	C	D	E	F	G	H	I	J	K	L	M
2	Species	**Electronic Energy (E) (hartree)**	**Zero Point Energy (ZPE) (hartree)**	**E'vib(T) (J/mol)**	**5/2RT**	**ΔE (hartrees)**	**ΔE (J/mol)**	**ΔZPE (hartrees)**	**ΔZPE (J/mol)**	**ΔEv(T)**	**5/2RT**	**HalA (J/mol)**	**HalA (kcal/mol)**
**3**	**tryptamine**	-497.51	0.21	19704.45									
**4**	**Cl^+**	-14.41	0.00	0.00									
**5**	**tryptamine+Cl^+**	-511.92	0.21	19704.45									
**6**	**tryptamine_2Cl_cis$^+$**	-512.20	0.21	23723.54	6197.39	-0.28	-722700.38	0.00	6760.66	4019.09	6197.39	726156.20	173.44
**7**	**tryptamine_2Cl_trans$^+$**	-512.20	0.21	23478.41	6197.39	-0.28	-737917.78	0.00	7632.33	3773.96	6197.39	740256.80	176.81
**8**	**tryptamine_4Cl_cis$^+$**	-512.19	0.21	23586.32	6197.39	-0.26	-693457.56	0.00	8223.07	3881.86	6197.39	695313.75	166.07
**9**	**tryptamine_4Cl_trans$^+$**	-512.19	0.21	23478.41	6197.39	-0.26	-692987.60	0.00	7800.36	3773.96	6197.39	695158.59	166.04
**10**	**tryptamine_5Cl_cis$^+$**	-512.18	0.21	23497.31	6197.39	-0.26	-678500.09	0.00	7356.65	3792.86	6197.39	681133.69	162.69
**11**	**tryptamine_5Cl_trans$^+$**	-512.18	0.21	23473.47	6197.39	-0.26	-678426.57	0.00	7435.42	3769.01	6197.39	680957.56	162.64
**12**	**tryptamine_6Cl_cis$^+$**	-512.19	0.21	23484.29	6197.39	-0.26	-692730.30	0.00	8204.69	3779.83	6197.39	694502.84	165.88
**13**	**tryptamine_6Cl_trans$^+$**	-512.19	0.21	23594.07	6197.39	-0.26	-692677.79	0.00	7931.64	3889.62	6197.39	694833.16	165.96
**14**	**tryptamine_7Cl_cis$^+$**	-512.18	0.21	23767.16	6197.39	-0.26	-670985.91	0.00	6999.58	4062.70	6197.39	674246.42	161.04
**15**	**tryptamine_7Cl_trans$^+$**	-512.18	0.21	23777.05	6197.39	-0.26	-670618.34	0.00	6986.46	4072.59	6197.39	673901.87	160.96
**16**	**tryptamine_NCl$^+$**	-512.17	0.21	22654.48	6197.39	-0.25	-658848.22	0.00	11226.64	2950.03	6197.39	656769.01	156.87

Table 2. Representative E'vib(T) calculation data (using eq. 3):

temp (K)	v	Nhv	Nhv/RT	e(Nhv/RT)	e(Nhv/RT)-1	Nhv/(e(Nhv/RT)-1)
43.8	9.12644E+11	364.1734452	0.146905922	1.158244992	0.158244992	2301.326819
343.6	7.15946E+12	2856.849219	1.152440064	3.16590851	2.16590851	1319.007338
694.86	1.44785E+13	5777.387218	2.330571893	10.2838211	9.2838211	622.3070388
925.17	1.92774E+13	7692.291012	3.103035429	22.26543396	21.26543396	361.7274411
1140.12	2.37562E+13	9479.484666	3.823981272	45.78613301	44.78613301	211.6611556
1369.77	2.85414E+13	11388.90091	4.594231158	98.91205854	97.91205854	116.3176537
1526.08	3.17983E+13	12688.5345	5.118497474	167.0841327	166.0841327	76.39823442
1723.19	3.59055E+13	14327.39815	5.779607663	323.6321927	322.6321927	44.40783802
1899.49	3.95789E+13	15793.23784	6.370920769	584.5958589	583.5958589	27.06194295
2091.61	4.35821E+13	17390.61232	7.015294416	1113.53444	1112.53444	15.63152717
2301.47	4.79549E+13	19135.48536	7.719168315	2251.086607	2250.086607	8.504332815
4315.94	8.99296E+13	35884.72007	14.4757339	1935224.501	1935223.501	0.018542933
4611.27	9.60833E+13	38340.2302	15.46627559	5210964.842	5210963.842	0.007357608
5316.51	1.10778E+14	44203.92153	17.83166217	55487158.38	55487157.38	0.000796651
177.01	3.68829E+12	1471.742957	0.593694458	1.810665501	0.810665501	1815.475009
392.96	8.18796E+12	3267.251074	1.317994317	3.735920785	2.735920785	1194.20529
744.86	1.55204E+13	6193.110329	2.498272717	12.16146951	11.16146951	554.8651386
971.65	2.02459E+13	8078.747216	3.258930116	26.02168205	25.02168205	322.8698694
1258.36	2.622E+13	10462.58668	4.220560181	68.07160606	67.07160606	155.9912949
1398.64	2.91429E+13	11628.93944	4.691061614	108.9688012	107.9688012	107.7064792
1592.15	3.3175E+13	13237.87102	5.340097343	208.5330086	207.5330086	63.78682174
1802.55	3.7559E+13	14987.23387	6.045782411	422.3280625	421.3280625	35.57141147

1949.66	4.06243E+13	16210.37441	6.539191776	691.7272815	690.7272815	23.46855966
2140.02	4.45908E+13	17793.11544	7.177662354	1309.842728	1308.842728	13.59454048
2338.29	4.87221E+13	19441.62386	7.842663202	2546.978928	2545.978928	7.636207687
4406.58	9.18182E+13	36638.34293	14.77974195	2622771.007	2622770.007	0.013969331
4637.59	9.66317E+13	38559.06684	15.5545533	5691892.21	5691891.21	0.006774386
188.36	3.92479E+12	1566.112104	0.631762545	1.88092287	0.88092287	1777.808429
487.8	1.01641E+13	4055.794671	1.63608924	5.135048254	4.135048254	980.8336981
813.71	1.6955E+13	6765.561053	2.729196752	15.32057586	14.32057586	472.4363824
1020.3	2.12596E+13	8483.245803	3.422103017	30.63377068	29.63377068	286.2695367
1277.59	2.66207E+13	10622.47379	4.285057918	72.60675186	71.60675186	148.3445836
1456.49	3.03483E+13	12109.93108	4.885091467	132.3025661	131.3025661	92.2292034
1617.9	3.37116E+13	13451.96842	5.426463268	227.3437678	226.3437678	59.431583
1817.72	3.78751E+13	15113.36427	6.096662841	444.3723518	443.3723518	34.08729526
2005.26	4.17828E+13	16672.65851	6.725675092	833.5344995	832.5344995	20.02638752
2174.26	4.53042E+13	18077.80263	7.292503878	1469.244906	1468.244906	12.31252535
2399.88	5.00054E+13	19953.71159	8.049237077	3131.405039	3130.405039	6.374162878
4446.02	9.264E+13	36966.26532	14.91202436	2993711.151	2993710.151	0.012347977
4757.01	9.912E+13	39551.97992	15.95508995	8495863.443	8495862.443	0.00465544
260.8	5.43419E+12	2168.411747	0.874727499	2.398221686	1.398221686	1550.835442
600.65	1.25155E+13	4994.081732	2.01459	7.497652717	6.497652717	768.5978229
845.27	1.76126E+13	7027.96548	2.835049512	17.03124354	16.03124354	438.3917856
1112.45	2.31797E+13	9249.423496	3.731175636	41.72813645	40.72813645	227.1015642
1281.77	2.67078E+13	10657.22824	4.299077707	73.63185225	72.63185225	146.7294019
1465.35	3.05329E+13	12183.59721	4.914808053	136.2931463	135.2931463	90.05332162
1662.28	3.46363E+13	13820.96426	5.575314519	263.832523	262.832523	52.58468055
1861.1	3.8779E+13	15474.04564	6.242160076	513.9675218	512.9675218	30.16574145
2035.87	4.24206E+13	16927.1642	6.828341537	923.6576894	922.6576894	18.34609346
2184.84	4.55247E+13	18165.76964	7.327989372	1522.317879	1521.317879	11.94081125
2419.61	5.04165E+13	20117.75593	8.115411822	3345.635107	3344.635107	6.014932956
4579.18	9.54146E+13	38073.41911	15.3586452	4679235.024	4679234.024	0.008136678
5064.84	1.05534E+14	42111.42083	16.98755684	23856251.14	23856250.14	0.001765215
323.75	6.74586E+12	2691.807144	1.085862836	2.961994433	1.961994433	1371.974914
631.14	1.31508E+13	5247.589685	2.116853963	8.304968607	7.304968607	718.3589647
856.12	1.78386E+13	7118.177395	2.871440591	17.66244425	16.66244425	427.1988725
1118.98	2.33158E+13	9303.716934	3.753077364	42.6521364	41.6521364	223.3671005
1298.93	2.70653E+13	10799.90441	4.35663263	77.99405689	76.99405689	140.2693253
1486.41	3.09718E+13	12358.69979	4.98544364	146.2684512	145.2684512	85.07490569
1676.37	3.49299E+13	13938.11503	5.622572611	276.6000532	275.6000532	50.57370226
1877.69	3.91247E+13	15611.98256	6.29780321	543.3769129	542.3769129	28.78437889
2059.78	4.29188E+13	17125.96299	6.908536071	1000.781097	999.781097	17.12971274
2207.56	4.59981E+13	18354.67422	7.404192627	1642.857901	1641.857901	11.17920997
4252.08	8.8599E+13	35353.75851	14.26154641	1562107.465	1562106.465	0.022632106
4590.7	9.56547E+13	38169.20171	15.39728347	4863570.846	4863569.846	0.007847981
5240.38	1.09192E+14	43570.94153	17.5763209	42983242.85	42983241.85	0.001013673
						19704.45426

In silico mutation and molecular docking studies: The holo form of RebH structure (resolution of 2.15Å, PDB ID: 2OA1) was used for molecular simulations[19]. FAD, Cl^- and Trp were removed for obtaining receptor structure. Using Swiss-PDBViewer, the 8F, 10S, and 0S variants were modulated for each multiple mutation sites[20]. Furthermore, energy minimization was proceed for repair distorted geometry (wild type structure was also minimized for further simulation). The Swiss-PDBViewer uses GROMOS 43B1 force field for minimization. Molecular docking simulation was carried out with AutoDock Vina, which has been successfully used for detecting binding poses, was used for molecular docking[21]. The target ligand, tryptamine, was set to rotate three rotatable bonds in the aminoethyl substituent. All protein

structures including ligand were converted into PDBQT format by AutoDock tools to run the simulation. Polar hydrogen bonds were added to receptors (protein structure) and Gasteiger charges were typed to the structures. A cubic grid box (grid spacing = 1.000 Å; 24 × 20 × 20 grid points) was centered on the coordinates of the tryptophan in original PDB. Exhaustiveness and number of binding modes were set to 15. Obtained docking poses were analyzed based on binding energies and the mechanism of halogenase that could ensure feasibility of each reactive position.

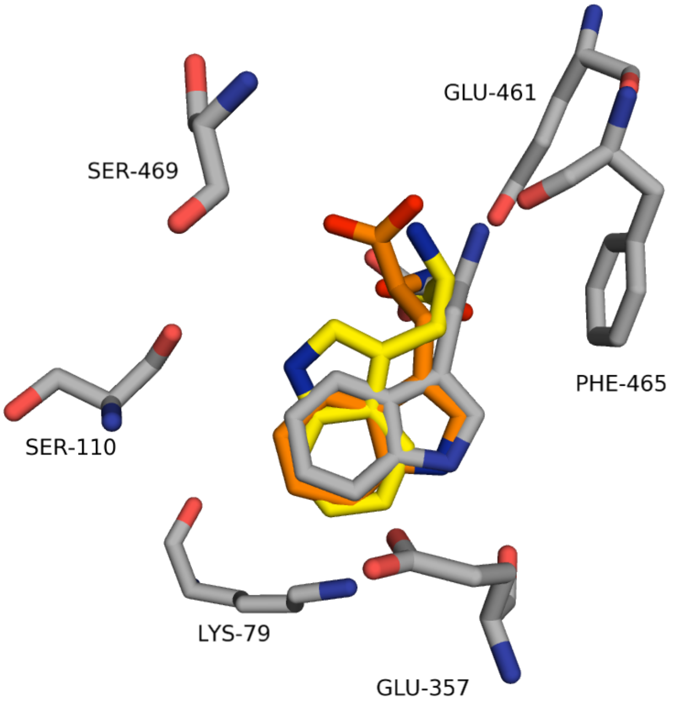

Fig S23. Simulated tryptophan binding poses of RebH showing 7-selectivity (orange) and 5-selectivity (yellow) comparing with crystal structure (grey).

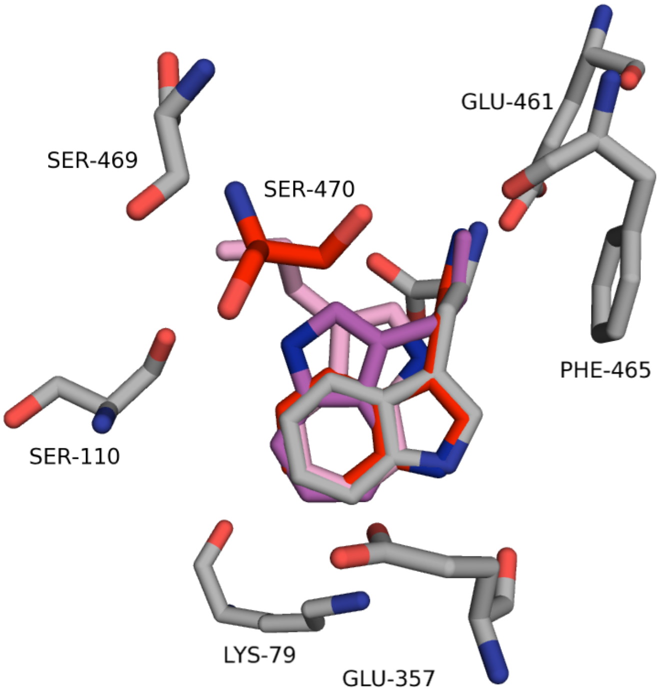

Fig S24. Tryptamine binding poses of 0S showing 7-selectivity (red), 5-selectivity (purple) and 6-selectivity (pink) comparing with crystal structure (grey).

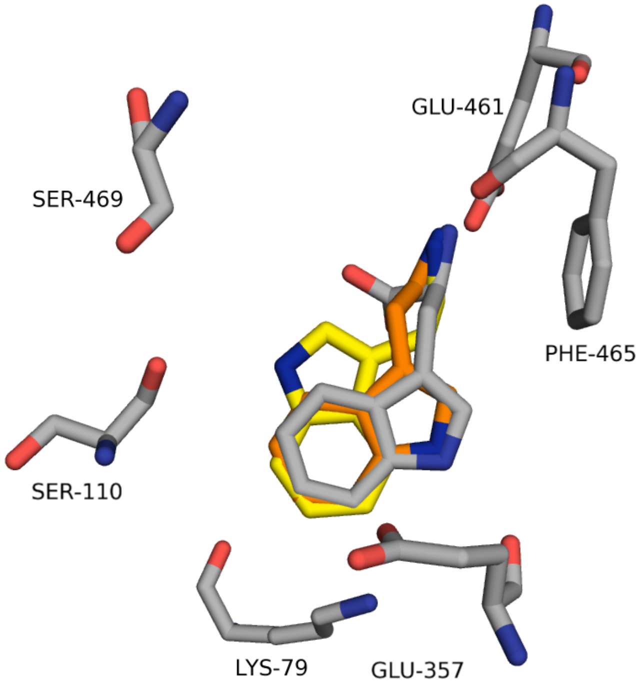

Fig S25. Tryptamine binding poses of RebH showing 7-selectivity (orange) and 5-selectivity (yellow) comparing with crystal structure (grey).

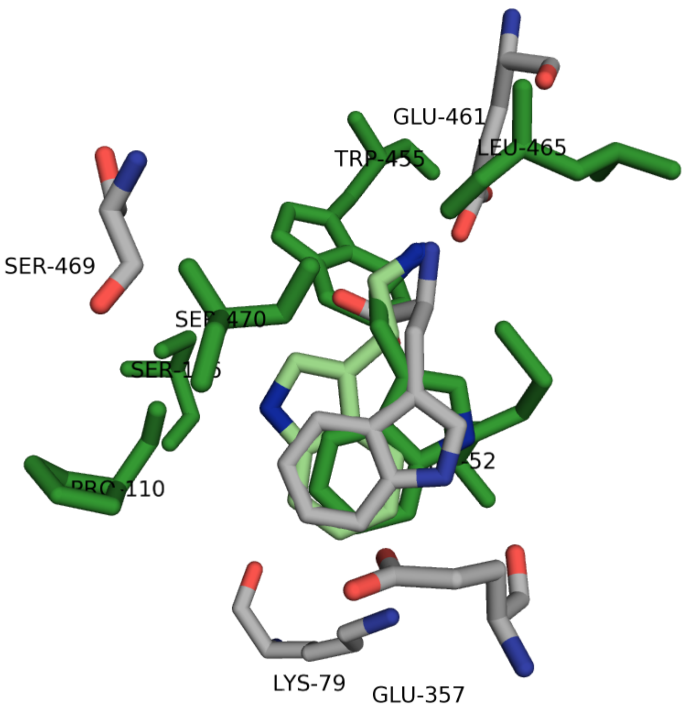

Fig S26. Tryptamine binding poses of 8F showing 6-selectivity (green) and flipped pose (lime) comparing with crystal structure (grey).

Activity of 0S, 8F, and 10S on Tryptophan: RebH variants (0.01-0.25 equiv., 5-50 μM final concentration), MBP-RebF (0.005 equiv., 2.5 μM final concentration), and glucose dehydrogenase (9 U/mL final concentration) were added as solutions (25 mM HEPES, pH 7.4) to a 96-well plate. A solution containing tryptamine and/or L-tryptophan (1 equiv., 0.5 mM final concentration), FAD (0.2 equiv., 100 μM final concentration), NAD (0.2 equiv., 100 μM final concentration), NaCl (20 equiv., 10 mM final concentration), and glucose (40 equiv., 20 mM final concentration) was added to these tubes to initiate reaction. Final reaction volume was 75 μL. Reactions were agitated for 2 or 16 hours at 650 rpm at room temperature. These were quenched with 75 μL of methanol, centrifuged to remove precipitated protein, filtered, and analyzed by UPLC Method 1, as described in the General Procedures. From these reactions, it was found that only 0S chlorinates L-tryptophan.

A.

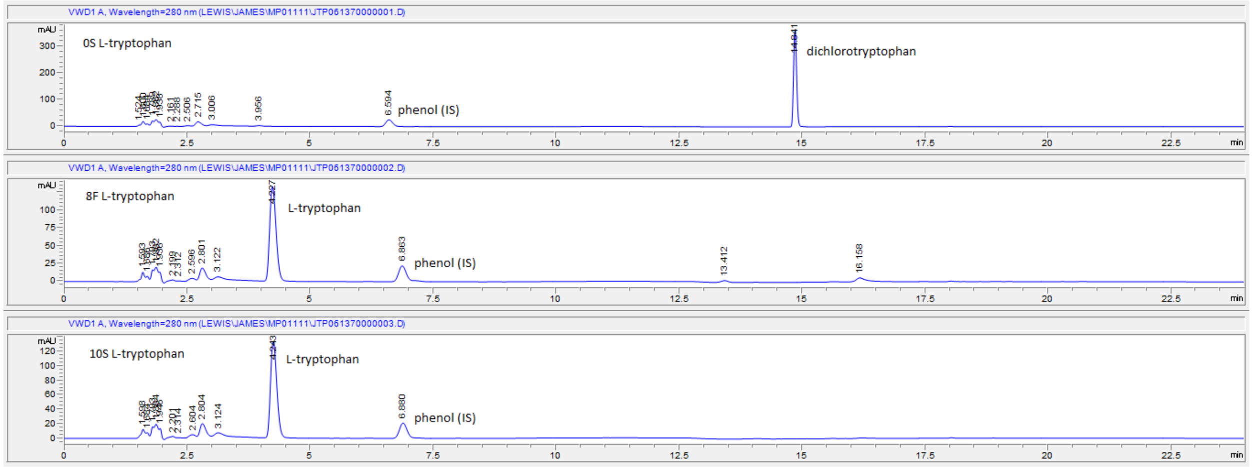

B.

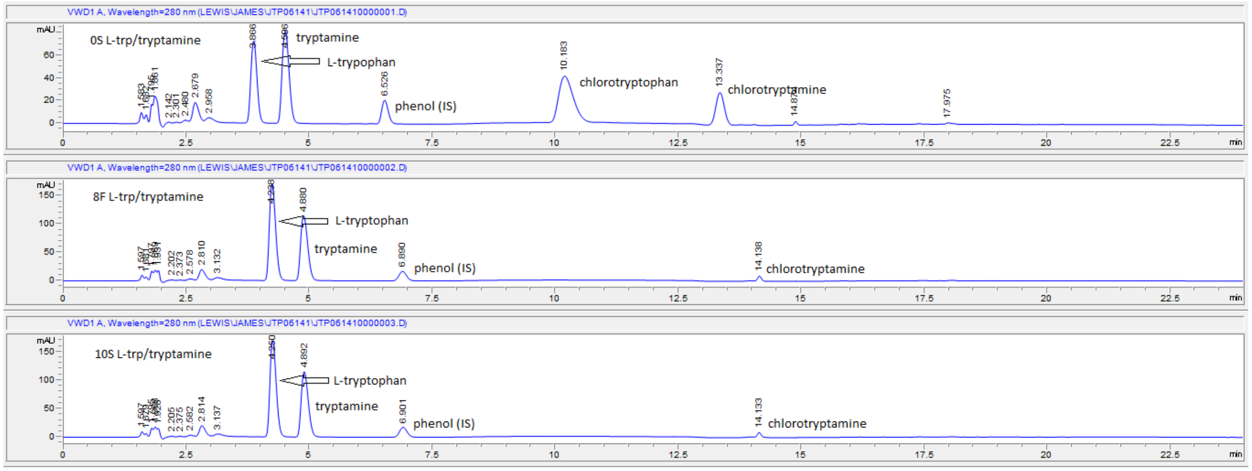

C.

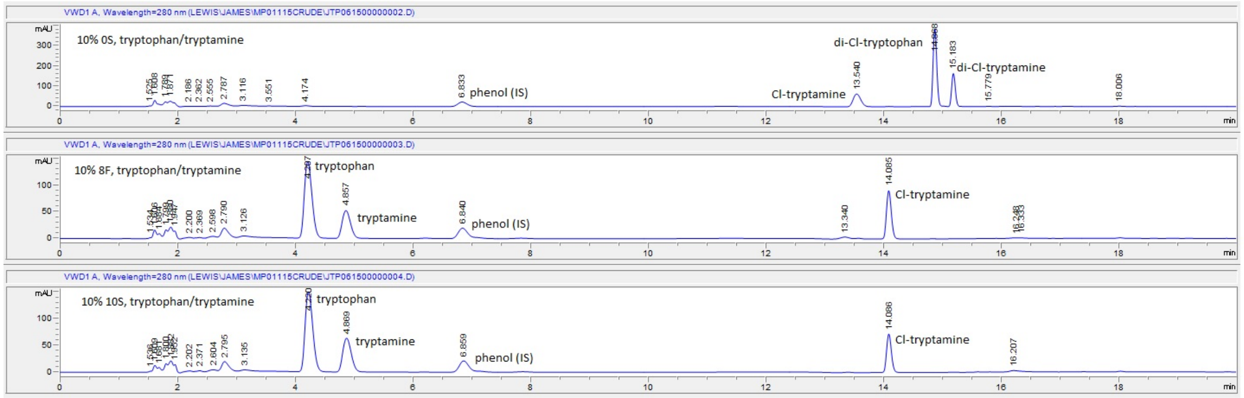

Fig. S27. UPLC traces showing conversion of L-tryptophan and/or tryptamine by RebH variants. A. Reactions were conducted for 16 hours, with 25 uM RebH and 0.5 mM L-tryptophan. B. Reactions were conducted for 2 hours, with 5 uM RebH, 0.5 mM L-tryptophan and 0.5 mM tryptamine. C. Reactions were conducted for 16 hours, with 50 uM RebH, 0.5 mM L-tryptophan and 0.5 mM tryptamine.

Additional Substrate Scope Profile for wtRebH and 10S: In addition to the tryptamine derivatives described in the substrate scope section of this work, other substrates were found to be accepted for halogenation by several variants, but were not isolated or fully characterized. A representative sample of these substrates is shown below (Fig. S28). These reactions were conducted according to the procedure outlined in the General Procedures, using 25 μM RebH and 0.5 mM final substrate concentration. UHPLC Method 1 was used to determine conversions. While 10S does not entirely alter selectivity of halogenation compared to wtRebH, it does significantly alter it in most cases. Using the MALDI-MS method described in this work, variants with altered selectivity could be further evolved for higher selectivity. In some cases, for example 2,3-dimethylindole, selectivity is dramatically altered; however, this is accompanied by significant decreases in activity. As discussed in more detail below, this can be readily overcome through subsequent rounds of evolution with a focus on enzyme activity.

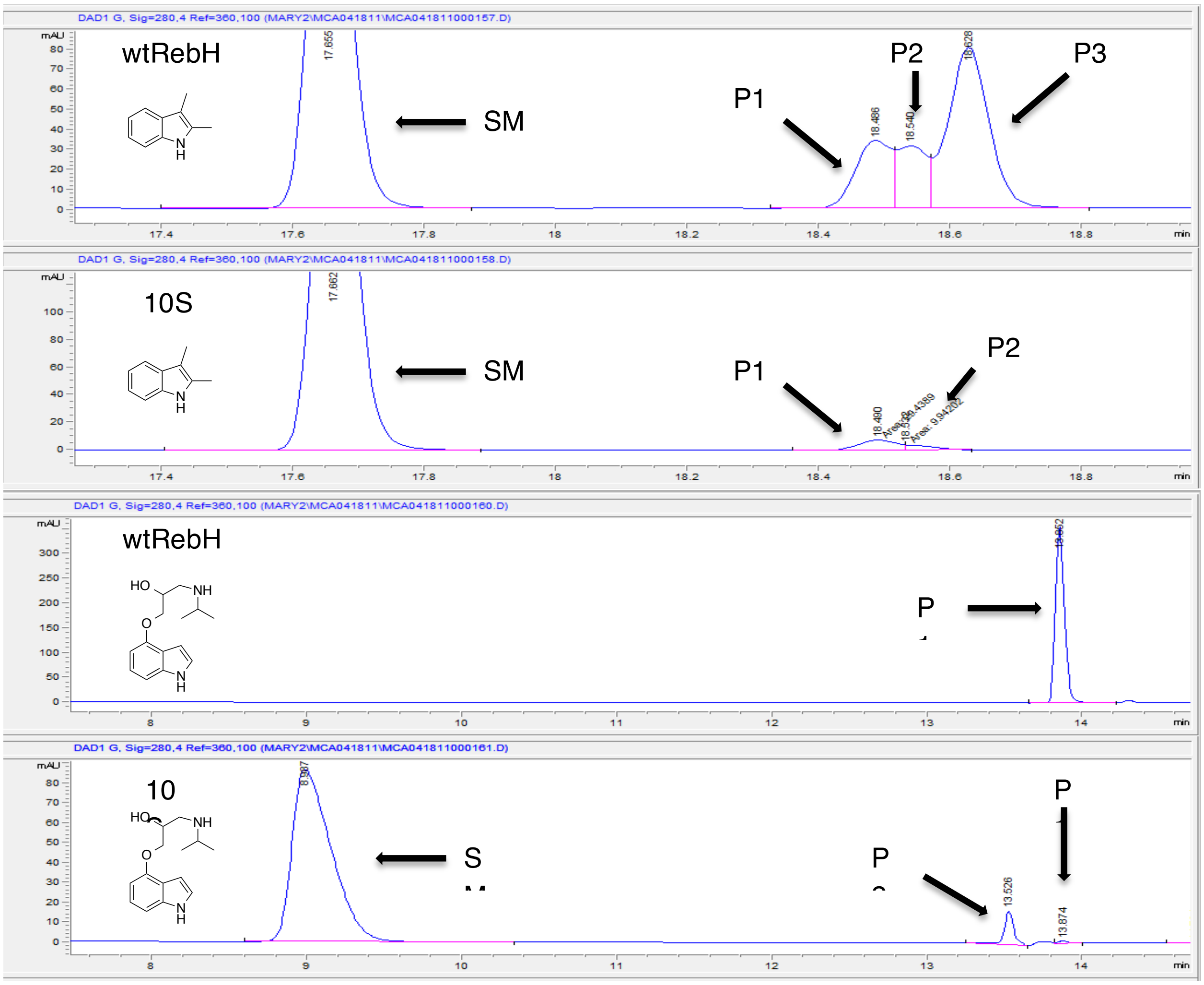

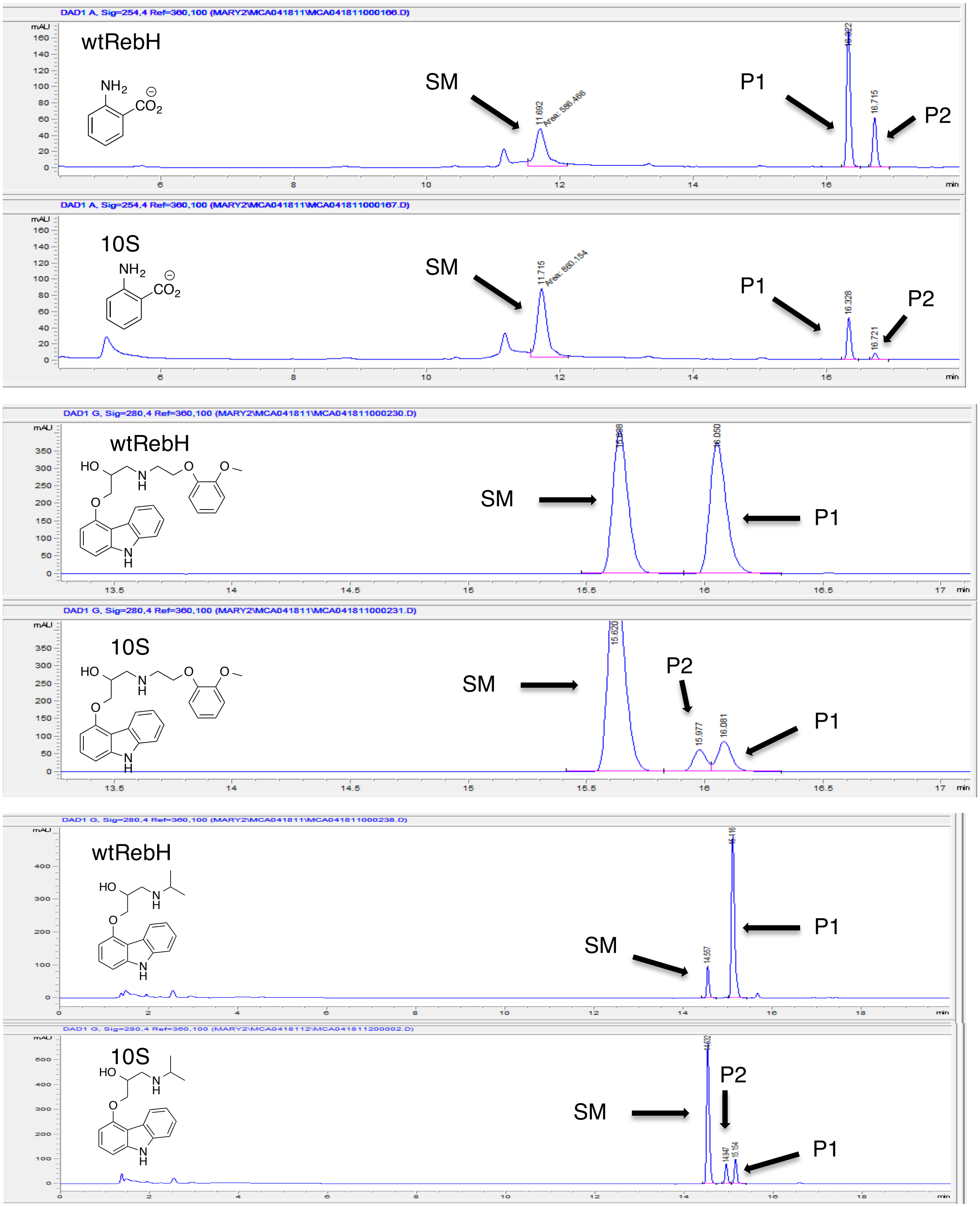

Fig. S28. UPLC traces showing additional scope and selectivity of wtRebH compared to the evolved 5-tryptamine halogenase, 10S.

Increasing Enzyme Activity through Directed Evolution

Like has been shown for many other enzymes, halogenase activity can be increased through directed evolution. This has been demonstrated in previous reports[10] as well as in this work (Fig. S29). Throughout this evolution, increases in conversion (1.7-11 fold) were found without a decrease in selectivity (Fig. S29). Because the focus of this work was to develop variants that had significantly altered selectivity, 10S and 8F were not further improved for activity in this study. However, these enzymes could be further evolved using the MALDI-MS assay described here to directly screen for highly selective mutants with increased activity.

	15 uM enzyme				25 uM enzyme											
	WT	0S	1P	2RFQ	3W	4PL	5LS	6T	6L	6TL	6S	7M	8F	7H	8C	10S
%yield 7	19.6	48.5	23.2	38.6	70.3	38.8	55.3	35.6	6.8	5.8	8.0	0.0	0.0	0.0	0.0	0.9
%yield 6	0.2	0.3	0.8	1.0	2.8	3.0	5.0	6.6	3.6	7.8	9.1	2.0	22.3	0.3	0.8	2.6
%yield 5	0.1	0.2	0.5	0.7	2.5	2.7	3.7	4.6	2.6	8.7	2.3	0.8	3.8	0.9	5.0	23.5
% sel for major isomer	0.99	0.99	0.95	0.96								0.71	0.85		0.86	0.87
fold increase in yield		2.5		1.67									11			4.7

Fig. 29. Increases in conversion along the evolution lineage that occurred without a decrease in selectivity for the desired isomer are boxed. Observed increases in conversion ranged from 1.7-11 fold.

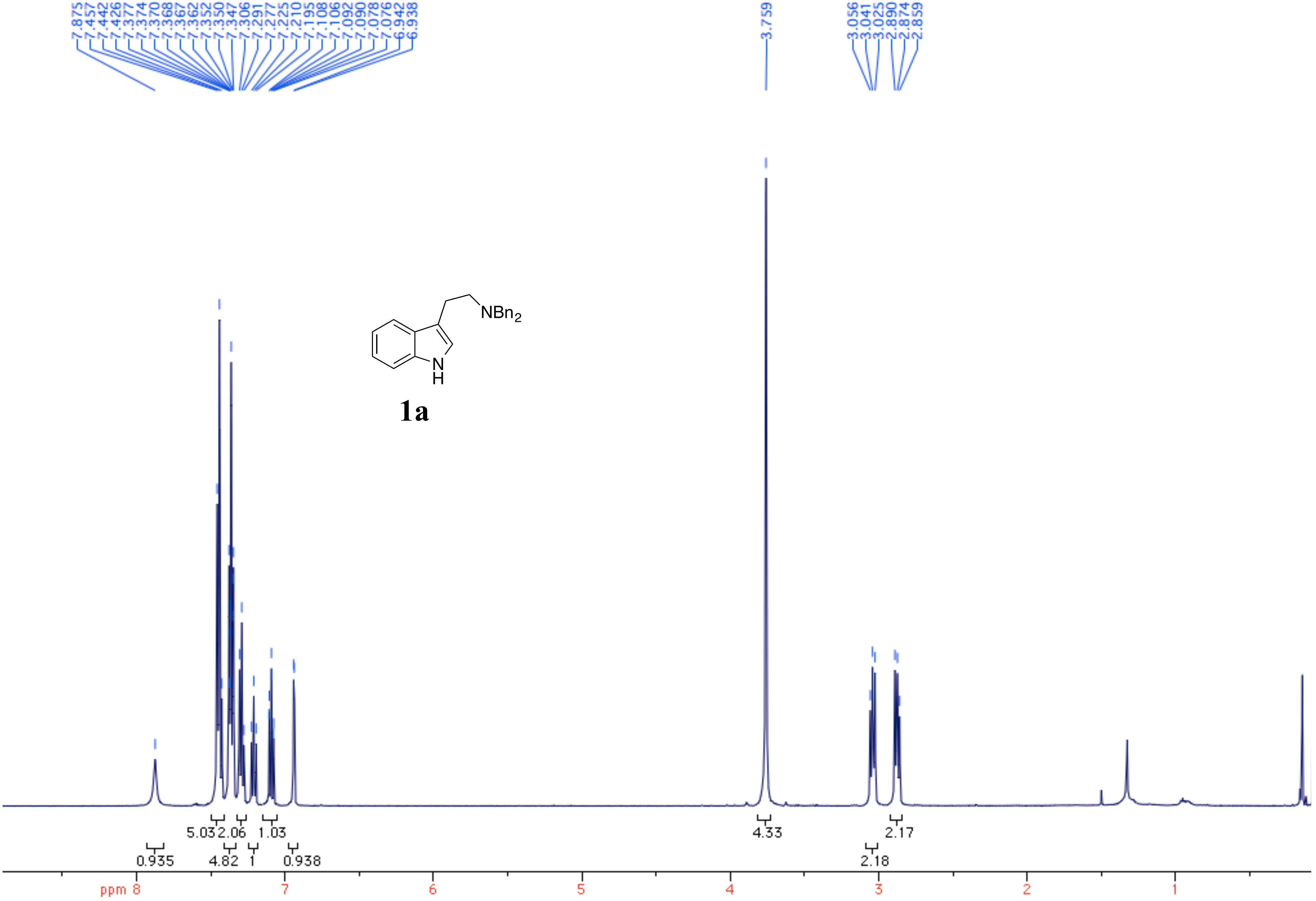
7.875
7.457
7.442
7.426
7.377
7.374
7.370
7.368
7.367
7.362
7.352
7.350
7.347
7.306
7.291
7.277
7.225
7.210
7.195
7.108
7.106
7.092
7.090
7.078
7.076
6.942
6.938
3.759
3.056
3.041
3.025
2.890
2.874
2.859
NBn2
N
H
1a
0.935
5.03
4.82
2.06
1
1.03
0.938
4.33
2.18
2.17
ppm 8
7
6
5
4
3
2
1

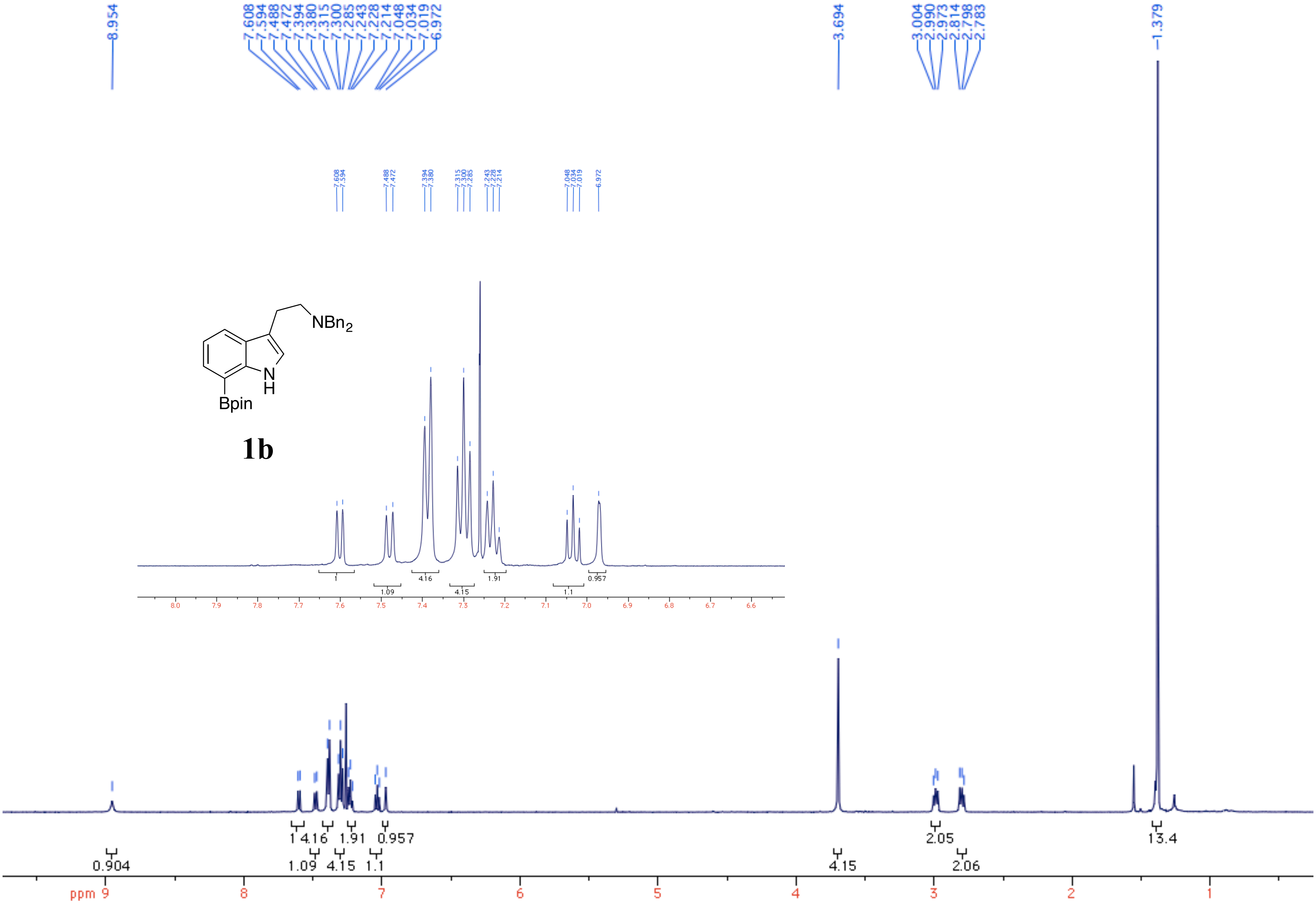

8.954
7.608
7.594
7.488
7.472
7.394
7.380
7.315
7.300
7.285
7.243
7.228
7.214
7.048
7.034
7.019
6.972
3.694
3.004
2.990
2.973
2.814
2.798
2.783
1.379
NBn2
N
H
Bpin
1b
1
1.09
4.16
4.15
1.91
1.1
0.957
0.904
2.05
2.06
13.4
ppm

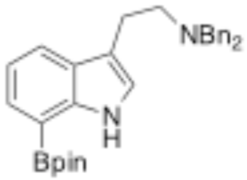
NBn2
N
H
Bpin

1b

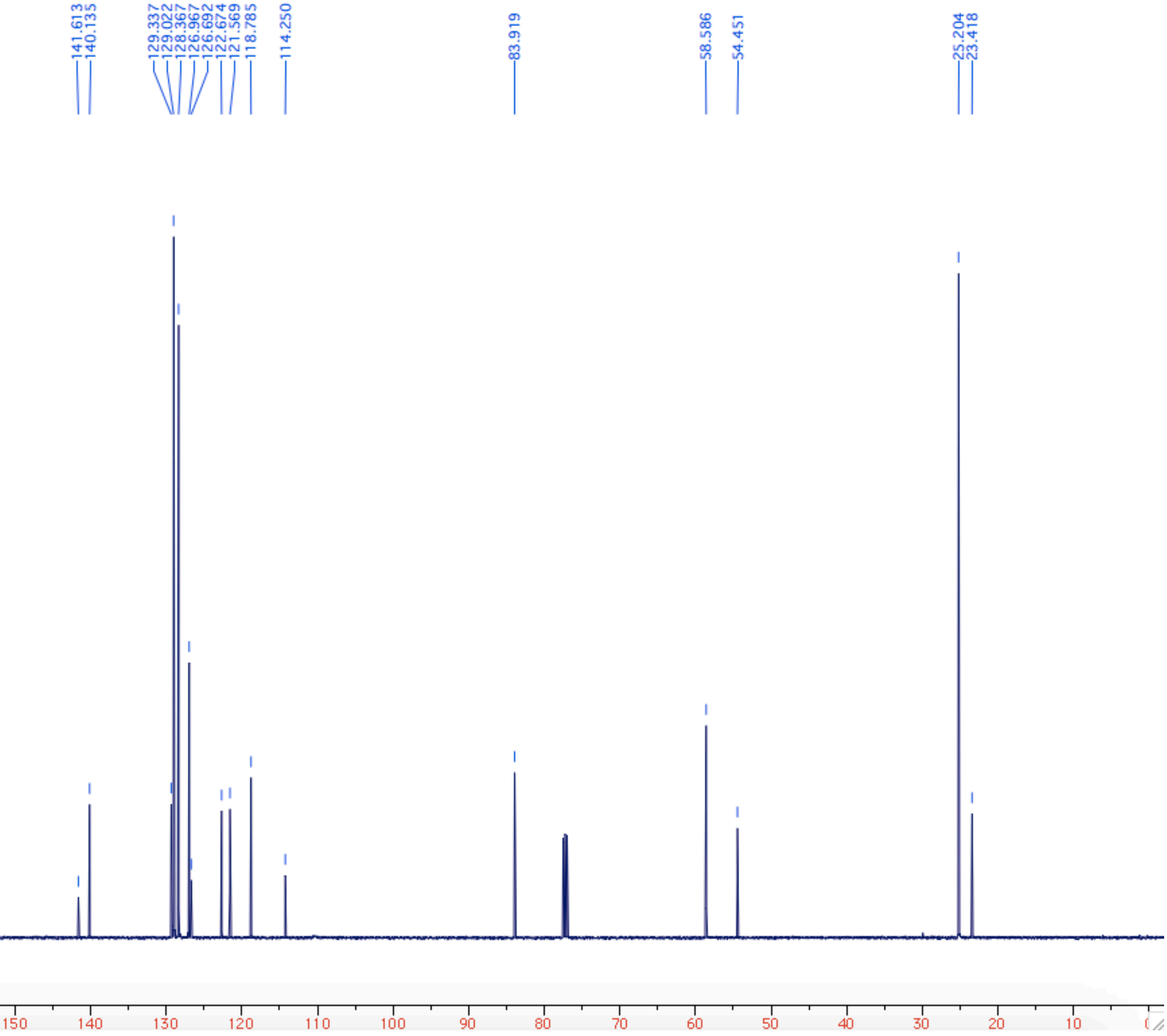
141.613
140.135
129.337
129.022
128.367
126.967
126.692
122.674
121.569
118.785
114.250
83.919
58.586
54.451
25.204
23.418
180
170
160
150
140
130
120
110
100
90
80
70
60
50
40
30
20
10

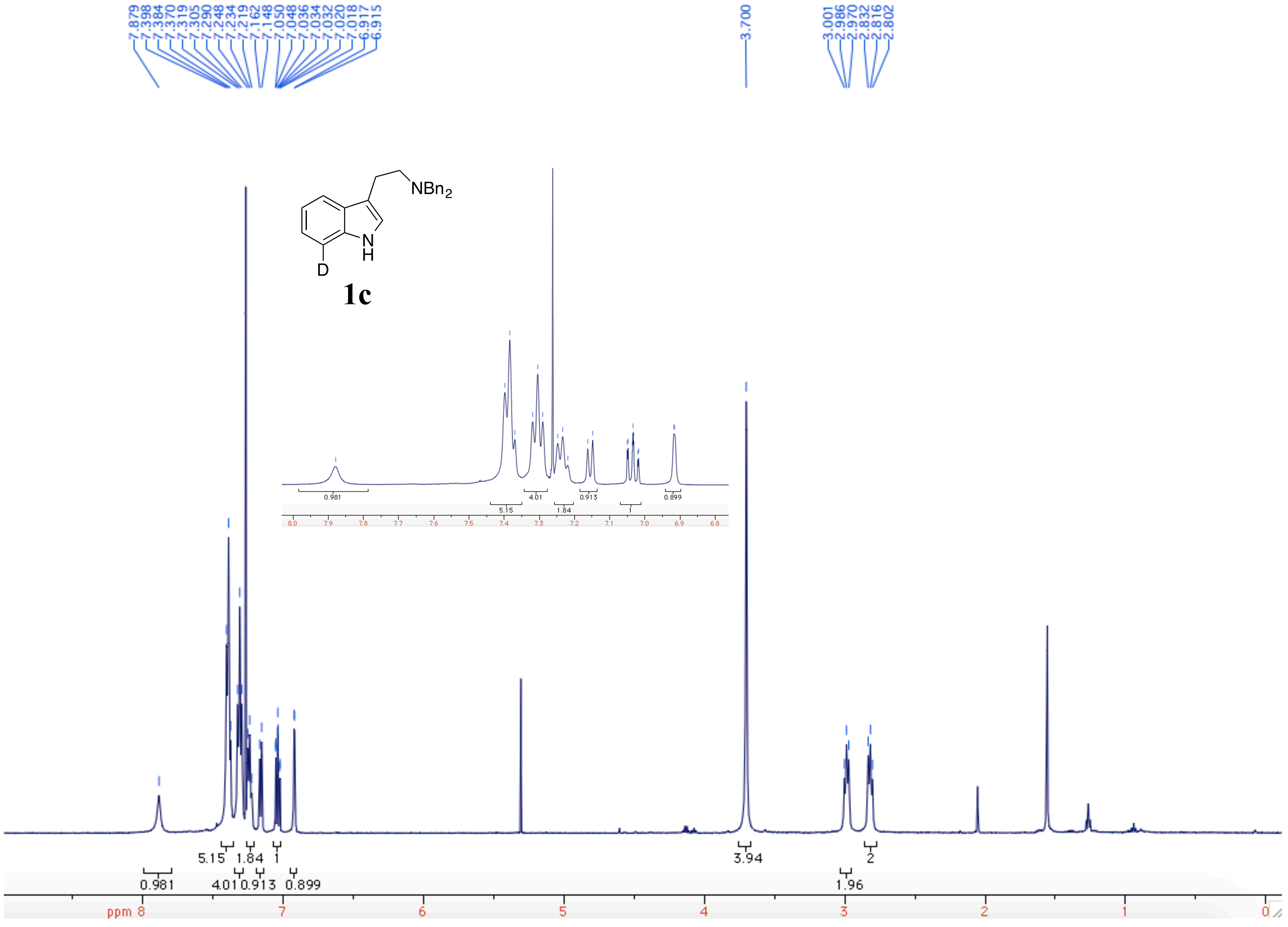

7.879
7.398
7.384
7.370
7.319
7.305
7.290
7.248
7.234
7.219
7.162
7.148
7.050
7.048
7.036
7.034
7.032
7.020
7.018
6.917
6.915
3.700
3.001
2.986
2.970
2.832
2.816
2.802
NBn2
N
H
D
1c
0.981
5.15
4.01
1.84
0.913
1
0.899
3.94
1.96
2
ppm

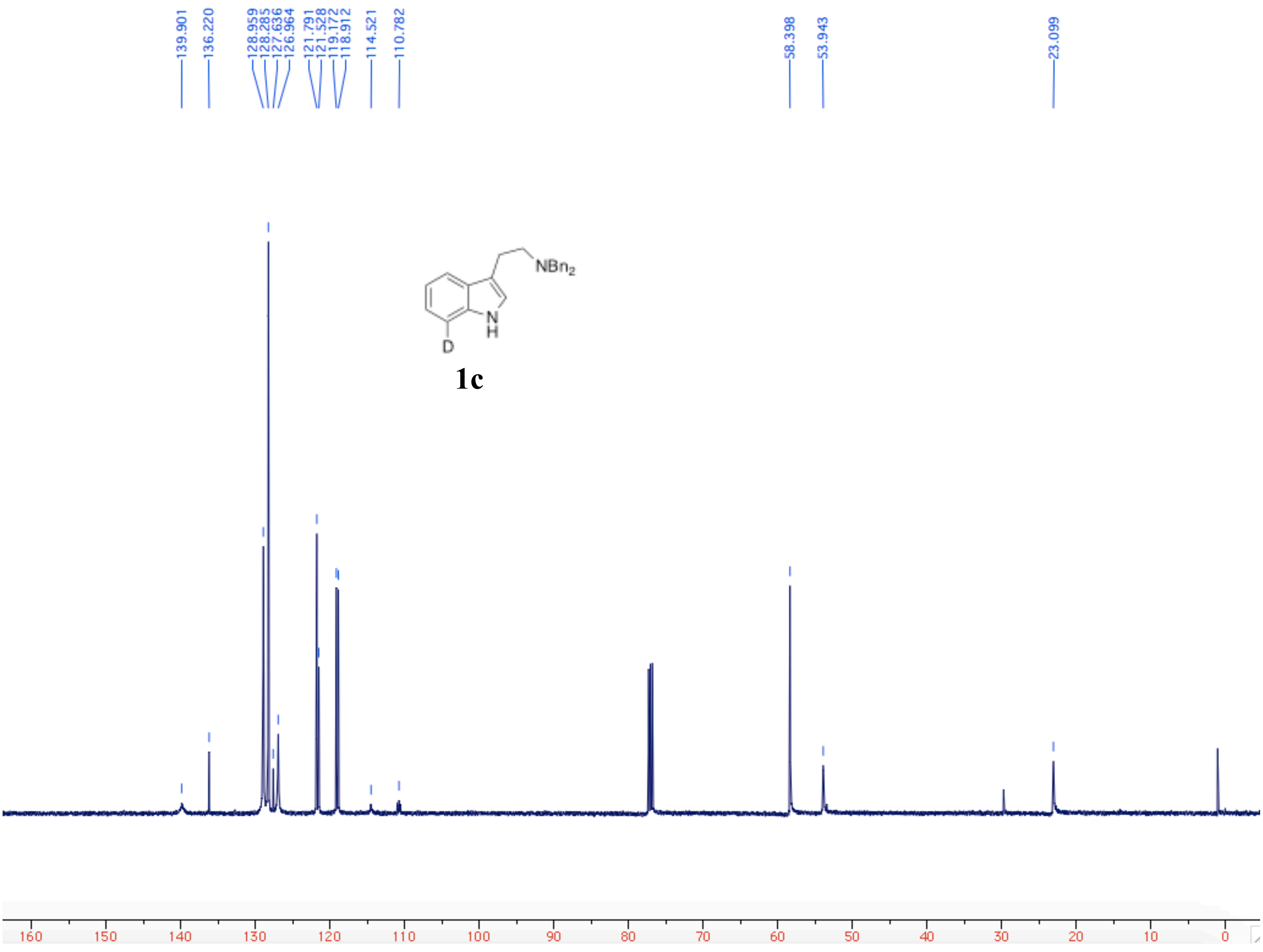
139.901
136.220
128.959
128.285
127.636
126.964
121.791
121.528
119.172
118.912
114.521
110.782
58.398
53.943
23.099
NBn2
N
H
D
1c
160
150
140
130
120
110
100
90
80
70
60
50
40
30
20
10
0

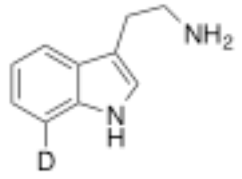

NH2
N
H
D

Probe 1

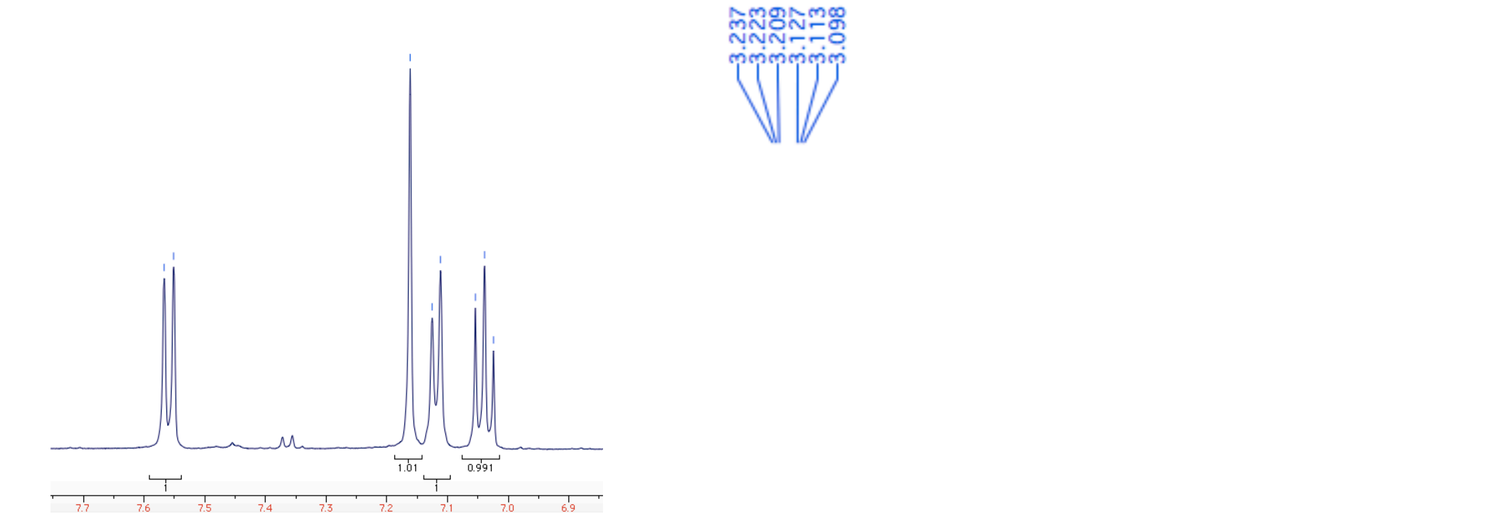

7.567
7.551
7.162
7.125
7.112
7.054
7.039
7.024
3.237
3.223
3.209
3.127
3.113
3.098
1.01
1
0.991
7.7
7.6
7.5
7.4
7.3
7.2
7.1
7.0
6.9

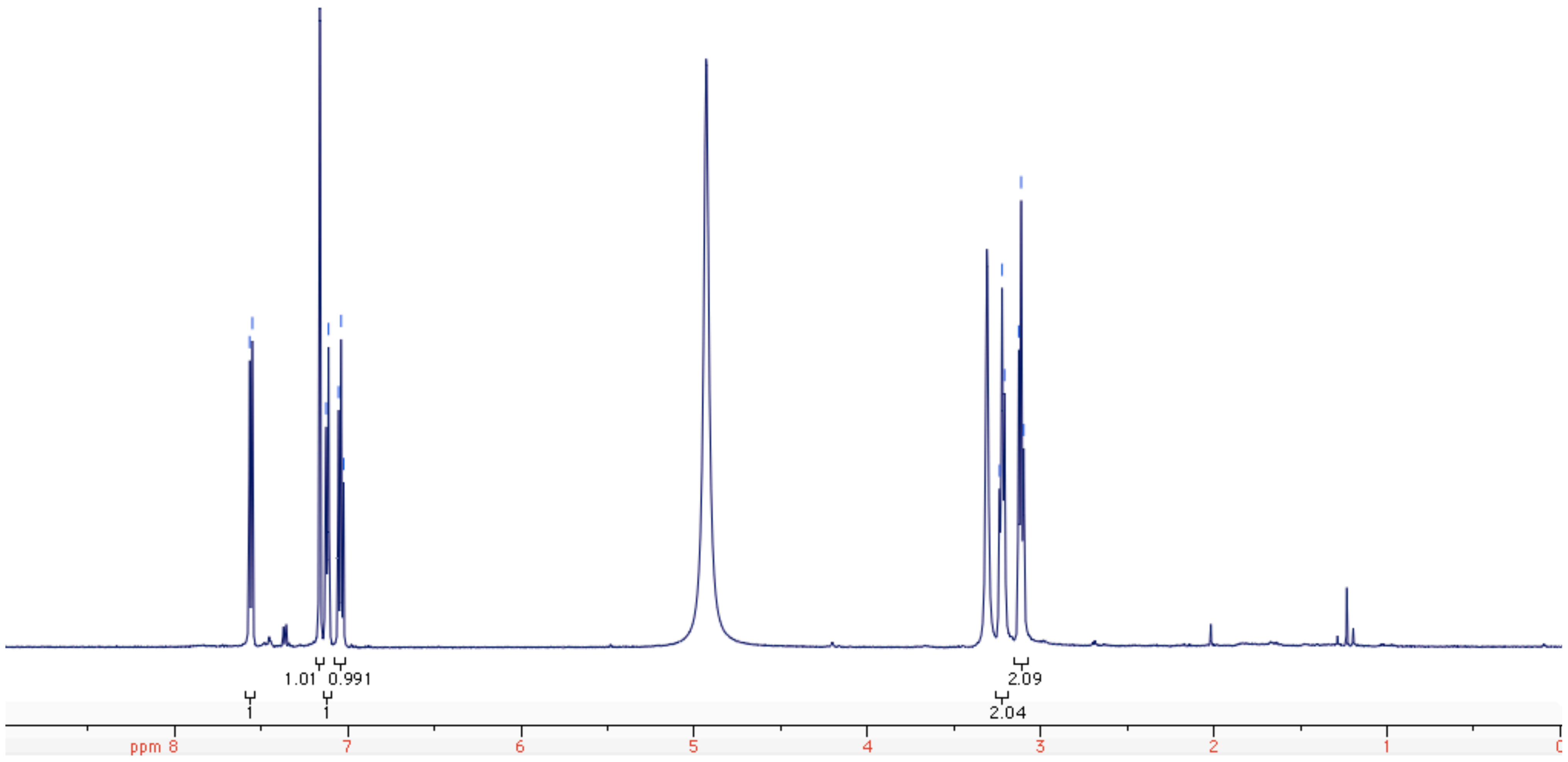

1.01
0.991
1
1
2.09
2.04
ppm 8
7
6
5
4
3
2
1

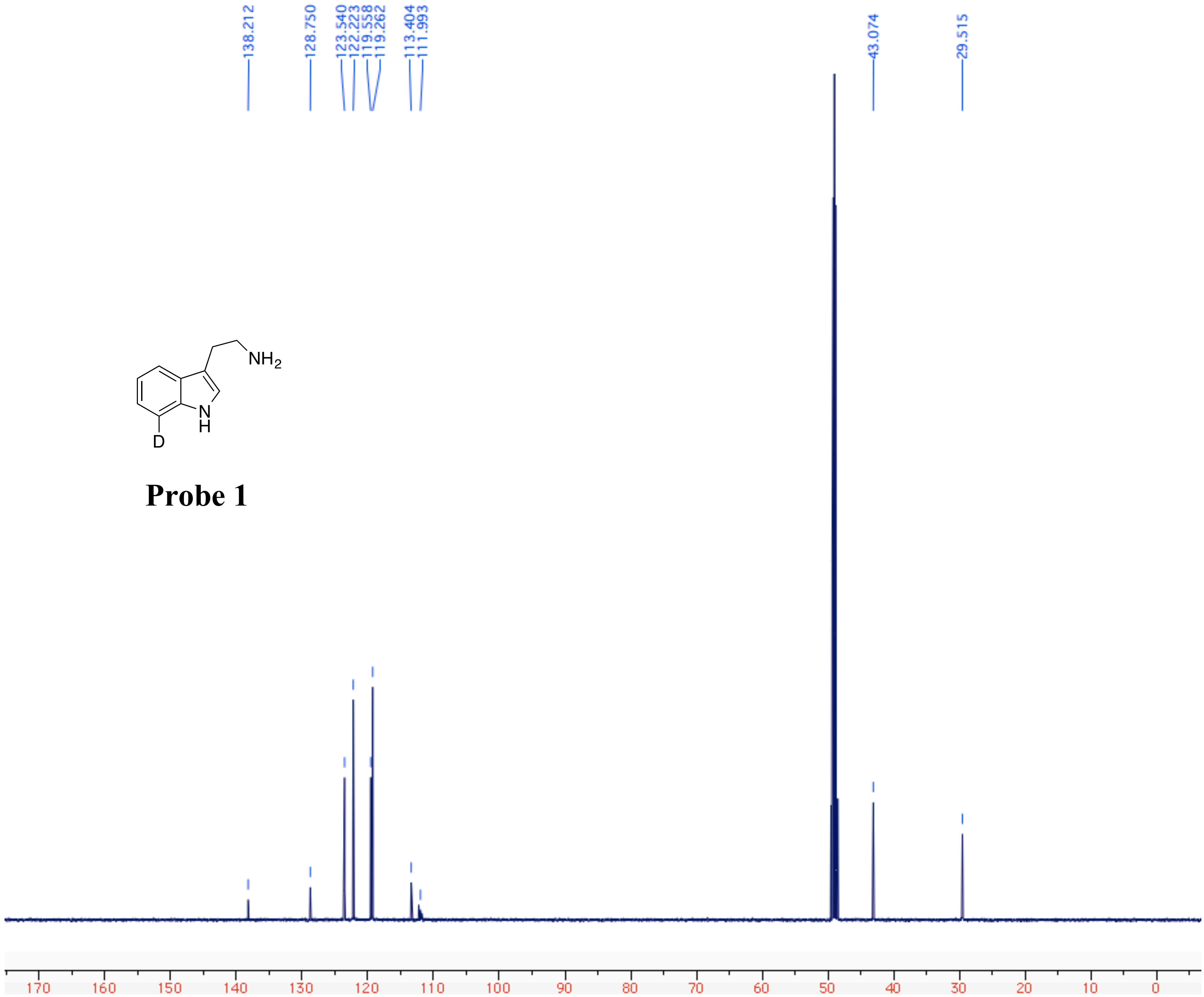
NH2
N
H
D
Probe 1
138.212
128.750
123.540
122.223
119.558
119.262
113.404
111.993
43.074
29.515
170
160
150
140
130
120
110
100
90
80
70
60
50
40
30
20
10
0

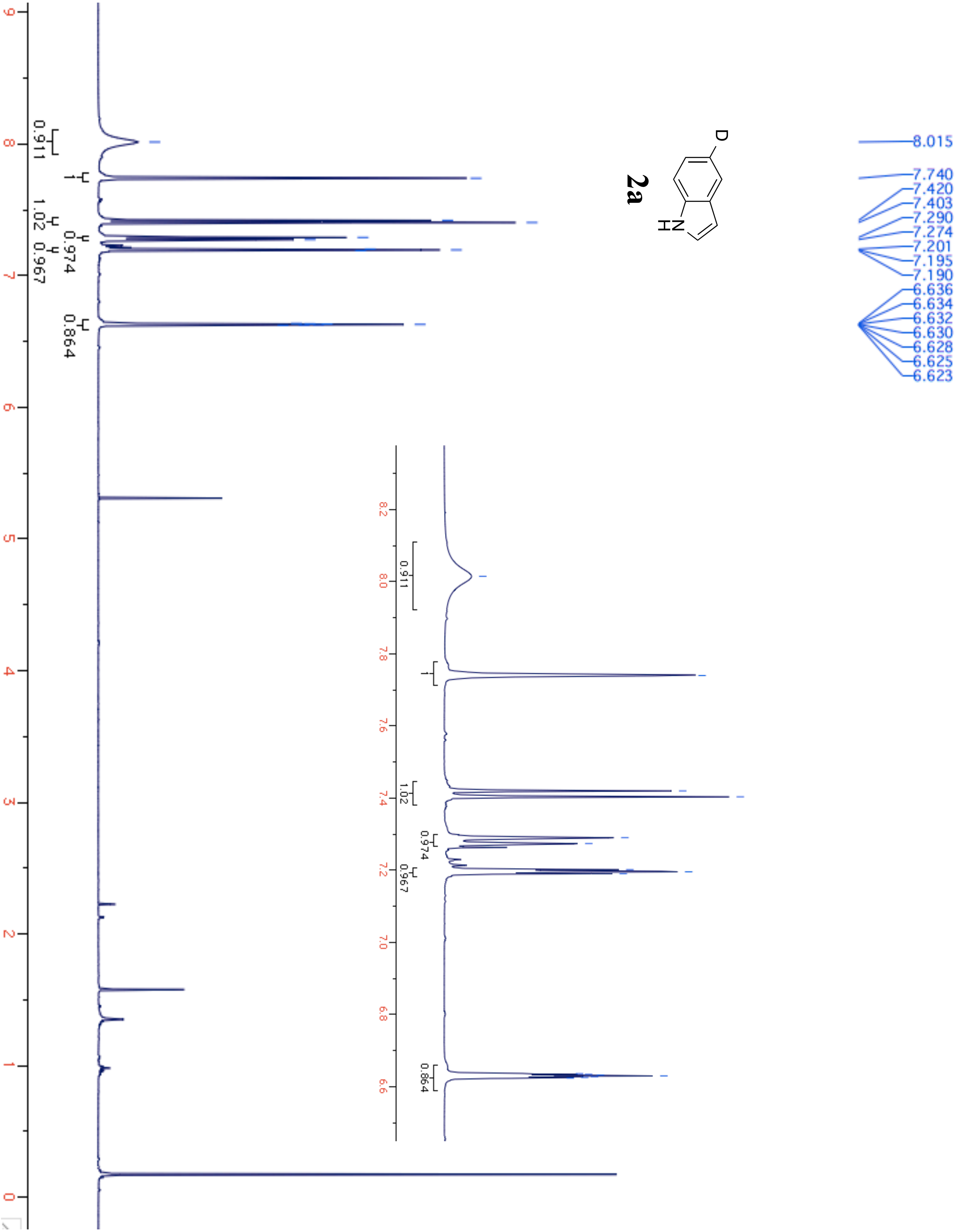

2a
D
N
H
8.015
7.740
7.420
7.403
7.290
7.274
7.201
7.195
7.190
6.636
6.634
6.632
6.630
6.628
6.625
6.623
0.911
1
1.02
0.974
0.967
0.864

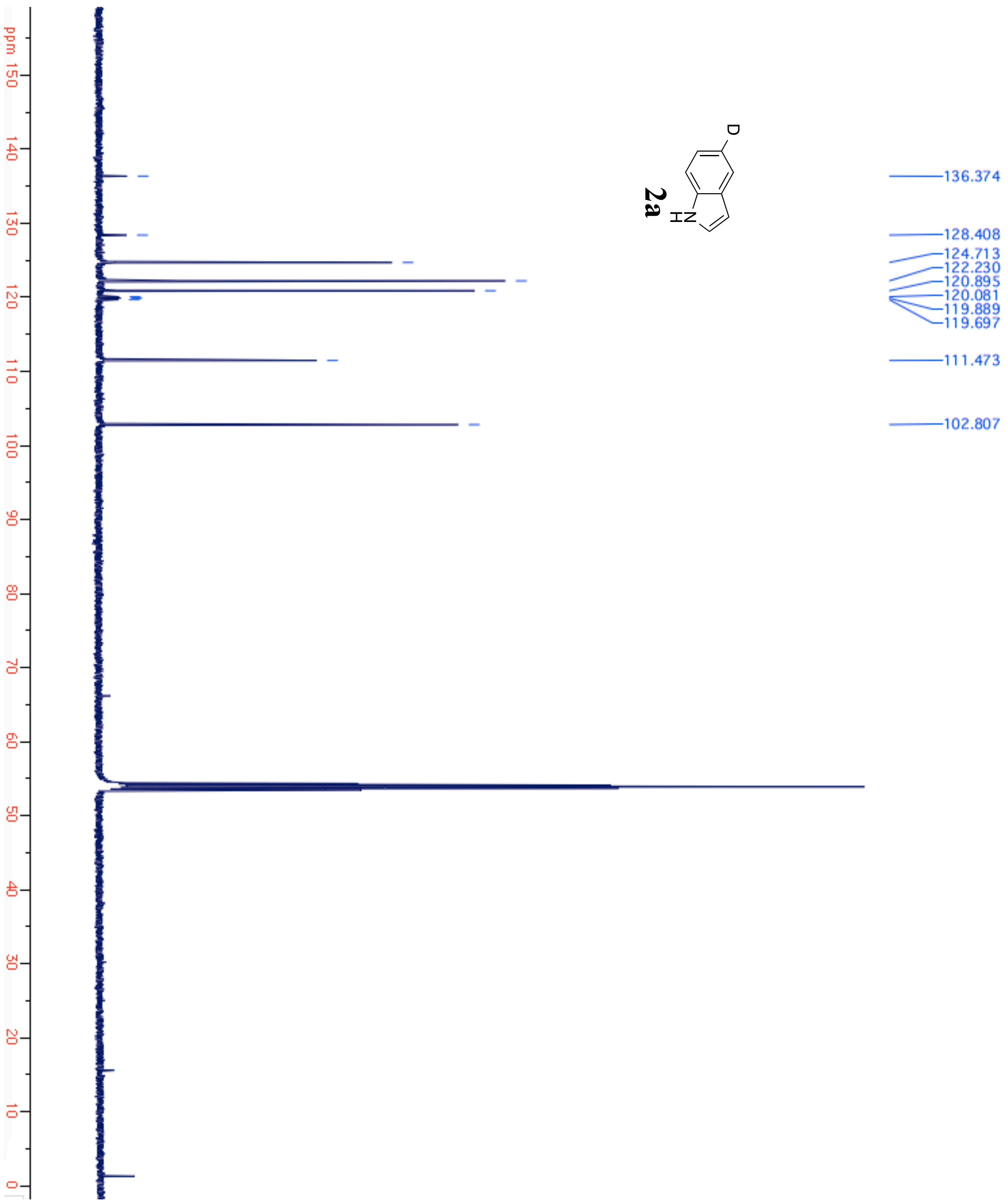
2a
D
N
H
136.374
128.408
124.713
122.230
120.895
120.081
119.889
119.697
111.473
102.807
ppm 150
140
130
120
110
100
90
80
70
60
50
40
30
20
10
0

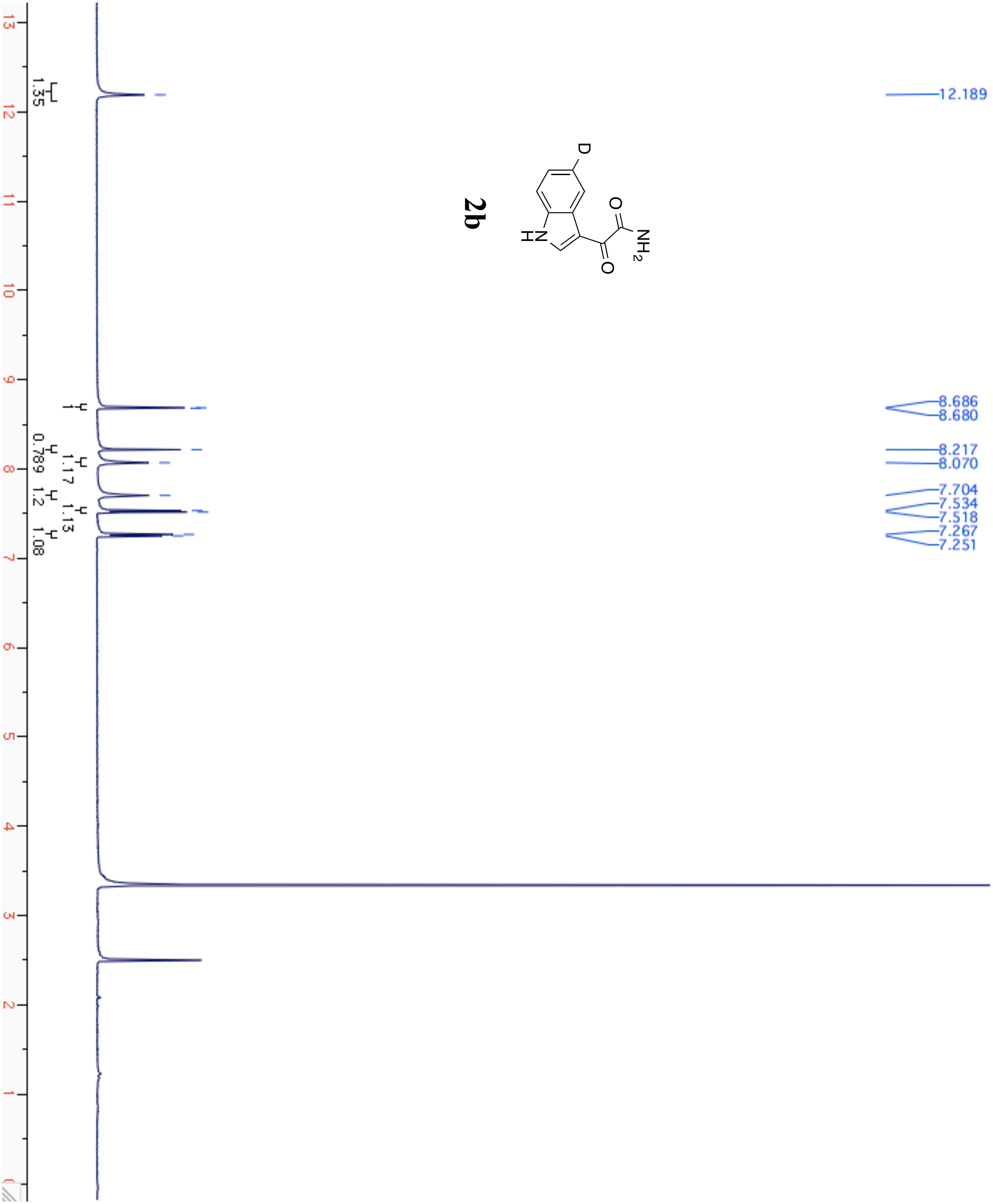
2b
D
O
NH2
O
N
H
12.189
8.686
8.680
8.217
8.070
7.704
7.534
7.518
7.267
7.251
1.35
1
1.17
0.789
1.2
1.13
1.08
13
12
11
10
9
8
7
6
5
4
3
2
1
0

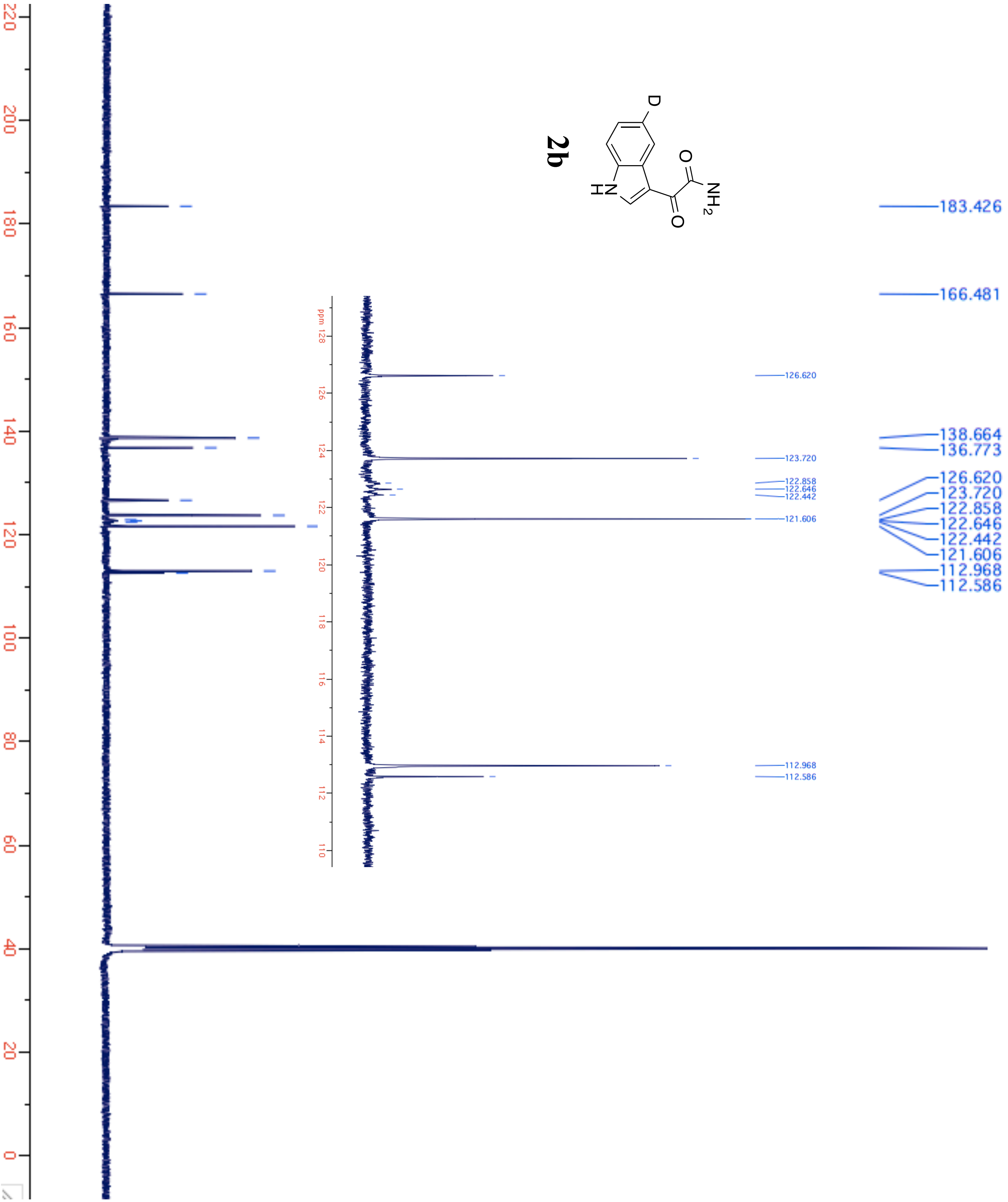
2b
183.426
166.481
138.664
136.773
126.620
123.720
122.858
122.646
122.442
121.606
112.968
112.586
ppm
220
200
180
160
140
120
100
80
60
40
20
0

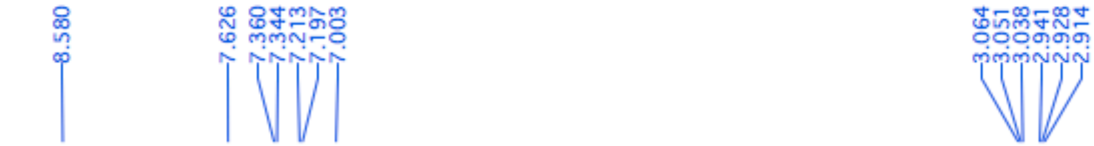
8.580
7.626
7.360
7.344
7.213
7.197
7.003
3.064
3.051
3.038
2.941
2.928
2.914

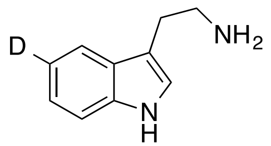
D
NH2
N
H

Probe 2

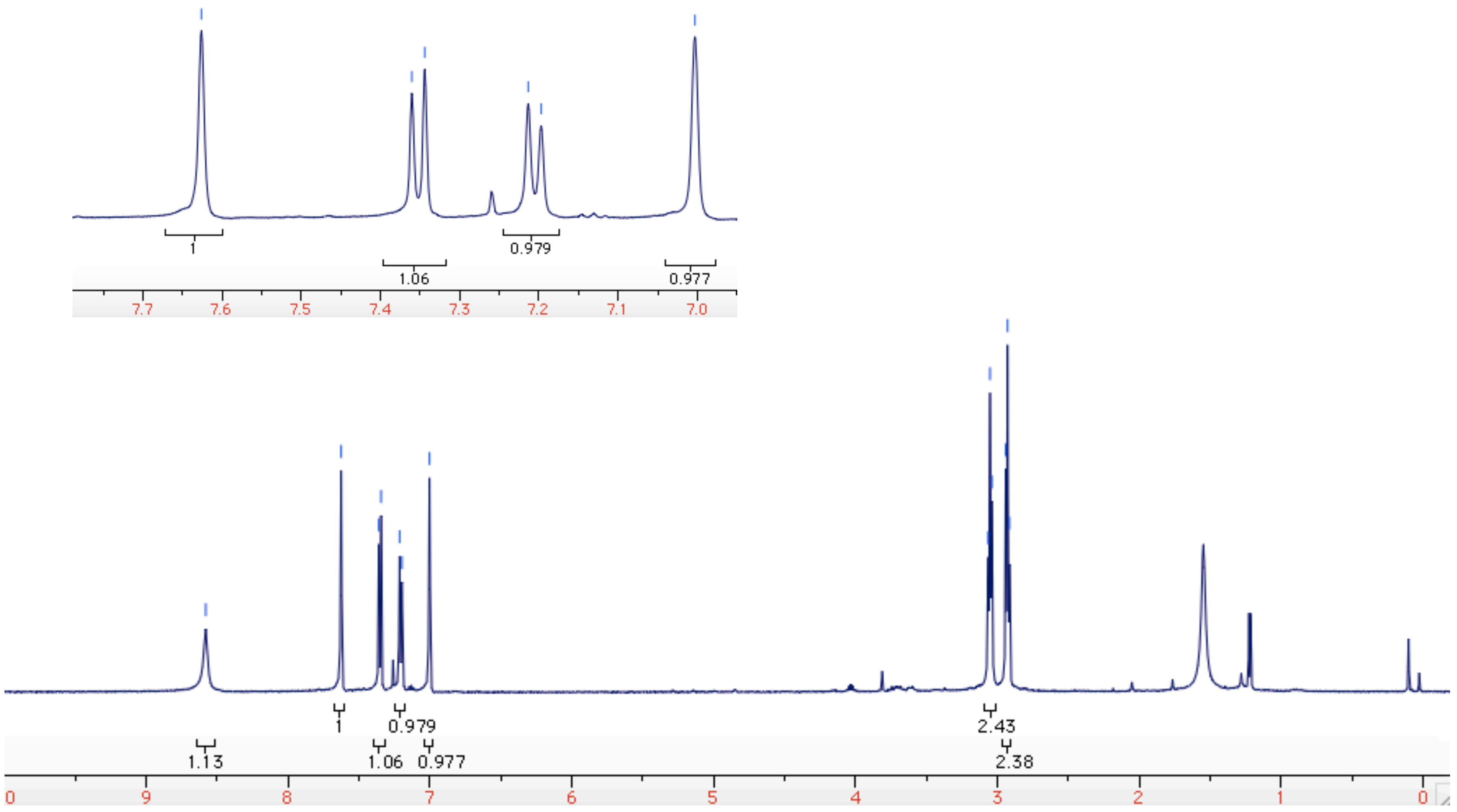
1
1.06
0.979
0.977
7.7
7.6
7.5
7.4
7.3
7.2
7.1
7.0
1.13
2.43
2.38
0
9
8
7
6
5
4
3
2
1
0

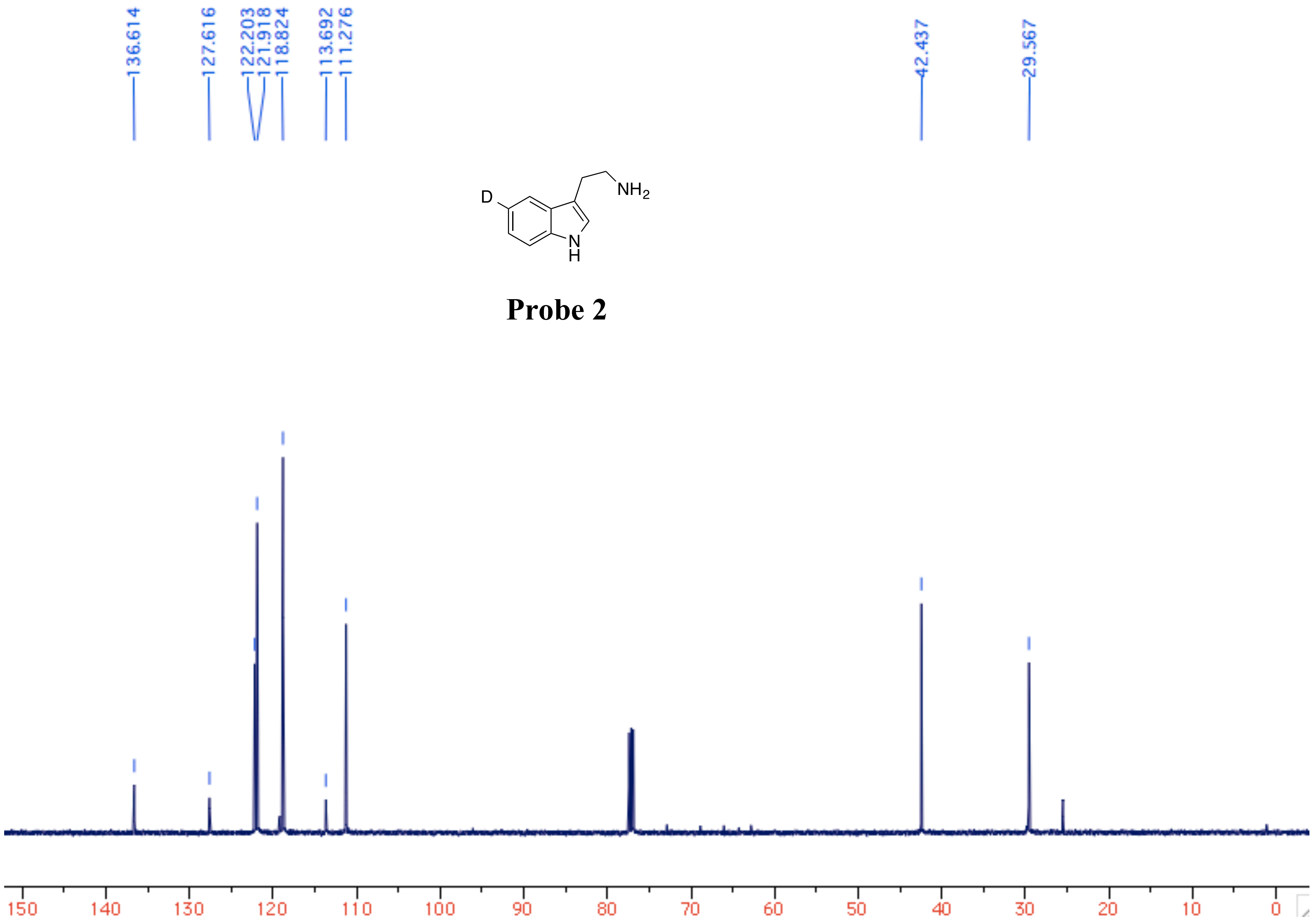
136.614
127.616
122.203
121.918
118.824
113.692
111.276
42.437
29.567
D
NH2
N
H
150
140
130
120
110
100
90
80
70
60
50
40
30
20
10
0

Probe 2

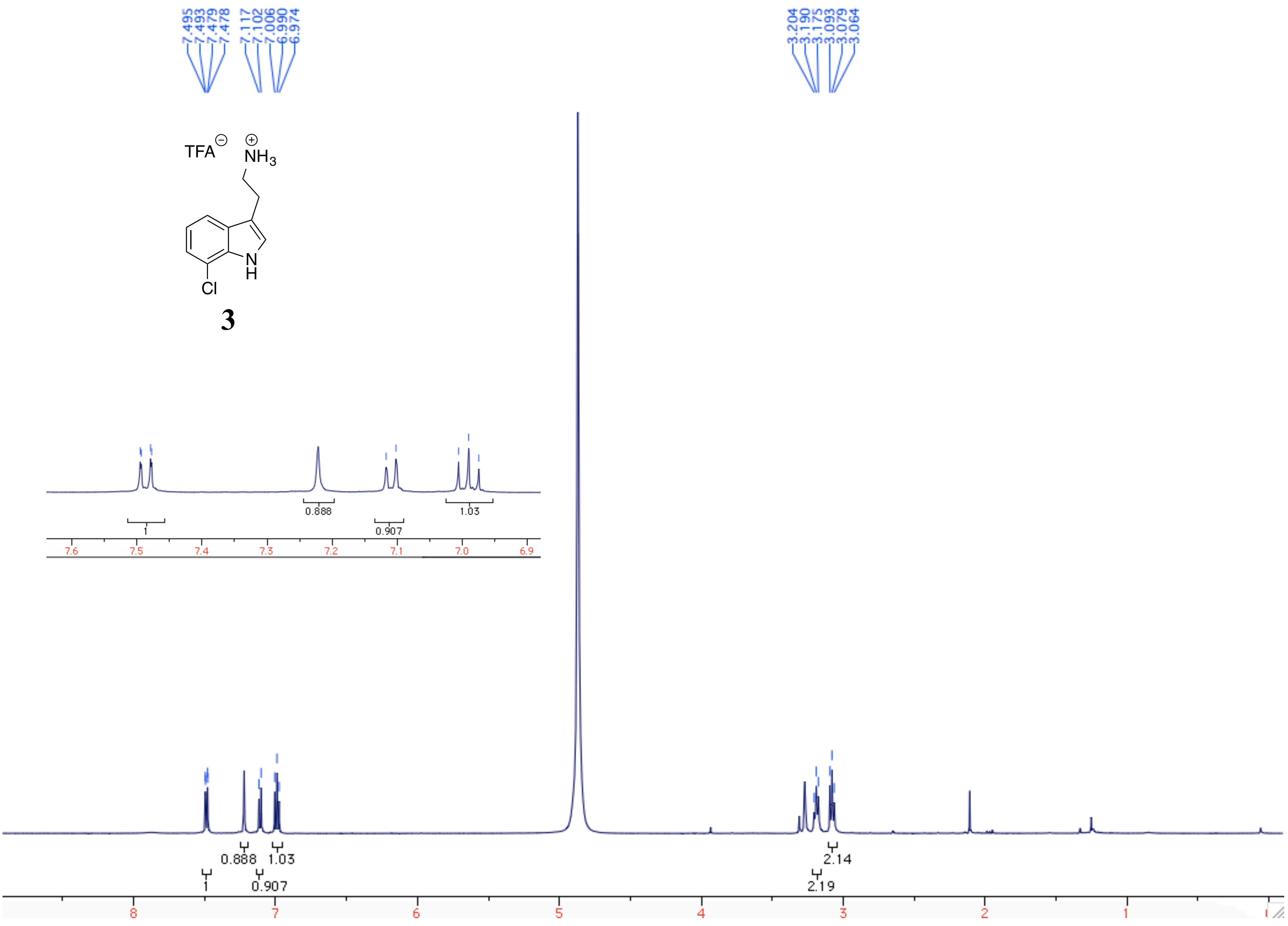

7.495
7.493
7.479
7.478
7.117
7.102
7.006
6.990
6.974
3.204
3.190
3.175
3.093
3.079
3.064
TFA
NH3
N
H
Cl
3
0.888
0.907
1.03
1
2.14
2.19

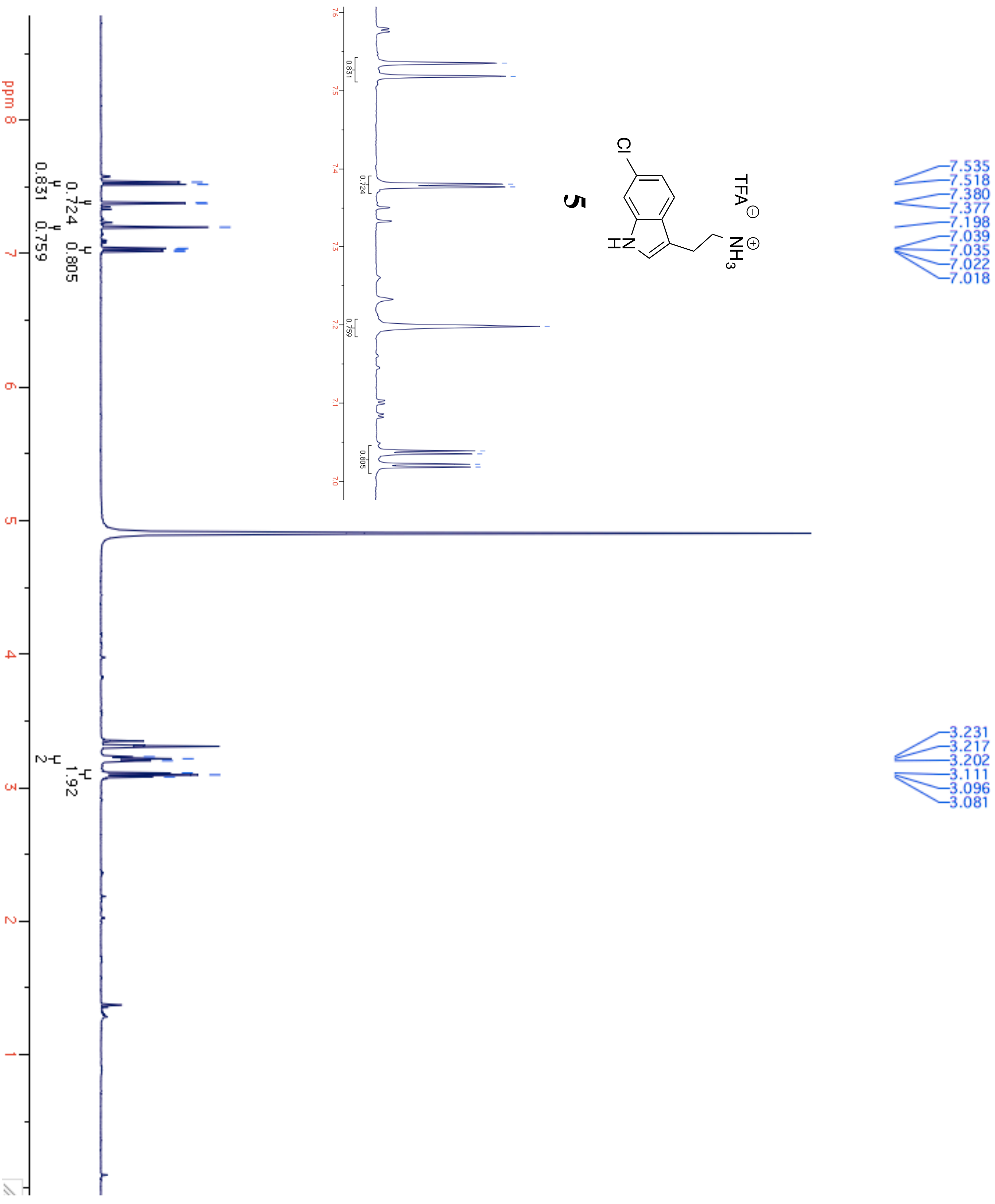
5
TFA
NH3
Cl
7.535
7.518
7.380
7.377
7.198
7.039
7.035
7.022
7.018
3.231
3.217
3.202
3.111
3.096
3.081
0.831
0.724
0.759
0.805
1.92
2
ppm

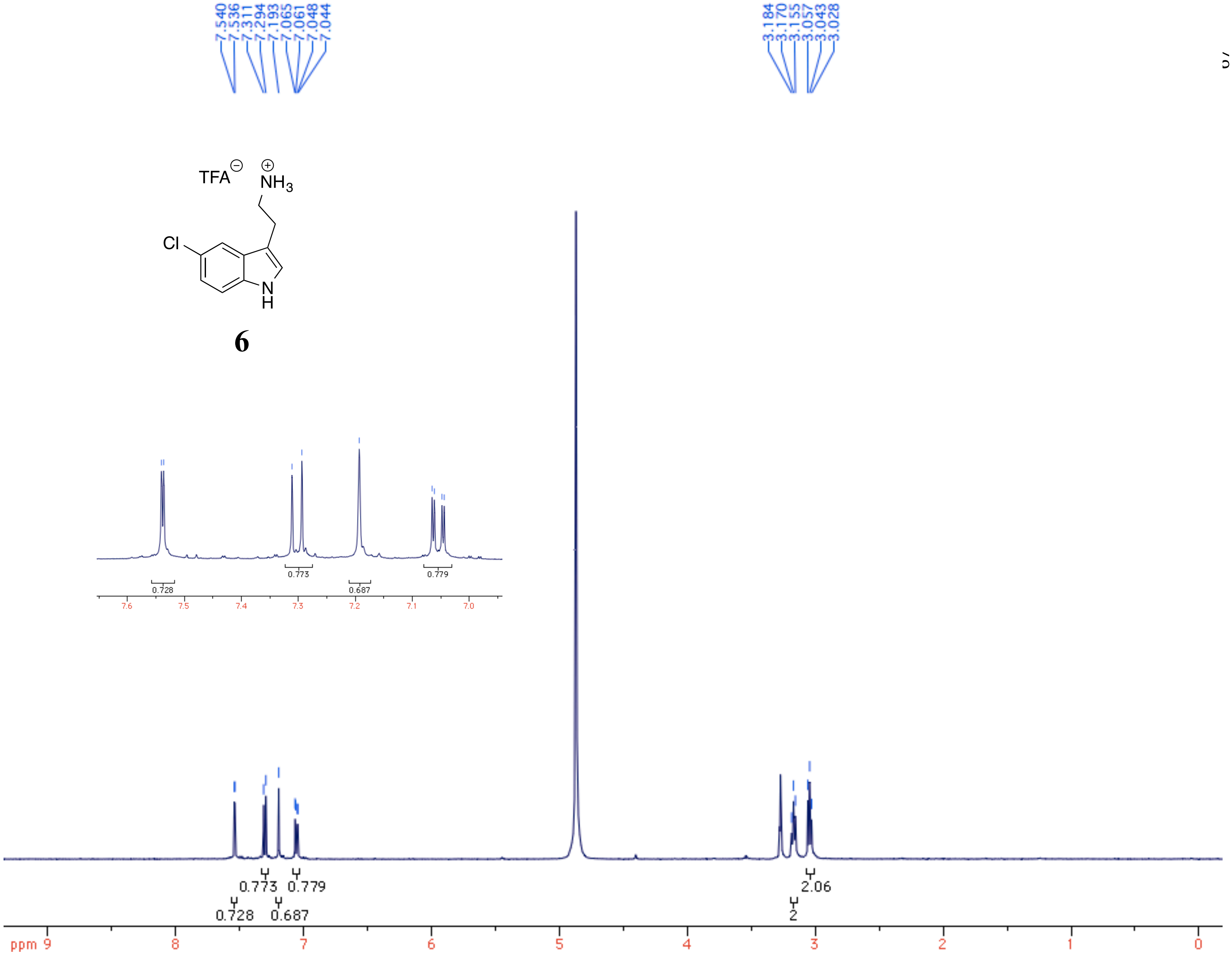
7.540
7.536
7.311
7.294
7.193
7.065
7.061
7.048
7.044
3.184
3.170
3.155
3.057
3.043
3.028
TFA
NH3
Cl
N
H
6
0.728
0.773
0.687
0.779
7.6
7.5
7.4
7.3
7.2
7.1
7.0
0.773
0.779
0.728
0.687
2.06
2
ppm 9
8
7
6
5
4
3
2
1
0

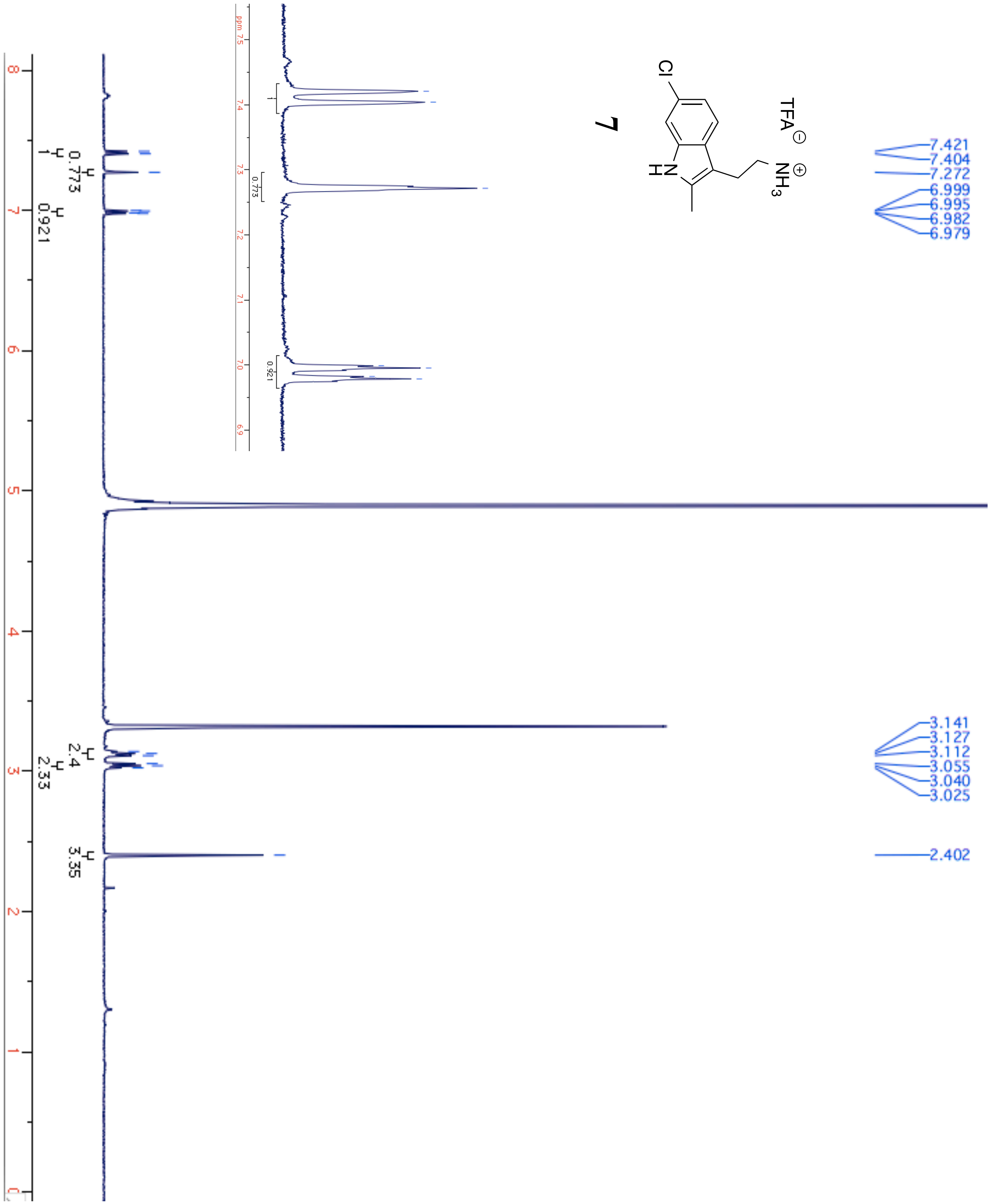

7
TFA⊖ ⊕NH3
Cl
N
H
7.421
7.404
7.272
6.999
6.995
6.982
6.979
3.141
3.127
3.112
3.055
3.040
3.025
2.402
1
0.773
0.921
2.4
2.33
3.35

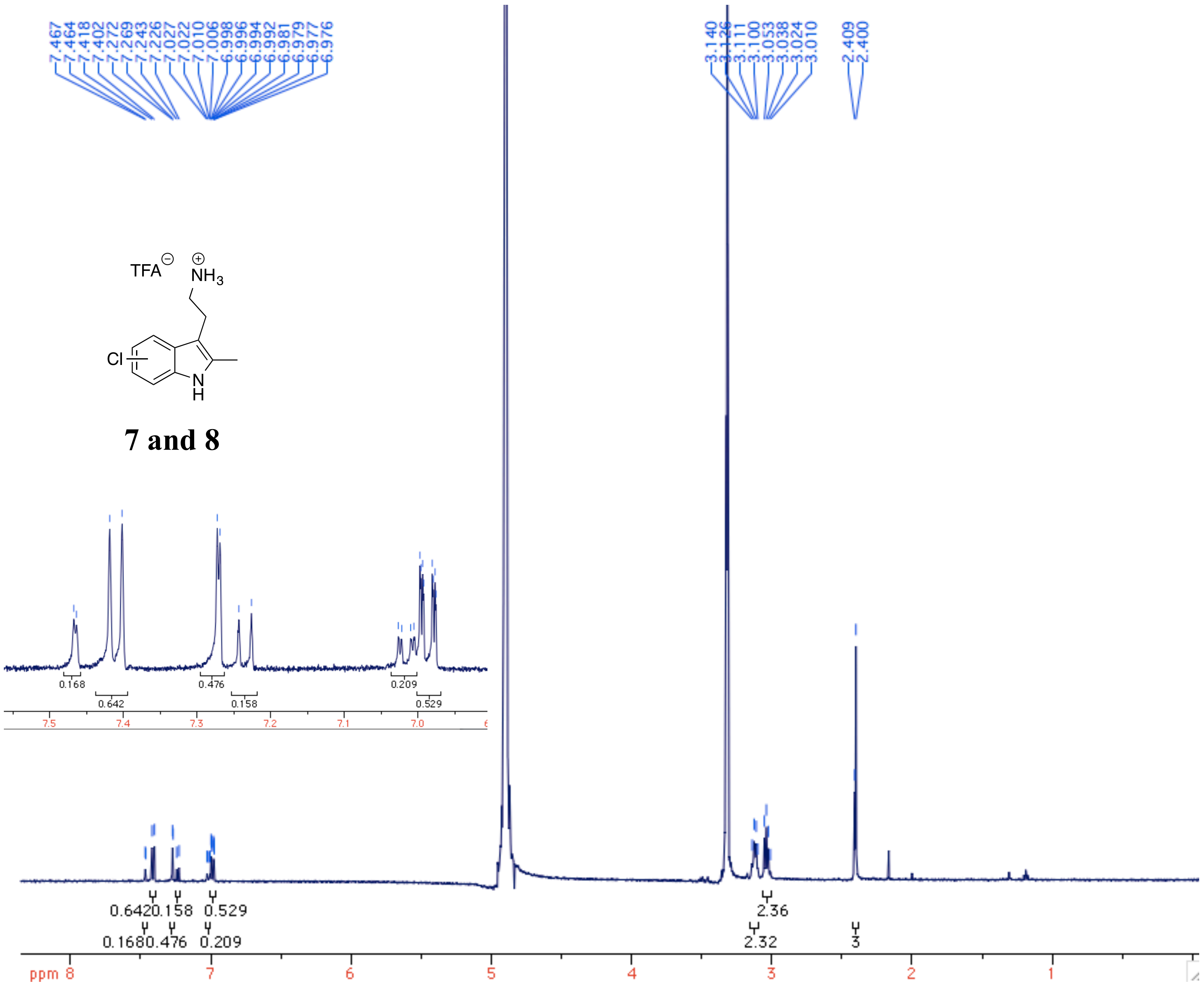
7.467
7.464
7.418
7.402
7.272
7.269
7.243
7.226
7.027
7.022
7.010
7.006
6.998
6.996
6.994
6.992
6.981
6.979
6.977
6.976
3.140
3.126
3.111
3.100
3.053
3.038
3.024
3.010
2.409
2.400
TFA
NH3
Cl
N
H
7 and 8
0.168
0.642
0.476
0.158
0.209
0.529
7.5
7.4
7.3
7.2
7.1
7.0
2.36
2.32
3
ppm 8
7
6
5
4
3
2
1

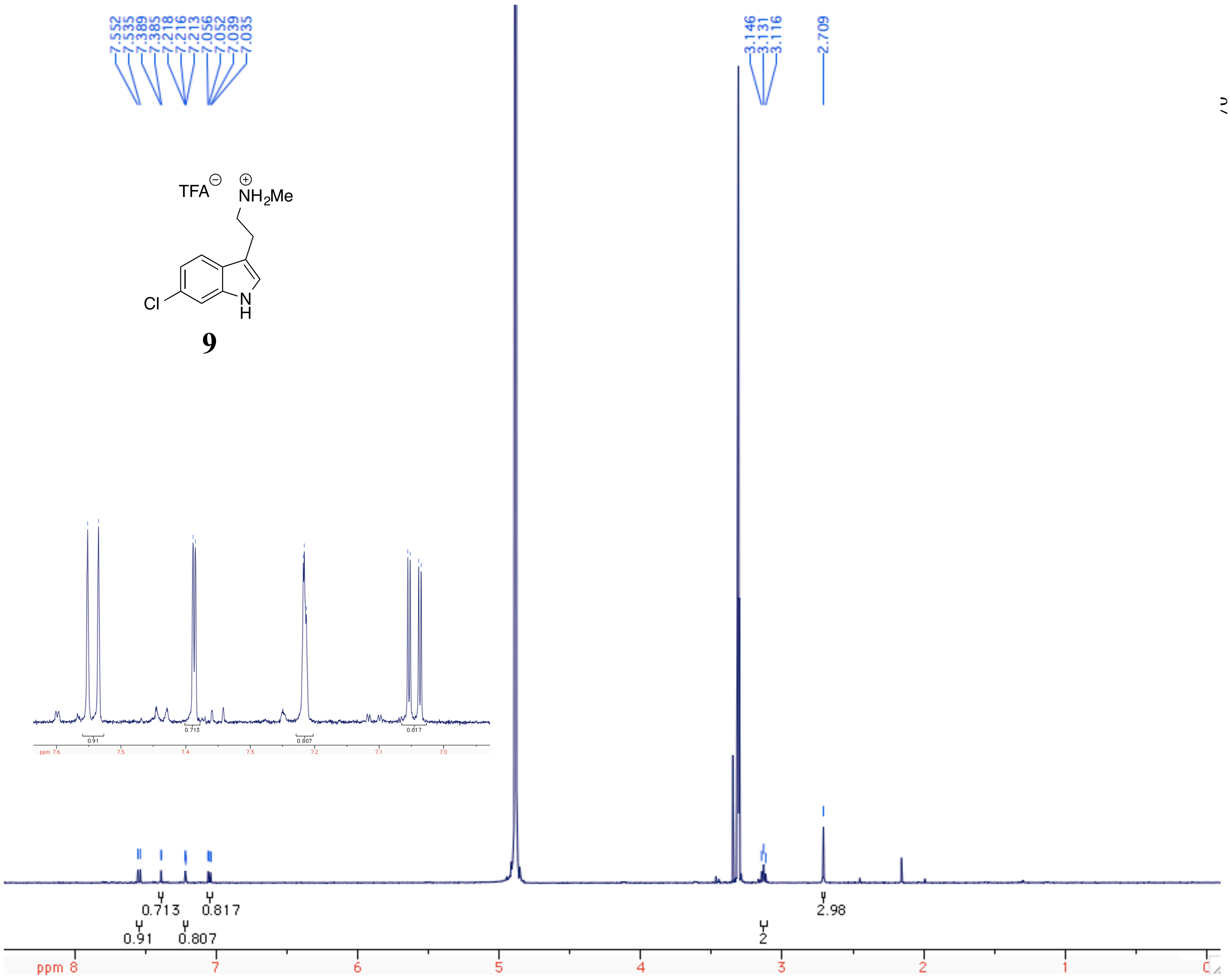
7.552
7.535
7.389
7.385
7.218
7.216
7.213
7.056
7.052
7.039
7.035
3.146
3.131
3.116
2.709
TFA
NH2Me
Cl
N
H
9
0.91
0.713
0.807
0.817
ppm 7.6
7.5
7.4
7.3
7.2
7.1
7.0
2
2.98
ppm 8
7
6
5
4
3
2
1
0

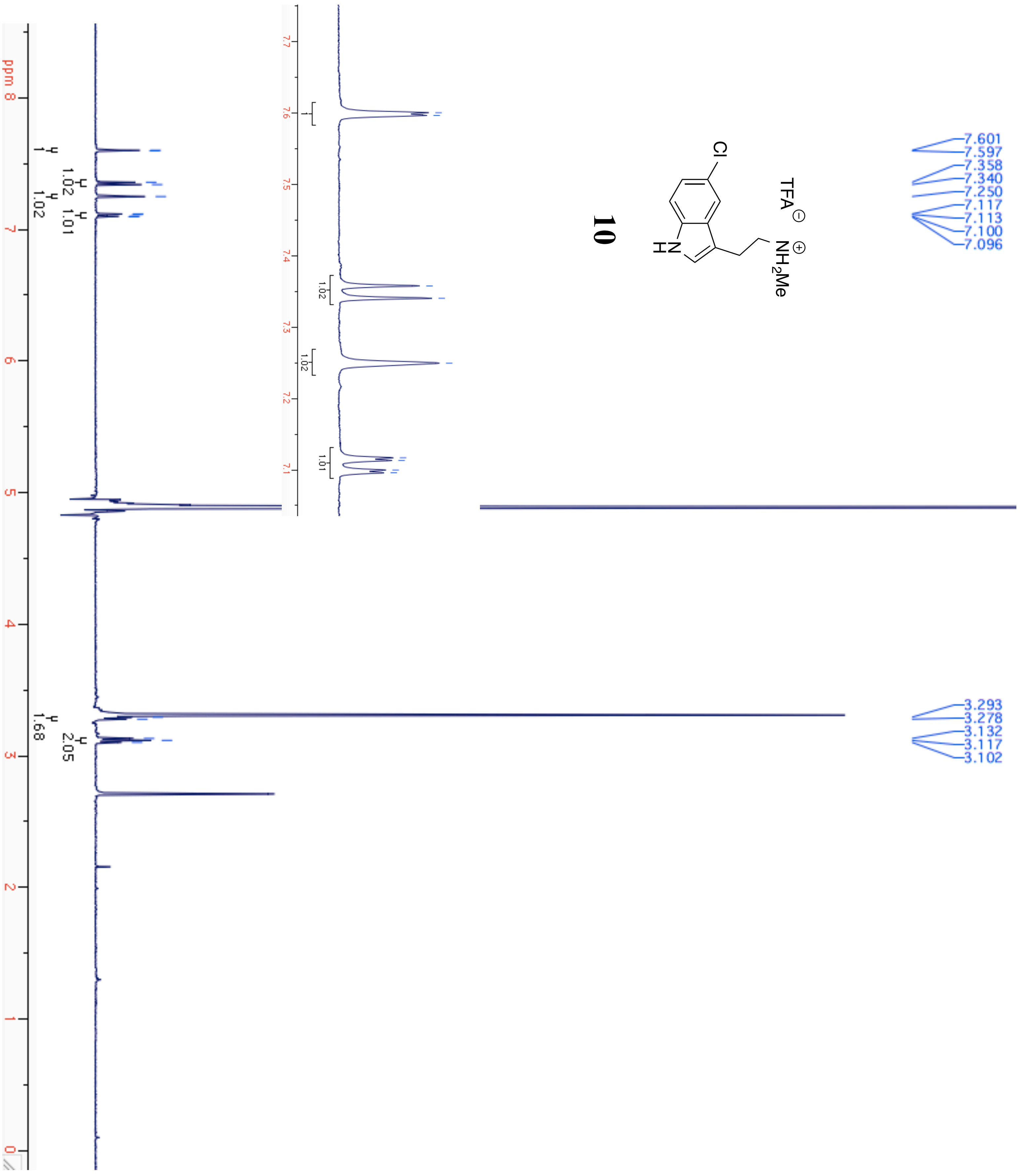
10
TFA
NH2Me
Cl
7.601
7.597
7.358
7.340
7.250
7.117
7.113
7.100
7.096
3.293
3.278
3.132
3.117
3.102
1.02
1.01
1.02
1
1.68
2.05
ppm

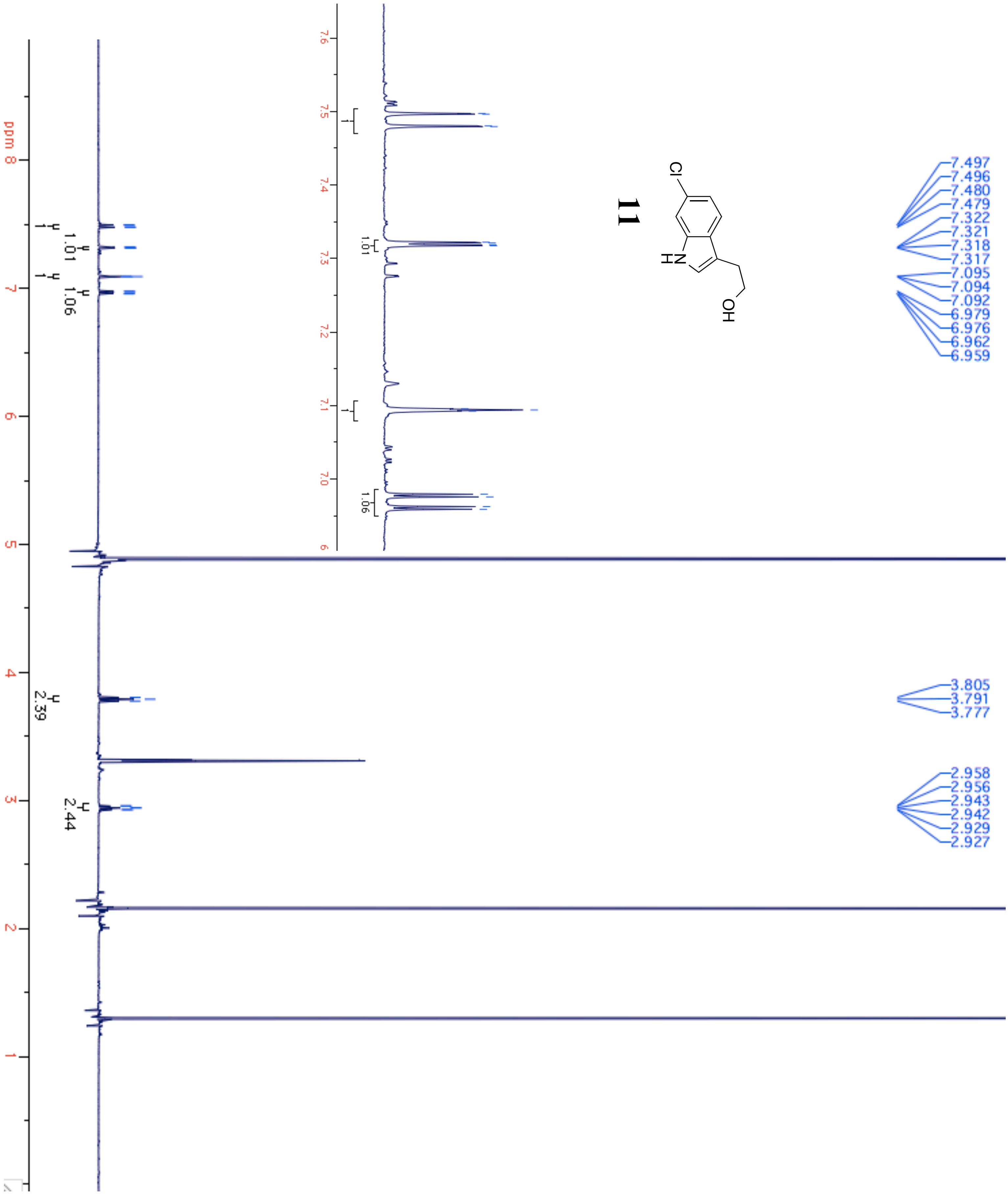
11
Cl
N
H
OH
7.497
7.496
7.480
7.479
7.322
7.321
7.318
7.317
7.095
7.094
7.092
6.979
6.976
6.962
6.959
3.805
3.791
3.777
2.958
2.956
2.943
2.942
2.929
2.927
1.01
1.06
2.39
2.44
ppm

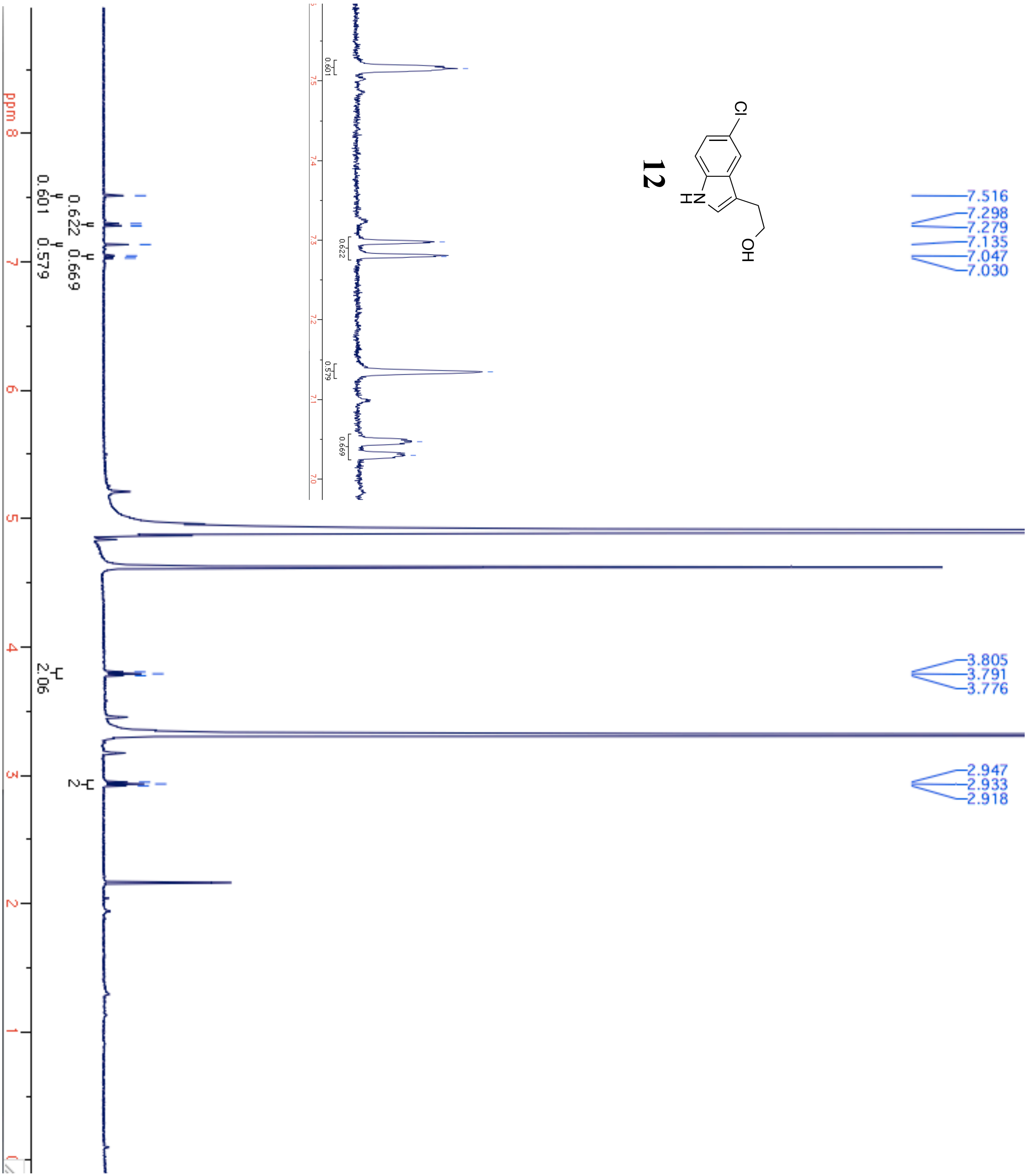
12
Cl
N
H
OH
7.516
7.298
7.279
7.135
7.047
7.030
3.805
3.791
3.776
2.947
2.933
2.918
0.601
0.622
0.579
0.669
2.06
2
ppm 8
7
6
5
4
3
2
1
0

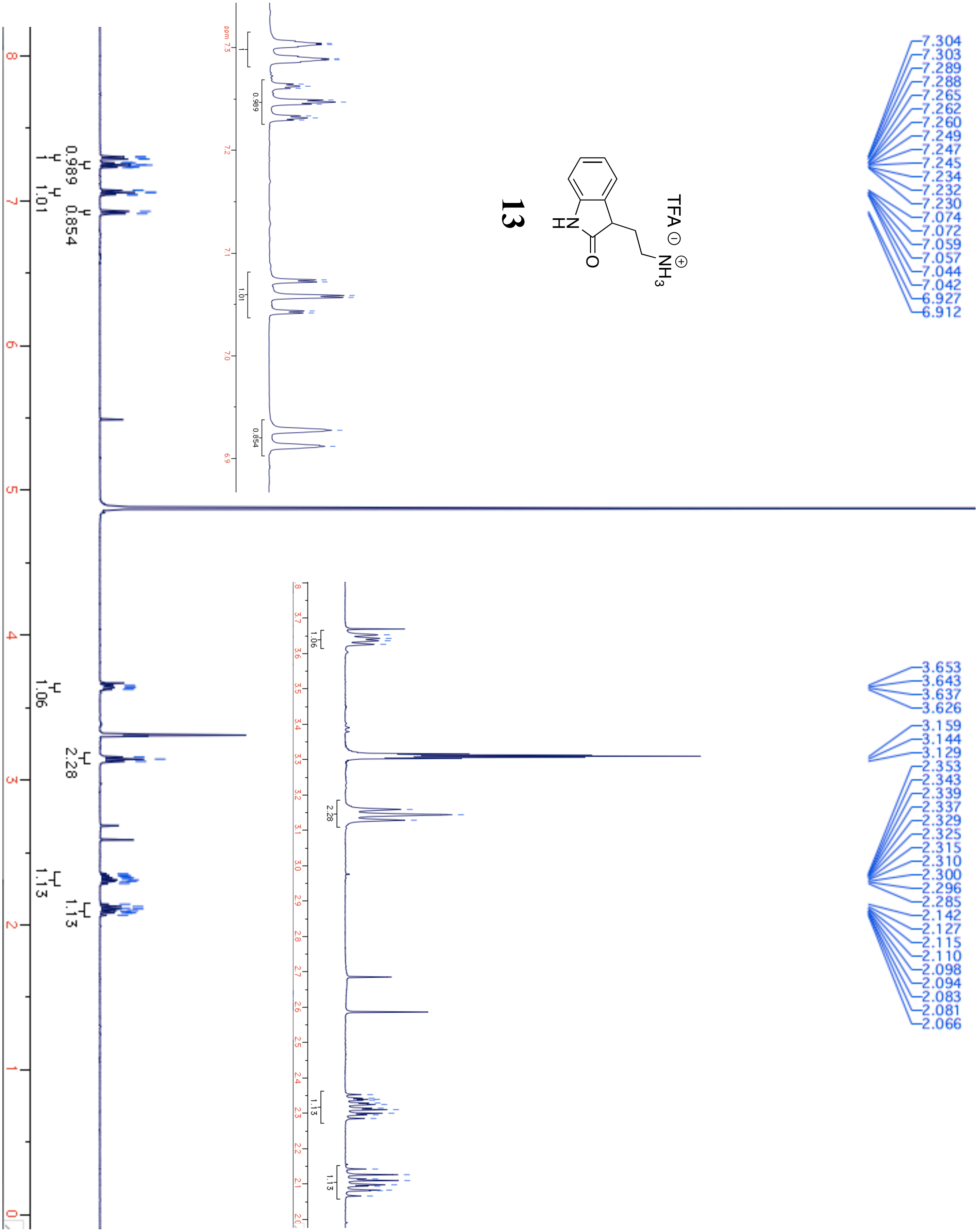
7.304
7.303
7.289
7.288
7.265
7.262
7.260
7.249
7.247
7.245
7.234
7.232
7.230
7.074
7.072
7.059
7.057
7.044
7.042
6.927
6.912
3.653
3.643
3.637
3.626
3.159
3.144
3.129
2.353
2.343
2.339
2.337
2.329
2.325
2.315
2.310
2.300
2.296
2.285
2.142
2.127
2.115
2.110
2.098
2.094
2.083
2.081
2.066
TFA
NH
O
13
1
0.989
1.01
0.854
1.06
2.28
1.13
1.13

References

1. Yeh, E.; Garneau, S.; Walsh, C. T. *Proc. Nat. Acad. Sci.* **2005**, *102*, 3960-3965.

2. Cabrita, L. D.; Dai, W.; Bottomley, S. P. *BMC Biotech. 2006*, 6:12.

3. Parsons, R.L.; Berk, J.D.; Kuehne, M.E. *J. Org. Chem.*, **1993**, *58*, 7482-7489.

4. Payne, J. T.; Andorfer, M.C.; Lewis, J.C. *Angew. Chem.* **2013**, *125*, 5379 –5382.

5. Hara, T.; Durell, S.R.; Myers, M.C.; Appella, D. H. *J Am Chem Soc.* **2006**, *128*, 1995-2004.

6. Nicolaou, K. C.; Krasovskiy, A.; Trépanier, V. E.; Chen, D. Y. K. *Angew. Chem. Int. Ed.* **2008,** *47* (22), 4217-4220.

7. Pham, V.C.; Ma, J.;Thomas, S.J.; Xu, Z.; Hecht, S.M. *J. Nat. Prod.* **2005,** *68,* 1147-1152.

8. Sambrook J, Frisch EF, Maniatis T (**1989**) *Molecular cloning: a laboratory manual. 2*, Cold Spring Harbor Laboratory Press, New York.

9. Gottlieb, H. E.; Kotlyar, V.; Nudelman, A. *J. Org. Chem.* **1997**, *62*, 7512-7515.

10. a. Poor, C. B.; Andorfer, M.C.; Lewis, J.C. *ChemBioChem* **2014**, *15*, 1286 – 1289. b. Payne, J. T.; Poor, C. B.; Lewis, J.C. *Angew. Chem. Int. Ed.* **2015**, 54,4226 –4230.

11. Heckman, K. L.; Pease, L. R. *Nat. Protoc.* **2007**, *2*, 924-932.

12. Caster, Library Evaluation Tool. Downloaded from Prof. Manfred T. Reetz's website, http://www.kofo.mpg.de/en/research/biocatalysis.

13. Robbins, D.W.; Boebel, T.A.; Hartwig, J.F. *J. Am. Chem. Soc.*, **2010**, *132* (12), 4068–4069.

14. Ibaceta-Lizana, J.S.L.; Jackson, A. H.; Prasitpan, N.; Shannon, P.V.R. *J. Chem. Soc., Perkin Trans. 2*, **1987**, 1221-1226.

15. Maresh, J.J.; Giddings, L.A.; Friedrich, A.; Loris, E.A.; Panjikar, S.; Trout, B.L.; Stöckigt, J.; Peters, B.; O'Connor, S. E. *J Am Chem Soc.* **2008,** *130* (2), 710–723.

16. Glenn, W. S.; Nims, E.; O'Connor, S. E. *J Am Chem Soc.* **2011**, *133* (48), 19346-19349.

17. Miyake, F. Y.; Yakushijin, K.; Horne, D. A.; *Org. Lett.*, **2004**, *6* (5), 711–713.

18. Ashtekar KD, et al. *J Am Chem Soc* **2014**, *136*, 13355–13362.

19. Bitto, E.; Huang, Y.; Bingman, C. A.; Singh, S.; Thorson, J. S.; Phillips, G. N. *Proteins: Structure, Function, and Bioinformatics* **2008**, *70*, 289-293.

20. Guex, N.; Peitsch, M.C.; Schwede, T. *ELECTROPHORESIS* **2009,** *30*, S162-S173.

21. Trott, O.; Olson, A. J. *Journal of Computational Chemistry* **2010,** *31*, 455-461.
